# Global anaesthesia practice using inguinal hernia surgery as a tracer condition: a secondary analysis of an international prospective cohort study

**DOI:** 10.1111/anae.16686

**Published:** 2025-09-09

**Authors:** NIHR Global Health Research Group on Environmentally Sustainable Hospitals in Low‐ and Middle‐income Countries, Cortland Linder, Maria Picciochi, Sakina Bhaloo, Ebenezer Amofa, Canada Gaston, Jose Andres Calvache, Sivesh Kamarajah, Palesa Motshabi, Dmitri Nepogodiev, Dhruv Ghosh, Laura Kudrna, Maame Jenny, Muyiwa Rotimi, Telesphore Kabera, Virginia Ledda, Sam Lawday, Craig McClain, Cliff Shelton, Abdul Ghaffar, Adedogni, Allen Jean De La Croix Ingabire, Asad Latif, Nana Boateng, Jean Pierre Nganabashaka, Larissa Cronje, Mogane Palesa, Sylvestre Nzahabwanayo, Pritpal Singh, Revatrams, Rotimi Aaron, Samuel Jerry Cobbina, Sandrine Zola, Suryakiran Sharma, Teena Gill, Tony Thomson, James Glasbey, Janet Martin, Christina George, Aneel Bhangu, Cortland Linder, Maria Picciochi, Sivesh Kamarajah, Bryar Kadir, James Glasbey, Aneel Bhangu, Aneel Bhangu, Abdul Gaffar, Adewale Adisa, Andrew Dove, Christina George, Dmitri Nepogodiev, Elizabeth Li, Ewen Harrison, Fareeda Galley, Ian Thomson, J. C. Allen Ingabire, James Glasbey, Janet Martin, Laura Kudrna, Mwayi Kachapila, Natalie Rowland, Omar Omar, Parvez Haque, Pritpal Singh, Rachel Lillywhite, Richard Lilford, Rob Lillywhite, Samuel Cobbina, Sylvestre Nzahabwanayo, Telesphore Kabera, Tracy Roberts, I. Dajti, Z. Djama, M. Lucchini, R. M. Palacios Huatuco, K. Atherton, A. C. Dawson, E. Lun, F. Aigner, F. Berrevoet, I. Lawani, S. Lawani, C. Bokossa, S. Delibegovic, M. Slavchev, A. F. Sanon, A. Sanou, J. B. Gusa, J. C. Mbonicura, A. Bang, O. Gabom, C. Nwegbu, A. Brar, J. Martin, M. M. Modolo, M. Olivos, J. A. Calvache, J. Mihanovic, N. Gouvas, A. Yiallourou, B. East, S. Batista, R. Rivas, E. P. Lincango, S. Emile, A. B. Aregawi, A. P. Arnaud, N. Boumas, Z. Demetrashvili, H. Lederhuber, M. W. Löffler, A. E. Agbeko, N. B. Sam, S. Tabiri, F. Agyei, F. E. Gyamfi, S. Mohammed, I. Katsaros, G. Tsoulfas, L. Bains, J. Dhiman, D. Ghosh, P. D. Haque, A. Suroy, S. Ramjit, G. Marom, F. Pata, G. Gallo, F. Ayasra, I. R. Fakhradiyev, I. H. S. Hamdun, A. Iqbal, E. Mbanzabugabo, M. Elhadi, A. Gulla, L. Samison, M. Nyirenda, R. Nyirenda, A. C. Roslani, B. Bengaly, J. Psaila, L. Martinez, A. Ramos‐De la Medina, P. R. Nashidengo, M. McGuinnes, D. Wright, A. Ousseini, A. Adisa, A. O. Ademuyiwa, T. Risteski, Z. Al Balushi, B. Dawud, A. AlSharqi, F. Ali, A. U. Qureshi, H. Abu‐Arish, H. Gomez‐Fernandez, J. M. Faylona, M. D. Sacdalan, W. Krawczyk, J. G. Goncalves‐Nobre, M. Sampaio‐Alves, I. Santos, I. Negoi, A. Butyrskii, J. C. Allen, F. Ntirenganya, I. Fortune, J. Kosir, N. Parker, K. Chu, A. Minaya Bravo, D. Wickramasinghe, U. Jayarajah, M. Elmujtaba, M. Nikberg, E. Gialamas, M. Alshaar, M. Nkoronko, A. Isik, I. Mubesi, J. NgKamstra, O. Bahsas‐Zaky, E. Agastra, I. Dajti, R. Belouz, Z. Djama, A. Mouffokes, M. E. Muriel, R. M. Palacios Huatuco, M. Santillan, A. Duro, J. I. Valenzuela, D. A. Pantoja Pachajoa, G. Romero reyna, C. M. Florián Villa, M. Gosselink, Y. H. Lam, A. Nguyen, J. A. Duffield, A. Frankel, S. Bowman, D. Mitchell, H. Iswariah, S. Abeykoon, S. Gananadha, S. Gananadha, S. Salindera, S. Stevens, A. C. Dawson, M. Issa, E. Wong, L. Bromley, E. Wong, K. Jaffry, M. Bickford, E. Wong, R. Nataraja, R. Hodgson, A. Fox, M. Wichmann, A. Davis, S. Zhang, M. Ishak, R. Liang, P. Tang, R. Liang, M. Park, C. Cornwell, E. Page‐Taylor, A. Hameed, R. McGee, I. Königsrainer, F. Aigner, S. Mikalauskas, L. Havranek, A. Binder, S. Islam, M. P. Singh, G. Gbessi, H. Aouagbe Behanzin, M. Agbadebo, E. Bara, A. B. Yevide, T. K. Hessou, A. M. Hodonou, I. Lawani, Z. Matkovic, N. Lalovic, J. Miskovic, M. Salibašić, A. Cerovac, A. Tursunovic, C. Panis, T. Ivanov, M. Karamanliev, R. Donchev, D. Hadzhiev, T. Yotsov, E. Hristova, A. F. Sanon, J. C. Mbonicura, N. Diomede, J. Gusa, S. Stock, O. Ndizeye, R. Spence, S. Lee, S. Lee, M. M. Modolo, C. J. Perez Rivera, S. Sierra, D. S. Garcés Palacios, J. A. Calvache, J. Mihanovic, G. Augustin, A. Yiallourou, J. Moravik, A. Al Kaddah, Z. Musilová, M. Schön, J. Roman, B. East, R. Rivas, G. Abouelnagah, D. Attia, G. Abouelnagah, A. Maher, A. Kedwany, S. Abdelmohsen, R. Adel Diab, A. Al‐Mallah, H. Taher, H. Abozied, A. S. M. Abdelrahman, M. ElFiky, N. Shehata, B. Fahmy, H. Elghadban, E. Adel Mahmod Sultan, M. Omar, E. A. Ahmed, A. M. Elkhouly, A. Asla, F. Terefe, A. Yeshitila, M. Taeme, A. Demessie, A. B. Aregawi, N. S. Bayleyegn, B. Sime, A. Police, A. Castaldi, E. Reitano, N. Boumas, Z. Demetrashvili, C. Kamphues, D. Hackner, U. Ronellenfitsch, J. Rolinger, D. Reim, N. Börner, J. Goedeke, A. E. Gut, M. Janda, J. De Deken, M. W. Löffler, R. Armah, A. Bediako Bowan, E. Kafui Ayodeji, E. A. Arkoh, F. E. Gyamfi, A. Davor, M. T. Morna, N. Agboadoh, E. A. Nachelleh, A. E. Agbeko, S. Mensah, P. Taah‐Amoako, K. Collins, A. S. Seidu, B. K. Seshie, G. Ansong, K. Kambouri, A. Kyriakidis, D. Korkolis, N. Memos, D. Kelgiorgi, C. Chouliaras, E. Fradelos, N. Michalopoulos, N. Dimitrokallis, A. Paspala, P. Christodoulou, E. C. Tampaki, D. Schizas, K. Kontzoglou, M. Spartalis, M. Billis, N. Tsakiridis, A. Karakosta, D. Panagopoulos, G. Koukoulis, G. Christodoulidis, K. Bouchagier, V. Mousafeiris, A. Papadopoulos, L. Katsiaras, N. Zampitis, O. Ioannidis, M. Drogouti, C. Kaselas, D. Lytras, M. Aguilera‐Arevalo, L. TaléRosales, S. T. Torres Rodríguez, N. Krishnappa, S. Kumar Venkatappa, T. S. Mishra, Y. Sakaray, R. Kottayasamy Seenivasagam, R. Sharma, R. Gupta, M. Luthra, T. Longkumer, A. Chhabra, T. Doma Bhutia, M. Pathak, J. Rathod, M. K. Agrawal, D. Jain, N. K. Chaudhry, A. Mathew, P. Alexander, V. Kumar, R. D. Sharma, B. Sarang, D. Singh, N. Gupta, S. Kulkarni, T. Rashid, L. Bains, T. Iahmo, A. Kumar, M. Kumar, R. Abhinaya, V. S. Jha, D. Dugar, S. Basu, K. Singh, C. Mahakalkar, F. Q. Parray, J. A. Kalyanapu, M. Chisthi, A. Kavalakat, B. Roopavathana, N. Yousefzadeh Kandevani, M. Pourfridoni, R. Raheem Attallah Al_obaidy, Z. Alkhuzaie, M. A. Al‐Juaifari, S. Ramjit, R. Tummon, S. Abu Salem, R. Sulce, P. M. Cicerchia, E. Marra, M. Rottoli, J. Andreuccetti, E. Locci, F. Cappellacci, N. Cillara, E. Abate, F. Ascari, S. Romano, M. Veroux, B. Nardo, D. Sasia, G. Baronio, N. Fabbri, J. Martellucci, G. Canonico, V. Lizzi, F. D'acapito, D. Merlini, A. Barberis, M. F. Amisano, A. Luzzi, F. Palmieri, C. L. Bertoglio, E. Baldini, M. Ceolin, P. De Nardi, M. G. Piacentini, F. Ferrara, F. Brucchi, F. Di Marco, N. Tamini, P. Anoldo, R. Patrone, F. Selvaggi, G. Bellio, P. Venturelli, L. Conti, L. Morelli, S. M. M. Basso, F. Biolchini, C. Marafante, A. Antinori, M. Campanelli, P. Lapolla, G. Palomba, L. Cardinali, E. Andolfi, L. Verre, G. Poillucci, E. Pontecorvi, S. Novello, M. Santarelli, L. Cobellis, G. Ietto, F. Pederiva, A. Iacomino, D. Verdi, A. Broglia, P. Cianci, M. Angelucci, G. Calini, H. Yonekura, S. Alananzeh, A. Qasem, Y. Alawneh, S. Al‐Tahayneh, A. Khamees, M. E. H. Albanna, R. K. Z. Almahadin, M. Mahafdah, O. Mansour, M. Mubarak, A. Alrababah, M. Kulimbet, I. Fakhradiyev, R. Parker, D. Rahme, H. Hamdar, W. Ebrahim, M. Saleh, S. Alsaeiti, R. Michael, A. Bojazyah, H. Bileid Bakeer, M. Abudabbous, N. Albahloul, A. Egdeer, H. Embarek, M. Abdelkabir, H. Idheiraj, R. Salim, K. Ayad, A. Alragheai, S. Egreara, S. Timmalah, N. Lahmer, N. Ben Hasan, D. Venskutonis, A. Dauksa, A. Gulla, F. Rasoaherinomenjanahary, M. S. Mohd Shah, R. Noor, S. N. Loke, M. A. Yunus, H. Amin‐Tai, K. S. Dembele, J. Psaila, C. M. Nuño‐Guzmán, L. A. Flores Chávez, C. M. Nuño‐Guzmán, G. Yanowsky‐Reyes, A. Gonzalez Ojeda, G. Ambriz González, E. E. Lozada Hernandez, M. Trejo‐Avila, C. Moreno‐Licea, A. Navarrete‐Peón, M. Noguez Castillo, A. Ramos‐De la Medina, I. Gouazar, A. Ouachhou, P. R. Nashidengo, S. Rennie, M. Haimona, F. Fadzlullah, L. Paterson, A. Adeyeye, J. Olaogun, N. Oloko, P. Agbonrofo, A. Abiodun, U. Ezomike, S. A. Sani, T. A. Lawal, A. O. Lawal, A. I. Okunlola, O. M. Williams, A. Adisa, T. Mohammed, P. Elemile, I. I. Aremu, L. Abdur‐Rahman, J. G. Makama, I. U. Garzali, T. T. Ibiyeye, O. H. Ekwunife, O. H. Ekwunife, O. Ojewuyi, I. Ogundele, M. Daniyan, T. Risteski, H. Al‐Aamri, A. H. Al Sharqi, M. Al Hinai, S. Ahmed, S. H. Waqar, M. Shahid, F. Ashraf, A. N. Syed, A. S. Ammar, K. Hayat, N. Talat, W. Mabood, H. W. Bhatti, M. Usman Malik, B. Mohamad, A. Alwali, A. AbuNemer, S. Alijla, H. Ayesh, H. Abu‐Arish, D. Rabaia, A. Jaber, M. MohammedAli, A. Attili, O. M. Cuenca Torres, V. Panduro‐Correa, L. Fuentes Rivera Lau, C. F. Huaroto Landeo, G. Mendiola, Y. Carpio Colmenares, C. Shiraishi Zapata, R. Díaz‐Ruiz, Ł. Nawacki, M. Kisielewski, Z. Orzeszko, M. Matyja, W. Krawczyk, M. Walędziak, J. K. Zajac, F. Brzeszczyński, S. Henriques, J. Frazão, S. Gaspar Reis, A. R. Mateus Loureiro, M. Reia, J. Pinho, D. G. Alves, R. Silva Borges, E. Borges, M. Nunes, A. Faustino, G. Fialho, J. Dias‐Ferreira, M. Santos, J. Marques Antunes, H. Devesa, J. Ricardo, R. Branquinho, J. Fernandes, J. Pereira‐Macedo, B. Vieira, C. E. Guldogan, S. T. Makkai‐Popa, F. Grama, E. A. Toma, I. Negoi, M. Muresan, V. Kakotkin, A. Bedzhanyan, S. Katorkin, A. Butyrskii, V. Ten, N. Christian, C. Mpirimbanyi, A. Costas‐Chavarri, N. Alzerwi, A. Shabkah, A. Nawawi, N. Alzerwi, S. Chowdhury, D. Y. Alalawi, S. Awad, A. Karamarkovic, J. A. Košir, M. Flint, A. Victor, S. S. Verhage, F. Gool, T. Mabogoane, B. Phakathi, V. Pillay, R. Jayakrishnan, Y. Manickchund, O. Jolayemi, H. Stark, C. Molewa, H. Wain, D. Montwedi, G. De Wee, C. Dempers, H. Aguado López, M. D. M. Martí‐Ejarque, A. Torroella, O. Martin Sole, A. Landaluce‐Olavarria, V. Alonso, Á. Fernández Camuñas, M. Estaire Gómez, A. G. Barranquero, L. Marquez, P. Serrano Méndez, A. Vilar, J. Guevara, A. M. Minaya Bravo, J. L. Rodicio Miravalles, R. Díaz Pedrero, L. Tallon‐Aguilar, A. Curado Soriano, Z. Balciscueta, D. Moro‐Valdezate, B. De Andrés‐Asenjo, A. Vazquez Melero, J. Escartin, C. Gracia‐Roche, S. Srishankar, D. Wickramasinghe, U. Jayarajah, S. Gobishangar, W. Wijenayake, I. Abdalla, S. Ibrahim Tour Harakan, M. M. Yassin, Z. Aljalabi, I. Adel, I. M. G. Ahmed, M. Hajalamin, E. E. Abuobaida Banaga 11 Hag El Tayeb, H. A. Fadlalmola, E. Alkhalifa, H. Zaigham, M. Nikberg, P. Probst, E. Gialamas, J. Gass, A. Tampakis, G. Peros, M. A. Schneider, M. A. Farho, A. A. Kayali, M. Aloulou, A. Ghazal, B. Alsaid, M. Klib, H. Dalati, S. Jomaa, Y. Alhammoud, S. Abbas, G. Hneino, I. Ali, G. Bashour, A. Hammed, S. Techapongsatorn, F. Alassani, A. Sebai, G. C. Bulbuloglu, M. A. Koç, M. Y. Uzunoglu, B. Yigit, A. N. Sanli, G. K. Kurtoglu, E. Tuzuner, Y. Altinel, Ö. P. Zanbak Mutlu, R. E. Sönmez, S. Bektas, E. Erginöz, A. Özcan, Y. Tosun, İ. H. Özata, T. K. Uprak, E. Unal, N. Kiziltoprak, M. Ergenç, M. T. Demirpolat, B. Citgez, H. Ulman, Y. K. Şen, E. Colak, N. Kavak, E. Kamer, I. Mubezi, H. Lule, S. Stonelake, C. S. Ong, P. Patel, S. Dindyal, F. Georgiades, J. Abbasy, M. Kaur, B. Martin, M. Chauhan, S. Ahmed, M. Tutton, N. Chidumije, W. Al‐Khyatt, A. Sukumar, H. Kamal, A. Nada, A. Chaudhary, M. Bogdan, M. Peter, J. Walshaw, M. Ewedah, L. Rampersad, A. Peckham‐Cooper, N. S. Blencowe, R. Lunevicius, P. Panahi, E. Baili, K. Theodoropoulou, P. Kapsampelis, M. M. H. Mohammed, C. Parmar, M. M. H. Mohammed, C. Smart, P. Wilson, F. Gareb, G. Sundaram Venkatesan, C. Hidalgo Salinas, S. Tingle, N. Marzouqa, T. Theivendrampillai, M. Zahed Abdalla, A. Rahman, M. Abdelkarim, O. Whitehurst, E. Tokidis, S. Bandyopadhyay, B. Al‐Sarireh, A. Salam, M. Sulciner, G. Chang, A. Alecci, H. E. Rice, D. Ridder, K. McKenzie, A. Choudhry, M. Y. Abdualqader, B. Alshaikh, I. Dajti, K. Bensmain, Z. R. Benamrouche, I. E. Boumakhlouf, I. E. Boudis, H. Abdoun, M. Benamrouche, M. Saidani, Z. Djama, A. Chied, H. A. Mimouni, A. K. Awad, B. Radja, B. Abdennour, M. N. Bouhafs, M. E. A. Meghaizerou, P. Carmignani, J. Mondino, R. Figueroa, J. Morales, F. R. Pascual, M. A. Bequis, F. Suldrup, C. Korzin, J. Napoli, N. Feijoo, F. Mahnic, M. E. Duran, L. Chantada, C. Brandi, J. F. Viñas, F. Lucero, C. Samojeden, L. J. Caram, S. Bertone, F. Corvatta, L. Garciandia, M. Rius, S. Matthiess, J. Paredes, M. C. Kalaydjian, A. Veira, M. A. Fernández Zurita, J. I. Valenzuela, S. Gomez, G. R. Viscido, M. A. Doniquian, R. Badra, J. S. García, C. I. Ferrero, M. Garcia, L. Granero, M. Pagani, M. Gosselink, J. Ringers, M. Lie, B. Mao, C. Stennard, E. M. A. Murphy, J. E. Do, M. Harris, B. Fosh, M. Watson, J. Petric, M. Maclean, X. Y. Po, M. Pham, D. Patterson, V. Gunasaegaram, E. Hopping, P. Holt, J. A. Duffield, E. Schmidt, R. Colbran, V. Liu, E. Tan, J. S. T. Tefay, R. Shen, S. Bowman, D. Mitchell, M. Kelly, A. Edmundson, H. Iswariah, R. Franz, M. Chandrasegaram, P. Yuide, S. S. Hlaing, S. Abeykoon, D. Kaushal, C. Leung, S. Davis, N. F. Franco, T. Rawther, R. McClen, W. Petrushnko, E. Roussos, K. Das, S. Stevens, F. Alnimri, S. McClintock, J. Maritz, S. Hariharan, S. Laura, B. Wang, V. Ng, J. Linker, A. Li, I. Dong, R. Bhatia, S. Cai, W. K. H. Lai, A. C. Dawson, S. Y. D. Chia, M. Binks, N. Tran, S. H. M. Ng, D. Shen, E. W. Y. Lun, E. Reid, J. Cui, M. Roussos, M. Issa, M. Anandan, P. Devlin, U. Naidoo, B. Balaravi Pillai, D. Abeysirigunawardana, T. Parker, T. Valizadeh Elizeh, D. Liu, S. Ng, J. Jones, O. Ladlow, J. V. Maida, D. Proud, A. Vu, N. Shulman, L. Bromley, V. Muralidharan, K. Hall, C. Cheong, C. Jamieson‐Grigg, A. Hilder, T. Manickam, L. Barnard, K. Jaffry, A. Gray, A. Lim, R. Kattini, M. Bickford, S. Kenworthy, A. Crowe, J. Zhu, M. Pacilli, A. Comella, K. Taghavi, R. Nataraja, S. J. A. Robinson, D. Lowen, A. Khan, S. Samadi, S. Tan, A. L. Surkitt, S. Condron, E. Haege, E. Francis, A. Boynes, S. Gill, B. D'Souza, H. Xiao, E. Fraser, J. Wong, S. Fennelly, K. Mori, M. Muir, Y. Huang, R. Pajtak, J. You, C. Banal, T. Abelman, N. Chen, L. Chong, H. Jalilehvand, W. Santucci, B. McKay, I. Murshed, D. Makary, L. Green, M. Wichmann, A. Lim, M. Kang, E. A. Dontoh, P. Walker, A. Fani, S. Sundararajan, E. Downes, A. Davis, M. Bajwa, R. Geow, K. K. Sim, S. Smith, L. Peters, S. Zhang, A. C. K. Cheung, I. Caitens, M. Ishak, E. Zhang, K. K. A. Yu, L. Beukes, L. Kang, F. Amico, M. Bowles, E. Downing, B. Williams, G. Cox, V. Bakshi, N. Ensor, J. Ng, C. T. Petcu, J. Hwang, T. J. Hugh, S. Quoy, C. Knee, D. Siriwardena, C. Tung, G. Smith, O. Camilleri, J. Vu, J. Hong, C. Cornwell, E. Chan, C. Killoran, J. Mackenzie, D. Fry, V. Mahendravarman, A. Shanmugalingam, C. Li, L. Allan, P. Shivashankar, H. Pleass, V. Lin, R. McGee, B. Bereket Araya, C. W. Un, Y. De Silva, D. Maan, J. Wang, M. Kwon, R. Kibuuka, J. Chew, J. Siu, J. Barklimore, N. Klammer, R. Schmidt‐Branden, P. Tschann, P. Horvath, N. Koter, G. Moitzi, E. Wallner, C. Allmer, F. Aigner, J. Kahn, A. Belarmino, R. Sucher, V. Wolfschluckner, G. Singer, R. Függer, M. Biebl, A. Punzengruber, H. Fehrer, A. Binder, E. Haiden, P. Riedl, M. Enßlin, K. Nahar, T. Akter, A. Oosterkamp, G. Gbessi, J. Avakoudjo, M. Fiogbe, P. Assouto, S. P. Chigblo, H. Aouagbe Behanzin, M. Seto, G. Mevognon, M. Agbadebo, A. Hada, S. F. A. Houndji, E. Bara, A. B. Yevide, Z. F. Tamou, E. Hatangimana, B. Cakpo, R. Soglonou, T. K. Hessou, S. R. Tobome, M. Zounon, A. M. Hodonou, C. Bokossa, F. Hounde, R. Alinde, F. Dossou, R. Goudou, A. C. S. Toi, G. Natchagande, M. Stjepanovic, O. Čančar, M. Pejović, J. Miskovic, M. Boras, M. Kajic, V. Dragisic, Z. Brekalo, I. Mikulic, N. Soldo, M. Bevanda, M. Faletar, M. Salibašić, E. Hodžić, E. Halilović, M. Kruščica, E. Bičakčić, A. Cerovac, H. Škiljo, E. Hodžić, O. Bedak, M. Kalabić, E. Begunić, A. Huremovic, E. Alić, R. A. Tenfen Carneiro, D. Georgiev, I. Fidoshev, V. Neykov, E. Daleva, I. Ilieva, M. Karamanliev, D. Dimitrov, A. Shanker, P. Vladova, M. D. Shoshkova, A. Mehta, M. Abdullahi, V. Ratheesh, V. Kamalathevan, C. Wiesner, S. Shittu, M. Galasyuk, S. Shanker, M. Imirski, A. Soumpasis, E. Hadzhieva, D. Chakarov, T. Yotsov, P. Kamenova, A. Vricheva, I. Yotsov, E. Hristova, K. Spassov, A. Sanou, M. Windsouri, R. Doamba, I. W. Bahikoro, A. S. T. Sanon, N. Ildephonse, N. Steve, Y. Fulgence, G. D. Nibogora, C. Nimbona, B. Paul, G. Kazobinka, C. Rukundo, N. Renovat, E. Ndizeye, M. Dauphin, F. F. Irakiza, L. Niyidukunda, G. Nkunguzi, N. Oscar, N. Theophile, B. Révérien, S. Oum, S. Eam, N. S. Bibila, N. N. Cabrel, J. Dongmo, R. Spence, G. Berger, D. Hannedige, C. Hoogerboord, A. Abidali, M. Mozel, L. Monteiro, R. Lertnamvongwan, D. Konkin, R. Kaur, M. Mozel, R. Lertnamvongwan, L. Monteiro, S. MacKenzie, D. Konkin, M. M. Modolo, E. Sepúlveda, Á. Molero, M. Perez, L. V. Torres Bavestrello, Ó. A. Soublett Rivas, C. Carrillo Sarango, L. Paredes, P. Sornoza, A. Pinto, C. González, J. Wang, L. García‐Zambrano, P. A. Cabrera Rivera, N. Paez, S. V. Agudelo Mendoza, M. S. Mosquera Paz, A. Kadamani Abiyomaa, C. F. Roman Ortega, F. Casas, B. Guerra, J. D. Molina Marin, C. Maya, C. Vasquez Maya, B. Dieck, F. Zapata, V. A. Ruiz López, M. A. Ñañez, D. C. Cardona Gomez, A. Rojas, D. C. Patiño García, L. I. Bolaños, C. Pastás, D. A. Pérez Muñoz, J. Mihanovic, I. Bacic, D. Vukosav, V. Žufić, O. Jurić, E. Dijan, N. Jović, I. Ćoza, I. Rakvin, Z. Katusic, T. Soric, I. Vidić, D. Rukavina, I. Separovic, R. Radojković, J. Mavrek, M. Theodoridou, A. Pilavas, R. Moukarzel, R. Andreou, N. Gouvas, K. Lambri, S. Charitonos, I. C. Mylona, G. Kokkinos, N. Dimitriou, M. M. El Ghoul Miliotou, V. Ioannou, N. A. Ververidis, N. Kalampokis, A. Yiallourou, R. Sokratous, D. Tsiardas, P. Evangelou, D. Evripidou, C. Thrasyvoulou, T. Reichelt, P. Hudáč, K. Akter, L. Moolla, F. Rudisch, A. Ibrahim Hassan, O. Ahmad, E. El Shennawy, M. Shalaby, M. Khaled, A. Akiba, F. Philips, E. Bankart, R. Elshennawy, A. Al Kaddah, H. Al Atassi, S. Ashry, N. Salgadoe, L. Majerčák, P. Levíček, A. Lukáč, L. Pánči, J. Roman, L. Tulinsky, I. Mrazkova, P. Ostruszka, A. Varga, L. Martinek, H. Novák, J. Woleský, P. Francúz, R. Rivas, B. Calcaño, J. Michel, Y. Perez, R. Ubiñas, P. Garcia‐Dubus, S. Batista, S. Strachan, Y. Tanas, Y. Kerolous, Y. El Okazy, M. Mokhtar, M. Lotfy, M. A. L. Sayed, H. Altabbaa, A. G. M. M. Abouelnagah, O. Al Shaqran, D. M. Awad, A. Sabry, G. Nagy, E. Amer, M. Khalil, A. El Shamarka, B. Sharaf Eldin, A. A. A. Aboshosha, A. Farrag, H. Sherif Farouk Ahmed Hassan, Y. Badr, Y. Orabi, M. Kamal Matter, A. Alrifaee, M. Elnour, M. Zahran, A. Aladl, M. Bahnacy, Y. Seada, M. Kotb, A. Ragab, Y. Farag, L. Khalifa, M. Elmiesiry, D. Abdalaziz, I. Maharem, O. AbouHiekal, O. Hany, S. Hanna, Y. Dean, A. Faisal, M. Mostafa, I. Ali, T. Sabra, H. Ibrahim, A. K. Ali, M. Osman, A. Eltayeb, A. Morad, M. O. Herdan, A. Abdelshafi, M. M. Nathan, M. Shalkamy, M. Hamada Takrouney, R. Sayad, F. A. Monib, A. A. Elhars, M. M. Saad, A. Rashad Temerik, A. M. Abbas, O. Mohamed Mokbel, E. AbdElBaset, A. Barakat, Z. Bady, S. Arafa, Z. Osama, A. Elzanaty, S. Salama, M. E. M. Madany, A. Khaity, R. Adel Diab, A. Ghazal, A. Ehab, A. Abd Elsattar, A. El‐bastwesy, A. Eisa, M. Elesseily, R. Radwan, Y. Asar, D. Waleed, S. Tawfik, A. F. Nixon Fulli, M. J. I. Albert, A. Autiak Ayii Chol, A. R. AbdelHalim, B. Azhar, H. Al‐derume, M. Alqadasi, N. Alasbahi, H. Abozied, Y. Ashour, Y. Mohamed, M. Abdelmaboud, H. Abdelazim, A. E. S. El kady, M. Omar, A. Haty, M. Abd Al‐Fattah, I. Tagreda, I. M. Kereet, A. G. Montaser, M. Faisal, M. Masoud, M. ElSayed Metwally, A. S. M. Abdelrahman, S. Mansour, A. Nabil, M. M. A. Marei, A. Elmosalamy, L. ElGebaly, A. M. Allam, T. Awad, H. Taher, K. Fayed, M. Abdelfattah, D. H. Khattab, N. Ali, A. Saleh, K. Nassim, N. K. Aly, I. Abo Elhagag, M. Doss, M. Elzayat, Y. Samer Morsy, M. ElFiky, M. Erfan, M. Zaazou, M. Reda, M. Kouta, M. Mohamad Amin, I. Guirguis, H. Amir, S. Aboseif, M. Abdelhafez, O. Agha, A. Khairy, A. Dawoud, M. Hamoud Almahly, S. Mostafa Yassin, A. El‐Sherbiney, A. Adel, H. Foda, D. Ahmed, A. S. Elkhodary, A. A. Mansour, A. Elghrieb, M. Natey, A. Elshazli Mahmoud, A. Khalleefah, H. Elfeki, M. Shalaby, M. Sadek, M. Abdelmaksoud, M. Mostafa, M. Waseem, A. Adel, A. Azam, A. Sakr, A. Sanad, عبدالفتاح م, H. Foad, S. Elnoamany, S. Selim, D. S. Alrokh, A. Hassanin, M. Alansary, A. Ragheb, M. Fahmy, M. Mehanny, A. G. E. Aboelnasr, K. M. G. Mohammed, M. Eissa, S. Allam, M. Kamar, A. Asla, F. Terefe, W. A. Zerefa, E. Gallo, A. Yingess, T. Kebede, M. Mesfin, G. Alemayehu, S. M. Djote, T. Girma, D. A. Muhie, E. Yeshialem, G. Seyfu, F. Tsige, A. Yeshitila, N. Solomon, M. Worku, S. Lakew, Y. Melkamu, S. T. Workineh, M. Beletachew, B. Mengesha, G. Getachew, I. Tesfahun, M. Teressa, B. G. Chiman, A. B. Aregawi, N. S. Bayleyegn, Y. Y. Metaferia, T. G. Moges, D. Mengiste, A. Teshome Sahilemariam, B. Sime, T. Jemal, E. Volpin, H. Braham, C. Lionel, R. Arena, Y. Malki, M. Bertrand, A. Castaldi, L. Theuil, A. L'Hostis, M. Prudhomme, P. Riva, A. Lapergola, D. Mutter, S. Perretta, P. C. Nze Obiang, Z. Demetrashvili, G. Devidze, G. Pisarevi, L. Petashvili, E. Ekaladze, N. Lekiashvili, I. Pipia, A. Tvaladze, G. Kenchadze, K. Khutsishvili, L. D. Lee, J. Binder, A. Denz, C. Krautz, M. Brunner, M. Maak, G. F. Weber, A. Stollberg, D. Hackner, S. Engel, F. Krämer, R. Grützmann, M. Schüler, J. Kleeff, R. Rüdrich, A. Kirschniak, J. Rolinger, S. Göller, L. Van den Hil, J. Miller, H. Pehlivan, M. Kießler, N. Hüser, D. Schippers, M. Berlet, M. Steffani, M. Weber, N. Börner, M. Albertsmeier, H. Arbogast, M. Mattis, S. Jarmusch, P. Zimmermann, U. Wirth, F. Anzinger, F. G. Bader, M. Sohn, M. L. Koschke, M. Busch, N. Hielscher, A. Brosin, J. Lindert, M. Philipp, M. Gumsheimer, F. Wiese, L. Sahan, G. A. Stavrou, J. De Deken, M. Jabal, M. W. Löffler, A. Königsrainer, M. Quante, C. Yurttas, R. Armah, N. A. Christian, D. Daary, S. Akuffo, A. Twumasi, A. D. Andani, J. Oppong, E. Agbowada, J. Daleku, J. Ampadu, W. Afedo, Z. Robertson, A. Obbeng, D. N. Lee, D. Ofosuhene, G. D. Brown, F. Osman, F. J. Eshun, C. Banka, I. Amankwaa, E. Ametefe, G. Owusu, J. Nyamekye ‐ Baidoo, O. Okrah, G. Birikorang, P. Kumassah, J. Dei‐Asamoa, J. Annan, C. Akli‐Nartey, D. Alifoe, S. Tsatsu, C. Ansah Larbi, U. Una, K. Yalley, A. Bediako Bowan, K. B. Oduro‐Boateng, S. Anim, H. Adjei, S. Dognia, A. Oppong, C. Markin, J. L. Ahale, N. K. A. Obuobi, S. Agana, E. B. Akakpo, F. Galley, F. E. Gyamfi, S. Segnitome, S. Agordjor, D. Adjei, D. Kyeremeh, Y. Sarpong, F. Opoku Twene, C. K. Ntow‐Boahen, S. A. A. Atupra, V. Siepaal, I. N. Bakaweri, A. J. Tabiim, R. Agyei Boakye, A. Asare Twumasi, R. Akankoatuesi Apatewen, U. Kanyan Kassim, B. Owusu Ansah, F. Amoako, V. Kudoh, K. Boakye‐Acheampong, B. Boakye, R. Kpangkpari, M. T. Morna, G. A. Rahman, E. O. Ofori, L. Adagrah Aniakwo, M. Amoako‐Boateng, D. T. Enti, S. Debrah, M. Nortey, P. Koggoh, P. Mensah, M. M. Agyapong, T. Agyen, V. Etwire, Y. Adofo‐Asamoah, S. Yussif, M. Yigah, B. Maanikuu, F. Kuubetersob, D. Powell, A. Gbeadese, F. Tierenye, E. A. Nachelleh, D. Y. D. Agbley, N. Jiagge, R. Akpaka, D. Labadah, N. Naabo, R. Guzmán Lambert, F. J. Eshun, P. Ntem, E. Setsoafia, N. Affram, B. Y. Hernandez Cervantes, D. B. Osei, K. Ewool, F. Nyarko, M. A. Ali, M. A. Oyortey, I. Hagbevor, M. E. Ashong, J. N. Anyorigiya, J. Yorke, A. Lovi, E. O. Osei, P. A. Boateng, R. Oppong‐Amoah, K. Agbedinu, C. Dally, S. G. Brenu, F. Galley, F. M. Agbemafoh, I. Kyei, C. Aboah, A. Y. Appiah‐Kubi, B. Nimako, M. Aikins, M. Adinku, A. Opoku‐Agyapong, J. Adjei, R. Sagoe, A. Gyedu, P. K. Boateng, S. Mensah, E. Frimpong‐manso, P. Taah‐Amoako, S. Tabiri, F. Owusu, P. Yeboah Owusu, E. M. T. Yenli, A. S. Seidu, M. Dason, M. Amadu, G. A. Adoro, M. Kyereh, I. Osman, J. Quansah, C. Doku, A. Darkwa Boateng, A. M. Muntaka, M. A. Dokurugu, R. Nesco, M. Yahaya, E. D. F. Konlan, A. Issaka, R. A. Ramirez Calas, M. Sheriff, M. Dery, V. Dassah, B. K. Seshie, F. Caiquo, M. Dum, L. Ackam, K. Yalley, F. D. A. Agbodo, A. Baiden Amissah, D. Ashitey, L. D. Bray, J. Ofori, M. Ishak, G. Ansong, I. Gogoulis, K. Bekiaridou, A. Mitsala, S. Botaitis, C. Tsalikidis, M. Asimakidou, C. Nikolaou, E. Efremidou, C. Limas, P. Chloropoulou, M. Aggelidou, M. Pitiakoudis, P. Kostoglou, G. Pappas Gogos, M. Karanikas, E. Kapasakis, M. Karakeke, A. Skarpas, C. Floros, K. Athanassiou, E. Karakeke, N. Tasis, A. Sarafi, A. Plastiras, G. Kavalieratos, T. Tsirlis, L. Chardalias, N. Memos, V. Themelidi, I. Papaconstantinou, T. Theodosopoulos, D. Politis, K. Iliakopoulos, K. Bramis, P. Antonakis, A. Skreka, D. Kotsaris, N. Dafnios, A. Vezakis, I. Contis, T. Petropoulou, K. C. Kordeni, T. Kozonis, G. Fragulidis, D. Massaras, E. Apostolopoulos, I. Karatsolis, A. Mourtzouni, K. Avgerinos, D. Kelgiorgi, K. Polychronopoulos, G. Kostoulas, A. Tsechpenakis, A. Saridaki, A. Ioannidis, C. Chouliaras, I. Tierris, M. K. Konstantinidis, D. K. Manatakis, D. Balalis, N. Stamos, N. Tasis, V. Kalles, T. Sidiropoulos, M. Papadoliopoulou, N. Arkadopoulos, D. Sampanis, P. Vassiliu, E. Dylja, A. I. Nikolaou, I. Margaris, P. Kokoropoulos, V. Tsaousis, S. Christodoulou, E. Poulios, A. Chamzin, S. Kapiris, E. Mavrodimitraki, M. Sotiropoulou, N. Dimitrokallis, N. Papadogianni, V. Vougas, M. Papamichail, K. Rekouna, K. Pavlopoulos, N. Roukounakis, A. Thanasa, P. Trakosari, M. Christou, E. Saitoglou, C. Nastos, D. Dellaportas, P. Lykoudis, N. Garmpis, G. Kouraklis, P. Christodoulou, G. Kapogiannatos, A. Nikitaras, S. M. Tsoti, J. Katogiritis, E. C. Tampaki, C. Papazacharias, O. Bellou, N. Machairas, P. Dorovinis, C. Doudakmanis, D. Schizas, A. Syllaios, M. D. Keramida, P. Stamopoulos, S. Davakis, M. Despotidis, A. Panagakis, S. Kykalos, F. Stavratis, M. Vailas, L. Karydakis, N. Kydonakis, A. Loizou, K. S. Giannakopoulos, P. Sakarellos, A. Kozadinos, I. Katsaros, C. Damaskos, E. Antoniou, M. Mavri, I. Psilopatis, S. Vernadakis, I. Bokos, P. Paraskeva, D. Vardakostas, I. Gomatos, A. Barlas, A. Smyrnis, D. Prevezanos, N. N. Mathioudakis, I. Kozadinos, A. Kozadinos, P. Kanavidis, N. Garmpis, E. Spartalis, M. Spartalis, C. Stefanou, S. Gkogkos, L. Fountoulis, I. Sougkas, M. Billis, A. Kontokostopoulos, A. Balta, T. Padioti, M. A. Sotiriou, A. Theochari, N. Tsakiridis, I. Tsakiridis, E. Synekidou, E. Athanasopoulou, C. Ntagkas, E. Samara, A. Katsiou, D. Panagopoulos, A. Panagopoulos, L. Katsiaras, C. Kolla, L. Mansour, G. Koukoulis, S. Zourntou, D. Papageorgouli, K. Bouliaris, L. I. Fountarlis, M. Bei, A. A. Kalidis, X. Vagena, A. Gkouniaroudi, A. Migdanis, A. Bakalis, E. Gavriil, K. Koumarelas, M. N. Kouliou, K. Bouchagier, F. Mulita, G. Verras, G. Skroubis, I. Maroulis, V. Mousafeiris, A. Panagidis, A. Papadopoulos, P. Grivas, F. Spanos, A. Kalogeropoulou, G. Zeringa, C. Kourouniotis, G. Rados, E. Barkolias, K. Zakkas, I. Demiris, V. Nikolaou, K. Tata, G. Karakaidos, E. Kontis, E. Papamattheou, A. Efstathiou, E. Efstathiou, N. Kopanakis, K. Ntatsis, I. Katsaros, P. Manikis, V. Tselepidis, M. Kyriazi, I. Siannis, N. Kouzakos, V. Georgilaki, N. Vlachakos, S. Vederaki, A. Tsiaka, A. Zarafidou, N. Zampitis, S. Tsatsos, F. Stefou, F. Kyramargios, M. Merrakos, G. Bekakos, A. Marinis, O. Ioannidis, E. Anestiadou, K. Zapsalis, S. Simeonidis, S. Bitsianis, M. Drogouti, A. Gkoutoula, A. Sarakatsanos, E. Efthymiou, I. Chatzis, I. Spyridakis, C. Kaselas, M. Tsopozidi, M. Florou, C. Demiri, V. Papadopoulos, D. Giakoustidis, A. Giakoustidis, D. Alexandrou, P. Chatzikomnitsa, S. C. Liapis, K. Perivoliotis, C. Chatzinikolaou, N. Tsantikos, Z. R. Karampotaki, D. Lytras, M. Aguilera‐Arevalo, M. Rodríguez‐Ordoñez, D. A. Sosa Méndez, M. Sebastián‐Mendoza, T. A. Salazar‐Lorenzana, R. Herrera, D. E. Reyes Rodríguez, P. Vásquez, S. Morales, J. Gomez, C. García‐Salas, J. R. Asturias Luna, J. Tabora‐Zepeda, S. Vasquez, J. R. Hernández, J. O. M. Herrera Batres, D. Muñoz, E. Ayala, O. Coyoy‐Gaitán, J. B. Pellecer Cano, D. A. Palma Portillo, M. Blanco, D. Herrera, S. A. Villeda, O. Lima Azurdia, S. Jp, M. Bhat, A. Raheja, I. Shariff, H. Anand, B. R. Budihal, S. Kashyap, B. Arya, N. Krishnappa, M. Manangi, P. Anandan, S. Shivashankar chikkanayakanahalli, S. Kumar Venkatappa, T. S. Mishra, P. Kumar, M. Gureh, M. K. Sethi, A. A. Asharaf, Y. Sakaray, S. Irrinki, S. Subbiah Nagaraj, S. Khare, C. Tandup, A. B. Muthunayagam, A. Prasath, P. Arunachalam, P. John, S. Raul, R. Vakil, R. Sinha, E. Dvivedi, A. Thomas, S. Joseph, A. Sharma, B. Khan, D. Chatterjee, R. Gupta, A. Khanduri, S. Singh, D. H. Tyagi, U. Daspal, N. Rawal, R. Varshney, M. Luthra, R. Handa, S. Basu, P. Chadha, R. Sethi, T. Longkumer, K. K. Mishra, S. Sundaramurthy, S. Kumar, D. Phom, J. Kaippally, D. Ommi, L. Imchen, T. Alinger, A. Chhabra, A. Kumar, B. Kharga, M. Sarda, K. Bhutia, N. Sharma, M. S. Rodha, N. Banerjee, A. Baksi, S. Kaur, R. Chaudhary, M. Lodha, S. P. Meena, M. Badkur, I. Singh, A. Sinha, K. J. Rathod, R. Saxena, J. Tk, A. Vig, M. Pathak, A. Sukhdev Jadhav, S. Nayak, T. Motiwala, K. Shreyas, S. R. Pathan, J. Rathod, C. Agarwal, K. Sharma, S. Pandya, A. Anand, A. Kumar, H. S. Pahwa, A. A. Sonkar, M. K. Agrawal, A. K. Pal, D. Jain, P. D. Haque, V. Michael, W. Bhatti, J. Dhiman, D. R. S. Thind, A. Bhatt, P. Gupta, A. Luther, S. Khurana, R. R. Ranadive, A. Suroy, H. Kaur, P. Shukla, N. K. Chaudhry, D. A. Hajela, P. Patel, P. K. Arya, D. D. Dhawan, D. R. Tripathi, K. K. Luthra, A. Kumar, D. H. Gupta, A. Mathew, C. Pun, P. Dummala, M. Gurung, P. Alexander, N. Aruldas, P. S. Prabhu, S. Payyanur Thotan, B. Sv, R. D. Sharma, R. Redkar, R. Nathani, S. Karmarkar, A. Sharma, S. Singh, S. Achugatla, A. Bangar, D. D. Kulkarni, K. Raghuwanshi, D. N. Nikam, S. N. Mahendra, B. Sarang, D. Belekar, K. Gaikwad, A. John, P. A. Thomas, L. Pramod, D. Gavit, D. Singh, N. Kansakar, N. Gupta, N. Kapur, N. Narain, S. Kulkarni, T. Rashid, M. Husain, F. Tauheed, S. V. Manzoor, S. Ohri, L. Bains, P. Lal, S. Neogi, A. Mishra, S. Ahuja, T. Iahmo, M. George, M. Singh, P. Waghchoure, A. Choudhrie, A. Kumar, M. Aggarwal, V. Kanna, S. Vembar, T. P. Singh, D. S. Walia, V. Singh, G. Kaur, A. Jindal, P. Dhamija, M. Kumar, A. Sinha, A. K. Jha, M. Sharma, A. Bhadani, R. Abhinaya, U. Kumbhar, A. Jain, S. Chilaka, V. S. Jha, A. Jayapalan, Y. Vashishth, G. Jalal, V. V. Nair, C. Raphael, H. K. Prabhakar, Z. Khan, D. Dugar, D. Mohanty, T. D. B. Tridip, D. R. Ramchandani, S. Basu, N. Kumar, A. G. Goswami, H. Panga, D. Mallik, A. Gupta, D. Rajput, R. Anjum T Siddeek, P. Manjunath, S. Edem, F. Huda, S. K. Singh, S. Karuppusamy Krishnasamy, S. Katragadda, K. M. R. Reddy, I. Ahmed, E. Yhoshu, L. Manoj Joshua, A. Das, P. Kothari, K. Singh, S. S. Malhi, R. Kaur, H. K. Cheema, M. Singh, N. Saini, M. Gupta, A. Bhatti, A. Gupta, P. Kaur, N. Pahuja, H. Kaur, S. Chopra, B. Sehgal, G. Singh, D. S. Kshirsagar, M. Kaple, G. Saxena, S. Dhole, A. Bhargava, C. Mahakalkar, S. Deshpande, R. Wani, R. A. Dar, A. A. Malik, N. Bhat, Z. A. Shah, G. A. Bhat, S. Kondpan, A. Kutma, J. A. Kalyanapu, A. Sundaram, R. Krishna raj, G. George, M. Chisthi, H. Jafarkhan, U. Govindan, C. Narayan, G. Pillai, V. V. Kollengode, D. Jabbar, T. Tony, A. Nair, A. Kavalakat, A. Moncy, A. Johnson, A. Joseph, A. Appukuttan, A. N. Oommen, S. Francis, N. Srinivas, M. Narayanan, T. Devabalan Koil, M. R. Jesudason, Y. Myla, A. S. P. Dhinakar, A. Tirkey, B. Roopavathana, R. Mittal, S. Surendran, N. Paul Ambrose, D. Joshiba, P. Trinity, R. Raghunath, N. P. Paul Sigamony, P. Y. George, R. Philip Sridhar, S. Chase, S. J. Arthur, N. Yousefzadeh Kandevani, M. Pourfridoni, H. Askarpour, H. Mohammadi sardoo, A. A. Kheirkhah Vakilabad, M. Ali‐Hassanzadeh, R. Raheem Attallah Al_obaidy, Z. Alkhuzaie, S. Salim, F. R. H. Hassooni, Y. Zwain, H. M. A. Oneizah, S. Razaq, M. Razaq, H. H. Z. Zaini, E. Colton, K. E. Oderoha, A. Gill, S. Ramjit, F. Ghazali, K. Griffin, R. Ahmed, C. Weadick, S. Nair, V. Sharma, C. Donohoe, R. Tummon, B. Maguire, S. Wrenn, R. Habib, P. Owens, A. Shweiki, G. Szydlo Shein, G. Almogy, Y. Mintz, A. Pikarsky, R. Elazary, O. Cohen‐Arazi, J. A. Demma, H. Mahajna, B. Helou, Y. Fishman, G. Marom, M. De Prizio, K. Kröning, R. Sulce, L. M. Fatucchi, F. Tofani, V. Mariottini, R. Malatesti, M. Scricciolo, G. A. Pellicano', A. D'Ignazio, A. Mazzoni, A. Biancafarina, M. Angelini, V. Borgogni, L. Rossi, G. Munzi, G. Tarantino, M. Castrovillari, A. Serao, J. R. Casella Mariolo, G. Del Corpo, A. Natili, A. Iodice, S. Gargiulo, B. Esposito, M. Pannullo, L. Bracciano, E. Marra, A. Alberico, A. Gori, S. Cardelli, G. Dajti, C. Larotonda, I. S. Russo, J. Andreuccetti, S. Molfino, D. Alberti, G. Pignata, G. Emiliani, G. Boroni, M. Ruffoli, M. Manfredini, L. Sequi, G. Zanni, E. Locci, M. Podda, V. Murzi, C. Piras, A. Carta, A. Pisanu, T. Pilia, P. Marongiu, A. Saba, M. Pisano, F. Campus, E. Gessa, E. Silanos, A. Lai, F. Frongia, F. Corronca, S. Montisci, F. Cappellacci, C. Soddu, G. L. Canu, F. Medas, P. G. Calò, S. Puddu, M. Biancu, M. Abbas, F. Casti, B. Demurtas, A. Deserra, F. D'Agostino, C. Margiani, L. Laface, M. Casati, M. Mariani, S. Guarriello, A. Balconi, B. Scotto, N. Laquatra, R. Ruccella, G. De Angeli, G. Gravante, A. Chiappini, R. Lopatriello, G. Mammolo, C. Distefano, G. Riccioli, R. Granata, M. Veroux, D. C. Centonze, S. Costa, R. Gioco, D. Zerbo, A. Licciardello, L. Stella, L. Rende, M. Osso, D. Paglione, F. Pata, D. Sasia, G. Giraudo, D. Ribero, S. Alberti, V. Schirinzi, P. Belotti, L. Taglietti, V. Giordano, A. Pesce, C. V. Feo, M. C. Pignanelli, S. Severi, F. Cammelli, F. Natali, M. Scheiterle, G. Maltinti, L. Fortuna, F. Coratti, A. Manetti, J. Martellucci, E. Monati, L. Gabellini, R. Fratarcangeli, A. Damigella, E. Adinolfi, A. Anastasi, M. Montagna, A. Giuliani, G. Procaccini, N. Tartaglia, F. Vovola, D. Merlicco, S. Schirone, S. T. Massa, G. Pavone, M. Pacilli, F. Maffei, A. Gerundo, D. Di Pietrantonio, S. Quartarone, L. Solaini, G. Ercolani, L. Ragazzini, V. Zucchini, M. Cammelli, D. Scotto Di Carlo, F. A. N. Marin, A. Azzinnaro, A. Razzore, A. Petrungaro, E. Mina, B. Sperotto, G. Carganico, D. Pertile, D. Soriero, R. Diaz, A. Luzzi, S. Carrabetta, C. Meola, D. Caruso, F. Floris, P. Grondona, E. Romairone, S. Marzorati, F. Ré, C. Righetti, A. Viacava, L. Epis, R. Sampietro, D. Gobatti, C. Zandonella, V. P. Dinuzzi, U. Rivolta, S. Luciano, G. M. F. Marini, L. Scaravilli, R. Magarini, G. Saletta, A. C. Sironi, G. Grava, M. Mercurio, D. Zulian, A. Izzo, M. Gritti, S. Giudici, E. Desiato, M. Molteni, L. Ottaviani, G. Grande, E. Mazzotta, S. Grimaldi, F. Brucchi, F. Ferraina, S. Lauricella, E. Di Marco, L. C. Nespoli, G. De Carlo, E. A. Baccalini, D. Palmisano, A. Scacchi, N. Tamini, L. Ripamonti, M. Rennis, C. Vitiello, A. Davolio, L. Degrate, P. Masseria, V. Brocco, P. Chiacchio, M. Ceresoli, E. Signaroli, A. Finocchio, C. Fumagalli, M. Binda, M. Milone, M. Manigrasso, S. Vertaldi, A. D'Amore, G. D. De Palma, A. Marello, L. Fedele, C. Sorrentino, D. Pignatelli, G. Luglio, F. P. Tropeano, M. Cricrì, A. Miele, G. Aprea, G. Palomba, M. Capuano, R. Basile, G. Sorrentino, D. Rega, A. Ottaiano, V. Granata, A. Belli, F. Izzo, G. Pellino, D. Massaro, V. Mosca, F. Selvaggi, G. Bellio, L. Rubin, N. De Santis, N. Schiavon, A. Zerbinati, S. Corso, C. Cecconi, P. Venturelli, G. Cocorullo, G. Carollo, R. Tutino, A. Bonelli, G. Salamone, M. P. Proclamà, R. Guercio, L. Licari, N. Finocchiaro, G. Graziano, G. Orlando, G. Guercio, M. Marcianò, G. Galatioto, F. Vassallo, G. Palmieri, F. Banchini, G. Di Franco, F. Porcelli, N. Furbetta, A. Comandatore, S. Guadagni, M. Palmeri, P. Ubiali, F. Maffeis, J. Velkoski, M. Giuffrida, G. E. Nita, C. Marafante, M. Garino, S. L. Birolo, M. Dugo, M. Pisano, A. Borello, R. Barone, L. D. Bonomo, M. V. Facchino, M. Caccetta, E. Moggia, M. R. D'Anna, C. Mosca, S. Mungo, A. Masciandaro, F. Tirelli, I. Neri, M. Aulicino, C. Vacca, M. Campanelli, M. Grande, L. Siragusa, G. Sica, A. Mingoli, G. Brachini, B. Cirillo, S. Meneghini, S. Giovampietro, G. Duranti, F. Ciccarone, L. Simonelli, I. Clementi, B. Binda, G. B. Fonsi, E. Spalice, E. Cianci, M. I. Bellini, G. Sgarzini, S. Sorrenti, E. Lori, P. Palumbo, D. Pironi, A. Amendola, C. De Martino, E. Bisogno, S. Di Saverio, A. Morello, L. Lely, I. Merlini, G. Travaglini, S. Sabbatini, M. Zambon, D. Giulitti, L. Barni, G. E. Poto, D. Fusario, L. Resca, L. Carbone, A. Francia, G. E. Poto, A. L. Pesce, O. Carpineto Samorani, F. Roviello, A. Ongaro, S. A. Piccioni, M. Gambelli, L. Catozzi, M. Gjoka, A. Bartalini Cinughi de Pazzi, N. N. Leonelle Lore, G. Grassi, F. Manasci, V. Ricchiuti, E. Basile, A. Spaziani, V. Silvestri, M. Favoriti, P. Favoriti, S. Novello, M. Piccino, R. Baldan, U. Grossi, G. Zanus, F. Scolari, E. De Leo, M. Brizzolari, A. Brun‐Peressut, I. Hoxhaj, M. Scopelliti, L. B. Lo Piccolo, E. Montanari, D. Cianflocca, A. Marano, S. Galati, S. L. Gamba, F. Velluti, A. Caltagirone, M. Giuliano, E. Potenza, B. De Zolt Ponte, D. Visconti, V. U. De Donato, L. Capello, S. Chaifouroosh Mamagany, C. Celano, E. Donnarumma, C. Saviello, R. Scola, S. Megna, M. Berselli, N. Palamara, L. Liepa, E. Ferri, V. Gentilino, M. Mogiatti, G. Farris, N. Pasqua, A. Iacomino, I. Mondi, C. Da Lio, F. Sulo, E. Ciccioli, D. Verdi, M. Martorana, M. Filardo, L. Schiavone, I. Conversano, M. Cappiello, G. Scialandrone, N. Petrarota, R. Tumolo, M. Angelucci, S. Valeri, G. Pascarella, A. Strumia, R. Alloni, D. Muschitiello, V. Morinelli, L. Bonello, S. G. Intini, S. Moschella, H. Kato, A. Horiguchi, D. Koike, Y. Asano, S. Alananzeh, S. Al Momani, M. Tanashat, O. Altobaishat, S. Alsmadi, N. Al Rabadi, L. Sweidan, Z. Alnajjar, G. Alsheikh, N. Mosleh, H. Alzuhd, R. Alkhatib, H. Al‐Abdallat, M. Aljarawn, T. Aloqaili, I. Nadi, A. Abdllah, O. Al‐Fahel, R. Khalil, M. Said, A. Qasem, H. Al‐Fahel, M. Hijazi, R. Rabah, A. Alaqtash, D. B. Badwan, Y. Alawneh, B. Alrayes, M. Salah, M. Al‐Qannas, H. Abu Obead, I. Alnimer, Y. Alawneh, M. Almaletti, A. Khamees, R. Yousef Yassin, A. Alsheikh, K. Al‐Shami, E. Abu Siam, O. Sarhan, K. A. Sawaftah, M. A. M. Sawaftah, M. A. Sawaftah, M. Sabri Massadi, Z. Al‐sheikh ali, N. Raiq, O. Ibrahim, J. Al Karmi, M. Diab, I. Aburumman, A. A. Altawaiha, M. Hasan, A. AlZu'bi, L. Yasin, B. Yacoub, R. Abu Salah, M. Alqedrh, S. Abu khousa, M. E. H. Albanna, R. Hussam Yacoub Hattar, D. Samardali, R. Refaie, L. Hijazein, S. Hammad, M. Barbarawi, S. Mamduh, J. Al Daradkah, S. Samardali, T. Alshawabkeh, M. Mahafdah, Q. Sabbah, S. A. M. Ba‐Shammakh, A. Al Hammoud, M. Bani hani, M. Tabaza, H. Malkawi, H. Haj Freej, D. Kasasbeh, B. Dweik, K. Ayyoub, O. R. Mahafdah, R. Hiary, I. Shehadeh, L. Dyab, R. Daradkeh, R. Raddad, R. Alzu'bi, T. Alhaj Hasan, B. Alzoubi, M. Alsharayri, M. Nofal, S. Ellouzy, M. Al‐Masri, S. Fakhouri, N. Absy, R. Suleiman, O. Mansour, J. S. Hadidi, A. Al‐fandi, M. Al‐Fraijat, N. Rabai, A. Al‐Zubeidy, R. Abd Elkareem, S. Bani Amer, T. Alhusban, S. A. L.‐Doghme, R. Abd Alkareem, R. Damseh, M. Alshami, T. Majed, S. Al Sharie, M. Araydah, R. Jaba'Teh, F. Haddad, O. Almomani, L. M. Mheidat, R. Haddad, S. Bataineh, S. Ababneh, M. Kulimbet, N. Maulenov, N. Lakhanov, M. Ramazanov, A. Kiyabayev, D. Amangaliyev, A. Shamsutdinova, A. Polatbekov, R. Parker, E. Irungu, A. Fadipe, F. Ondago, G. Waiyaki, A. Khoneisser, A. Kachi, B. Abboud, M. Chaccour, G. Bechara, N. Eshak, R. Hleyhel, M. Barakat, N. Lindi, B. H. Hameed, A. Abaidalla, N. Mosbah, M. Saleh khatab, M. A. M. Elghriani, A. Ali, A. Fathi, S. Qwyder, M. Saleh, M. Alshamikh, A. Alhammali, A. Aldurssi, A. Gusibat, M. Suleman, S. Alashhab, M. Denini, F. Alowjaly, M. M. Almihashhish, F. Elkhafeefi, H. Altawati, S. Elfallah, F. Benghalbon, R. Michael, M. Abosedra, A. Ahmayda, H. Mftah, M. Bohlala, M. Muragi, S. Alneihuom, A. Alkaseek, A. Alshiteewi, H. Shames, H. Bileid Bakeer, N. Albahloul, M. Abudabbous, A. Belkhair, A. Abdelmalik, M. Assalhi, M. Altajouri, A. Alailesh, A. Alshukre, B. Alazabi, M. Alazabi, G. Birqeeq, A. A. Y. Almugaddami, A. Egdeer, H. Embarek, M. Bilfaqirah, M. Abdelkabir, S. Abdeewi, A. Abdalhadi, M. Benghazi, H. Idheiraj, M. Yahmad, M. Alfaid, M. Abdu, E. Abdu, M. Khalifa, G. Matroud, A. Amaigl, H. Aldare, E. Ali, K. Shwail, K. Abdulrahman, A. Bouhuwaish, A. Emran, A. Abdraba, A. E. Elzoubi, A. Belaid, A. Alragheai, D. Omar, S. Magrhi, H. Farhat, S. Alsuwiyah, S. Abrayik, S. Bensalem, B. Algettawi, S. Egreara, T. M. A. Abdulmola, I. Kandil, F. Alshreef, F. Elhabishi, M. Alsori, L. Shawesh, S. Timmalah, M. Alnuwayli, A. Alhamadi, K. Ahmed Ibrahim, S. Abdullateef, R. Altayargh, A. Essamei, S. Altoume, A. Haidar, M. Khalil, A. Abdulnabi, S. Mohammed, A. Ghummied, E. Younes, S. Elfurdag, A. Ali, S. Ashini, M. Edeeb, Z. Al‐azher El‐hamel, M. Akkawe, N. Alwaer, A. Khair Etareig, B. Allbakosh, H. Abusnina, A. Awidan, L. Alokshi, M. Iqreewi, M. Almahjoub, H. Altounsi, M. Almaqrahi, E. Dainius, S. Bradulskis, E. Margelis, A. Mačiulaitytė, A. Subocius, A. Parseliunas, E. Kubiliute, D. Zuikyte, J. Kutkevičius, J. Vaitekūnas, L. Venclauskas, K. Jasaitis, M. Jokubauskas, Z. Dauksa, A. Gulla, E. Daukšaitė, M. J. Rakotonaivo, C. F. Rahantasoa Finaritra, J. B. Razafindrahita, Y. M. Razafimandimby, A. Rakotondrainibe, A. D. Zakaria, M. I. S. Ismail, R. Noor, M. Che yaacob, S. F. Moh Pauzi, Z. Chin, K. Voon, J. H. Fu, J. H. Lim, S. A. Theivendran, N. N. Ramli, M. Fitri, M. A. Yunus, A. N. Ramly, A. Md Yunos, A. A. A. Anuar, N. S. Abd Ghani, A. A. H. Ahmad Zaidi, F. Ashraf, M. Mahadi, A. A. Abdul Rahim, O. A. A. Dicko, A. Dembele, H. Dolo, D. Kone, C. Cini, A. Sultana, M. Farrugia, J. Schembri Higgans, S. Bowman, J. Psaila, P. Andrejevic, S. Brincat, K. Muscat, M. Sammut, R. Abela, M. Zammit Vincenti, L. Casingena, J. Galea, M. Portelli, M. Sammut, N. Spiteri, K. Iles, R. Cachia, D. Hili, T. R. Ibarra‐Hurtado, L. A. Flores Chávez, J. A. Flores Prado, K. Jasso García, N. E. López Bernal, E. V. Romo Ascencio, L. M. Flores Chávez, M. P. Mellado Tellez, S. A. Ibarra Camargo, G. Delgado Hernandez, J. A. Guzman Barba, L. A. Rea Bocanegra, M. Tello Jimenez, J. A. Tavares Ortega, E. Gómez Mejía, I. Esparza Estrada, A. A. Salinas Barragan, J. A. Jimenez Flores, S. J. Vázquez‐Sánchez, J. Gonzalez Garcia, Z. M. Correa López, F. J. Barbosa Camacho, C. M. Nuño‐Guzmán, A. M. Nava Franco, J. F. Martinez Martin del Campo, J. J. Ulloa Robles, L. Bravo, M. E. Gonzalez‐Gonzalez, F. D. Romo Rosales, T. R. Ibarra‐Hurtado, L. G. Peña Balboa, C. Yanowsky‐Gonzalez, R. Santana Ortiz, S. A. Trujillo Ponce, J. Orozco‐Perez, M. D. C. Gonzalez, J. E. Gonzalez Aboytes, J. Pizarro Lozano, J. E. Orozco Navarro, F. Ibañez Ortiz, O. Montaño Angeles, M. Calderon, F. Diaz, M. Lazo Ramírez, J. A. Aguilar, A. Gonzalez Ojeda, C. Fuentes Orozco, J. M. Chejfec‐Ciociano, J. M. Carranza Rosales, M. A. Sánchez Audelo, C. I. Lupercio Figueroa, K. V. Ascencio Diaz, C. E. Gutierrez de la Rosa, F. Mercado Sanchez, F. Y. González Ponce, R. Mares País, C. González Baez, L. Á. Pelayo Orozco, N. G. Barrera Lopez, A. Ramírez Beas, M. Á. Zaragoza Mendieta, M. F. Zarate casas, S. L. Trejo Ramos, P. Salas Núñez, J. A. Gutiérrez Gómez, G. Ambriz González, I. Cabrera, H. B. Moya‐ Ambriz, F. J. Silva Rivera, E. M. Torres De Anda, A. Hernández, F. J. León Frutos, V. E. Armenta Tapia, M. Nieto Galvan, J. M. Alvarez Hernandez, A. I. Sánchez‐Terán, N. Muñoz Montes, A. N. Fuertes Muñoz, R. L. Smolinski kurek, K. Bozada‐Gutiérrez, A. Nuñez Venzor, A. Zubillaga‐Mares, I. Serrano, C. Moreno‐Licea, S. Anaya Sanchez, A. Trigos Díaz, C. J. Pérez ‐ Padrón, E. Y. García‐Villegas, R. H. Perez‐Soto, M. J. Rueda Medécigo, A. Leon ‐ del‐ Angel, H. D. J. Pérez Baca, C. Chavarría Noya, L. Castro, D. Herappe, M. T. Barrio Renteria, A. Ramos‐De la Medina, L. Martinez, Durán Sánchez, D. S. Gonzalez, M. J. Martínez, M. Berrakkouch, F. Hourri, A. Benmansour, R. Ait Ben Addi, M. Melouane, A. Tariq, O. Boujidi, I. Zerrouq, M. Katif, S. Amahmid, S. Errami, S. Jamil, O. Nouhail, H. Essalim, A. Nidali, N. Ouachou, A. Aboumedian, S. Kessab, N. Lahnaoui, O. Arsalan, S. Kassad, A. Hrora, J. T. Abebrese, M. Van der Colf, F. W. Quayson, P. Shimbulu, P. Nambala, S. Rennie, A. Herewini, S. Kosna, A. Olsen, J. Gillingham, R. Campbell, A. Lin, T. Uiyapat, S. Beavis, D. Bardsley, M. Lill, J. McNab‐Hand, O. Ray, C. Harmston, A. Adeyeye, A. Akinmade, E. Afeikhena, A. I. Okunlola, D. Idowu, J. Olorunfunmi, A. Olabode, N. Oloko, K. J. Bwala, A. Ningi, P. Agbonrofo, D. Osifo, P. Idjerhe, B. O. Izedomi, O. Omoike, O. Irowa, S. Ideh, J. Enaholo, C. Agbonrofo, O. Emuze, P. V. Odigie, M. Ediale, R. A. Eghonghon, M. Edena, A. Ekpeti, M. Momoh, C. Osime, O. Osagie, A. Arekhandia, M. Ibadin, T. AbdulRahman, R. Ediru, J. Abutu John, O. Owolanke, U. Ezomike, N. Agugua‐Obianyo, J. Ede, S. Aliozor, E. I. Nwangwu, C. Ilo, C. Amah, L. Onyebulu, I. Ugwueke, I. Obianyo, C. Onwuzu, U. Dilibe, I. Orji, V. Enemuo, N. Celestine, N. Ekwo, S. A. Sani, S. Olori, I. Pius Ogolekwu, O. Attawodi, R. Hauwa Sani, P. Chimezie Andrew, A. Ishola, O. Ayandipo, N. Akinbami, A. Fakoya, T. A. Lawal, V. Osoka, H. Ogundipe, A. Ademuyiwa, F. Alakaloko, O. Oluseye, N. Duru, M. Ojo, T. Olobatoke, A. O. Lawal, C. Nwanmah, O. Alaba, R. Eloka, C. Bode, O. Elebute, J. Seyi‐Olajide, K. Onyekachi, O. Balogun, O. Christianah, L. Omomeji, F. Akinwande, A. Damola‐Okesiji, J. Okei, H. Abiyere, O. Fatudimu, B. Mustapha, O. Babatunde, A. I. Okunlola, O. M. Williams, O. Faboya, C. Ónyeka, F. Oni, K. Shodunke, G. Eke, M. Abdulsalam, O. Oso, A. Ayodele, M. Okechukwu, C. Adumah, A. Talabi, O. Oyinloye, O. Olajide, V. Agbakwuru, A. Aderounmu, A. Agbaje, Y. L. Balogun, M. O. Ameen, D. O. Komolafe, O. Olasehinde, O. Ajiboye, M. Fagbayimu, G. Aduroja, O. Fasoro, A. Adisa, A. M. Olugbami, H. Oyinlola, E. Adebunmi, T. Mohammed, A. Lawal, F. Bello, P. Adebayo, O. Salako, A. Akinkuolie, M. A. Adetoyi, A. Akeem Aderogba, T. Oyeyemi, O. Ojo, E. Osaze, H. Ekwuazi, I. Ogundele, P. Elemile, A. Ayeni, I. Okoro, C. Onuoha, M. Mobolaji‐Ojibara, J. Mohammad mohammad, N. T. Abdulraheem, A. Jimoh, A. Lawal, O. K. Fasiku, B. Aminu, S. Kache, G. Yohanna Abrak, A. A. Sheshe, L. Anyanwu, A. B. Muhammad, A. Abubakar Abdulkarim, T. N. Nagwamutse, I. U. Garzali, S. Muhammad, C. Nwachukwu, I. E. Suleiman, M. Abdullahi, S. A. Aji, A. Dahiru, L. B. Abdullahi, S. A. Yunusa, A. Yahaya, M. Bello, I. Wasiu, U. Mohammed Bello, B. Yunusa, N. Umar, R. E. Enejo, N. Nwafulume, A. Oke, J. Taiwo, O. H. Ekwunife, O. A. Egwuonwu, O. A. Okoye, N. Nwanne, C. Ugwunne, J. Ugwu, U. Ezidiegwu, C. D. Nwosu, K. Oluchukwu, V. Modekwe, C. Uche, E. A. Obiesie, C. Osuigwe, O. H. Ekwunife, J. Aseme, U. Edith, J. Ezeh, J. Nnoli, H. Willy‐Chidire, D. Chimkaomasiri, A. Ojewuyi, I. Ogundele, A. Adekoya, L. Amosu, A. Oyedele, A. Ayoade, B. A. Ayoade, A. Asekun, A. Ajayi, O. Popoola, M. Yinusa, A. A. A. Oyelekan, O. Oluyemi, C. Nwosu, M. Okudero, M. Agunloye, A. Ojo, C. Nwokoro, I. Babajimi‐Joseph, A. Williams, S. Ogunlade, M. Daniyan, T. T. Sholadoye, M. Bashir, N. Oyelowo, S. E. Nwabuoku, A. Yakubu, O. Ogunsua, M. Abubakar, M. A. Tolani, M. S. Aliyu, T. Risteski, V. Naunova, L. Jovcheski, P. W. Haque, A. Albatanony, A. Fajardo, M. Omer, D. P. Talreja, A. M. Elsayed, H. Habiba, H. AlAamri, F. Ali, S. Alsibai, B. Dawud, J. Albalushi, A. M. Al Balushi, A. Alzadjali, T. Al Barhi, A. Alkharusi, F. Alfarsi, K. Al Hinai, H. Al Qadhi, R. Al Shehhi, H. Al Miskry, O. Al Hamdani, O. Al Alyani, B. Rehmani, D. Ghosh, M. Al‐Attraqchi, Z. Al Balushi, M. Al Hinai, S. Alwardi, L. Al Riyami, A. Al Jamoudi, M. Masaaod, A. Raza, A. Hai, S. Ahmed, D. S. Maqsood, S. Chaudhary, M. Farooq, M. Tayyab, Z. Qureshi, M. Ali, S. Maqbool, U. Abdullah, M. Aziz, A. Irshad, S. Said, A. Zafar, U. Akram, I. Sadiq, A. Abbas, M. Siddique, I. Shahbaz, S. H. Waqar, M. Raheem, F. Akhtar, D. M. Mehmood, D. N. Mahmood, M. Shahid, A. Ali, M. Ahmed, M. Mansoor Iqbal, Y. Lakdawala, S. Otho, M. Khalid, M. Masood, R. Kumar, S. Jabeen, S. Altaf, M. Abdullah, G. Shamsi, I. Ahmed, N. Lodhi, G. Awais, L. Rai, S. Khattak, A. Janjua, A. Liaquat, M. Saleem, M. N. Rafique, M. M. Bin Khalid, H. Ahmad, M. S. Khalid, M. H. Sadiq, A. Hashmi, H. H. Shahid, M. A. Sadiq, M. H. Chishti, M. Usama, M. Kashif, A. M. Choudhary, H. Basharat, K. Khalid, M. A. Haider, M. A. Bashir, H. Sabir, M. F. Tarar, M. Usama, M. B. Mirza, W. U. Rehman, W. Tahir, R. Khalid, C. E. Azmat, H. Qayum, H. Irfan Khan, A. Mustafa, H. W. Bhatti, M. R. Farooqui, F. Rauf, N. A. Malik, M. Usman Malik, S. Hayat, D. Riazhussain, J. Najajra, N. Al‐Hroub, M. Abu Daoud, R. Jubran, M. Srour, M. Taamreh, A. Alsalahat, H. Masalma, A. Alwali, A. Alwali, L. Mohammed, M. Al zebda, S. Mahdi, A. Shaheen, G. Alrayyes, L. Tafesh, T. M. Abubasheer, M. Abu Jayyab, H. Jaber, M. Abuwarda, B. M. J. Alhaj, T. Aldirawi, F. Mahmoud, M. Ali, A. Albhaisi, M. Obaid, W. J. N. Almadhoun, A. Alroobi, M. Abo Abdo, A. Abuthaher, A. AbuNemer, M. Abu Al Amrain, I. Nasser, A. Awad, A. AlAgha, R. Madi, D. AbuNemer, N. Kullab, H. Elhallaq, A. Abu Tair, H. Ayesh, H. Abu‐Arish, A. Zamareh, M. Ahmoud, M. H. Oweidat, I. AlJada, M. Anati, S. Halabi, W. Alhroub, A. Abuhammad, S. M. Udwan, H. Yaghmour, D. Houmran, N. A. Awwad, M. Abed, I. HajMohammed, A. Hewari, A. Alqerem, E. Zidan, H. Abbadi, S. Abed, M. Shakhshir, M. HajHamad, M. Saifi, S. Abuzahra, A. Khouli, Z. Shabello, Z. Khraim, S. Ismail, M. F. Dwikat, R. Bassam, A. Sabbah, A. Gharib, R. Alzughayyar, R. Issa, A. Abuhantash, O. Matar, Y. A. Omar, O. Khalil, A. Awwad, A. Rodriguez Gonzalez, E. D. Sosa Ferreira, R. Ferreira Acosta, M. N. Martínez Bareiro, J. E. Giubi Bobeda, A. Franco, A. Walfor, L. Poggi, L. Poggi, L. Fuentes Rivera Lau, M. A. Moreno Gonzales, F. Camacho, O. Ibarra, G. Arredondo, K. Nieto Yrigoin, D. Chavez Fernandez, D. C. Juan Carlos, G. Mendiola, A. Salazar, R. Casma Bustamante, G. Borda‐Luque, Y. Carpio Colmenares, M. R. Li Valencia, F. Palomino Escalante, K. Quispe de la Roca, M. M. Caramantin Obando, R. Polo, V. Serna‐Alarcon, R. Mazurkiewicz, M. Kołomańska, P. J. Milewski, M. Kisielewski, T. Stefura, K. Richter, W. Wysocki, N. Kłos, W. Jabłoński, I. Alsoubie, B. Żaczek, T. Wojewoda, J. Bolanowski, Z. Orzeszko, M. Wikar, R. Solecki, B. Markowska, M. Szura, T. Gach, M. Matyja, B. Habrat, W. Serednicki, Z. Lorenc, M. Święch, M. Mietła, W. Krawczyk, M. Nycz, K. Urbańska, K. Komorowska, P. Kowalewski, M. Walędziak, J. K. Zajac, M. Zawadzki, M. Kusiński, M. Pryt, F. Brzeszczyński, H. Dąbrowski, M. Redynk, J. Figueiredo, B. Cismasiu, R. Souto, S. Henriques, A. L. Preto Barreira, J. Vaz, J. M. Carlos, M. Trindade, L. Moreira, M. Palas, J. Simoes, F. Ramalho de Almeida, M. Vasconcelos, A. Neves, J. Ribeiro, F. Afonso, A. Pita, R. Miranda Pera, M. Bernardo, C. Rio Ferreira, T. Branco, J. Fontaínhas, S. Pimentel Morais, B. Pinto, S. Patrocínio, L. Moniz, C. Rolo Santos, P. Bernardo, F. Nazareth, C. Silva, L. Heeren, A. R. Mateus Loureiro, B. Tinoco, A. Abreu, D. M. Gonçalves Múrias Gomes, C. Figueiredo, C. Aguero, M. Reia, M. Guerrero, M. A. Fernandez Romero, J. Dominguez, I. Colaço, M. Nunes Luís, S. Andrade, S. Oliveira, D. Pais, D. G. Alves, F. Castro, R. Ribeiro, I. Mogárrio, M. D. C. Gama Caldeira, B. Gama, C. S. Rodrigues, A. Cabral, A. Silva, E. Borges, J. Cassiano Neves, R. Bernardino, P. David Santos, J. Secchi, M. Nunes, D. Tavares, M. Cruz, C. Quintela, C. Cardoso, I. M. Lourenço, D. Vaz Acosta, P. Rego Ponte, R. Santos Pereira, H. Capote, M. B. Mourato, T. Mogne, N. Andrade, G. Fialho, F. Valente Costa Pinto, B. Cordeiro, M. Brito, G. Santos, D. Rosado, C. Costa, N. Pratas, J. DiasFerreira, A. L. Carreira‐Marques, R. Ribeiro Dias, B. Carvalho, M. Gomes, C. Soares‐Aquino, F. Gomes, S. Barbosa Castelo Branco, C. Coutinho, J. P. Vieira de Sousa, D. Atouguia, L. Cidade Costa, D. Silva, P. Correia, C. Henriques, A. M. Pinheiro Pereira, J. Marques Antunes, H. Devesa, R. Barradas, S. Fortuna Martins, N. Marcos, A. Jarimba, B. Louro, L. Rodrigues Madeira, A. R. Lourenço, A. Ferreira, A. Abreu da Silva, D. Stoian, M. Ferreira, R. Branquinho, J. C. Domingues, M. I. Seixo, R. Lalanda, C. Bôto, J. Fernandes, P. Laranjo, M. Reis, I. Borges da Costa, C. Assis, B. Lopes Patrício, N. M. Freitas Oliveira, M. Carvalho, J. Mendes, C. Macedo Cardoso de Oliveira, B. Freire, R. Pinheiro Duque, B. Vieira, U. Fernandes, A. Dupont, J. Ribeiro, R. Vaz Pereira, G. Sarp, E. Soyer Güldoğan, É. Gáspár, A. Chitul, C. Bezede, E. Ciofic, D. Cristian, E. A. Toma, I. M. Matache, O. Enciu, B. Bogdan‐Gabriel, I. Negoi, C. Ciubotaru, I. Tanase, C. Dina, V. M. Negoita, A. Perja, R. Drasovean, A. Trif, D. Misca, C. Hossu, I. Imihteev, V. Kakotkin, M. Agapov, V. Budyakova, S. Dos Santos Rocha Ferreira, R. Senin, A. Bedzhanyan, A. Sumbaev, K. Petrenko, E. Bedzhanyan, E. Tyurina, R. Azimov, P. Glushkov, K. Shemyatovsky, S. Husanov, A. Sidorova, G. Yarovenko, E. Shestakov, O. Lisin, A. Arustamyan, S. Katorkin, J. Sidorovskaia, K. Cholah, I. Cholah, D. Kurochka, V. Ten, Y. Kudryavcev, J. P. Rugambwa, C. N. Nelly Rosine, N. Jeannette, A. Dusabimana, C. Seneza, C. Uwakunda, L. Mukamazera, G. Ntwari, I. Didier, A. Costas‐Chavarri, M. Eugene, C. Nyampinga, R. Munyaneza, D. Muyenzi, N. Alzerwi, M. Rayzah, A. Almutairi, A. Alsultan, B. Ali, A. Shabkah, O. Ibrahim, H. Said, A. Alhebshi, A. Mohsen, K. Anaam, F. Alnazawi, F. Haddad, A. Basalim, B. Albaihani, S. Al Athath, A. Jowharji, A. Aljahdali, N. Trabulsi, M. Alharthi, A. Farsi, M. Ghunaim, A. Nawawi, A. Maghrabi, N. Alzerwi, Y. Aldebasi, F. Al Abbood, A. Elkhalifa, M. Alshanwani, S. Alshagrawi, F. Ahmad, A. Alayed, R. AlQahtani, R. Sugair, O. Alruwaili, B. Alsharari, H. Albalawi, H. Al Sohabi, A. Alshahrani, S. Asiri, M. Alshehri, A. Albalawi, R. Alatawi, T. Khewater, H. Adi, J. Akiely, N. Musawa, F. Alahmad, B. Alqahtani, M. Sersarah, Z. Farraj, D. Y. Alalawi, A. Alzahrani, N. Al Amri, M. AlThomali, D. Elkafrawy, J. Juloski, V. Cuk, V. Cijan, L. Milic, J. A. Košir, J. Grosek, A. Tomazic, T. Košir Božič, S. Gumede, C. Kloppers, K. Booyse, S. Dos Santos, M. Flint, Z. Johnson, J. J. Jordaan, G. Steenkamp, K. Nieuwenhuys, J. Uys, S. S. Verhage, A. Goliath, S. Gilbert, M. Kariem, N. Karimbocus, C. Lategan, T. Mabogoane, S. Mewa Kinoo, R. Naidoo, N. Ntanzi, S. Sibiya, S. Ebrahim, S. Govender, E. Naidoo, P. Moodley, K. Maharaj, H. Le Roux, J. Van Niekerk, A. Sparke, P. Omwansa, C. A. Baars, S. Marawu, K. Sevnaran, A. Szpytko, G. Charalambous, B. Van Zyl, O. Pheiffer, F. Roodt, D. Rattray, N. Rasool, M. Nkogatse, R. Mackay, G. Urdang, V. Manchev, D. Clarke, S. Naidu, V. Govindasamy, D. Montwedi, L. C. Kolongi, C. Elliot‐Wilson, S. Kalenga, I. Serfontein, M. Goga, S. Burger, R. Duvenage, H. Aguado López, F. Ruescas, A. García Marín, M. Scortechini, M. Jurado Román, A. Sanchez Gallego, E. González Marín, S. De la Cruz Ahufinger, M. Mateu, M. J. Medina, M. D. M. Martí‐Ejarque, R. Soliva Domínguez, L. Ruiz‐Villa, E. Montalbán Martínez, A. Torroella, C. Ginesta, G. Cárdenas Rivera, V. E. Gonzabay, J. D. Acevedo Parrales, M. Canals Sin, A. Lombardero, B. Capdevila Vilaró, M. Carbonell Pradas, M. E. Muñoz Fernández, R. A. Hernandez Rodriguez, I. De Haro Jorge, M. Riba Martínez, L. Tapia Moral, M. Coronas Soucheiron, P. Palazon Bellver, L. Ortega Lechuga, X. Tarrado, A. Domenech Plana, J. Prat‐Ortells, M. Bejarano Serrano, M. Cuesta Argos, M. P. Martin Gimenez, S. G. Laura, R. Ripoll i Palmés, A. Sainz Lete, J. C. Zevallos‐Quiroz, D. Gómez, B. Estraviz, J. M. De Francisco Rios, J. Barrutia Leonardo, M. González de Miguel, R. L. Ferlini, M. Ortega Escudero, Y. Galvañ Félix, C. Hernandez Diaz, J. Montero García, Á. Fernández Camuñas, E. P. Garcia Santos, F. J. Redondo Calvo, M. Estaire Gómez, R. J. Castro Lara, A. Ramos Bonilla, L. Rodríguez Gómez, M. Marqueta De Salas, A. Alvarez Cuiñas, F. M. Bujalance Cabrera, M. D. Cancelas, A. García Domínguez, G. Chamoso Mialdea, E. P. Cagigal Ortega, D. Enjuto, I. Cervera, R. Villalobos Mori, Y. Maestre González, C. Gas, L. Codina Corrons, C. Semeraro, J. García‐Quijada, T. W. Jorgensen, L. Marquez, M. J. Peña Soria, N. Tabatabaian, J. L. Garcia galocha, D. Fra Corral, L. Sante Serna, M. Diez Alonso, C. Vera Mansilla, L. Casalduero, S. Soto Schütte, Y. Allaoua, E. Gutierrez, C. Zapata Syro, F. Prieto La Noire, M. D. M. Olmedo Reinoso, S. Salido, N. Chavarrias, M. Vicario Bravo, L. Asensio Gomez, R. Abad, A. Gegúndez Simón, P. C. Arteaga Asensio, A. M. Minaya Bravo, E. González, A. Galvan, C. Guijarro Moreno, G. De la Peña González, A. Sánchez Gollarte, A. Robin Valle de Lersundi, M. Á. García Ureña, J. L. Rodicio Miravalles, A. A. Suárez Álvarez, D. W. Silva‐Cano, G. Martínez Izquierdo, P. Del Val Ruiz, M. Moreno Gijon, S. Amoza Pais, G. P. Ibero Casadiego, E. López‐Negrete Cueto, J. Carrizo, S. Sanz, R. Rodríguez‐Uria, A. Cembellin, G. García‐Santos, A. Fraile, D. Córdova García, L. Jiménez, J. Martin Fernandez, R. Alvarado Hurtado, A. M. Minaya Bravo, R. Díaz Pedrero, N. Cobeño Tamayo, V. Ongil Rodríguez, F. Aguilar del Castillo, Á. De Jesús Gil, S. Borrego Canovaca, J. R. Naranjo Fernández, Z. Valera Sanchez, R. Perez, M. Infantes Ormad, M. Sánchez Ramirez, C. Leal Ferrandis, C. Esteo Verdu, S. García López, J. Febré, B. Cuneo, C. León‐Espinoza, E. Martí Cuñat, G. Pou, C. Jezieniecki, S. Alonso Marcos, A. Vazquez Fernandez, J. Beltrán de Heredia, B. De Andrés‐Asenjo, D. Baños Méndez, J. C. Garcia Vera, M. Ruiz Soriano, E. Redondo, R. Martínez Díaz, T. Gómez Sanz, P. Artigot, C. Infante, C. Ferreras García, L. R. Cabezudo, G. Cabezudo, M. Lainez Escribano, M. Rodriguez‐Lopez, H. Nuñez Del Barrio, A. Romero de Diego, A. Vazquez Melero, M. Camuera, I. Herrero, D. Garcia López de Goicoechea, M. Sánchez‐Rubio, M. D. P. Cebollero, J. L. Blas Laina, V. Duque Mallén, N. Sánchez Fuentes, P. Sancho Pardo, I. Gascon Ferrer, M. Á. Dobón Rascón, T. Gimenez Maurel, J. Chóliz, S. Saudí‐Moro, S. Paterna ‐Lopez, A. Martinez German, D. Aparicio‐López, M. Á. Gascón Domínguez, P. Royo Dachary, S. Srishankar, S. P. B. Thalgaspitiya, K. J. Senanayake, D. Wickramarathna, D. Subasinghe, D. Wickramasinghe, M. Nandasena, K. Wijesinghe, H. Miyasika, J. Senavirathna, Y. Chamara, S. Rajendra, S. I. Thuraisamy Sarma, B. Balagobi, V. Sutharshan, S. Giridaran, W. Wijenayake, M. T. Ekanayake, S. Jayatilleke, S. Jayasekara, M. Perera, R. Perera, R. Ellawala, W. D. D. De Silva, S. Alqurashi, N. Rajab, T. A. Albushary, F. A. Mohammed Daoud, A. Younis, M. A. Suliman, F. Tahir Lwdie, S. Ibrahim Tour Harakan, M. Abdelhadi Suliman Adam, E. Hegab, A. Abdalla, T. Almahdi, E. Alabed, M. Ahmed, S. Eldirdiri, M. Salah, O. A. Eljizoly, A. Mohammed, A. Mohamed Ibrahim Mohamed, A. Albager, M. A. Ismael Alamin, M. Alsalawi, M. A. Elgak, E. G. Nubi Mohamed, A. Eltahir, E. Adel Hamdoun Aziz, I. Adel, O. Emadeldeen, O. G. Nubi, E. Mohamed, M. Hamed, M. Tageldin, E. Elsheikh, U. Omara, E. Adam, I. M. G. Ahmed, G. M. G. Ahmed, S. Imam, A. A. Adam, S. Amin Omar Alsiddig, A. Ahmed, S. Abdelrasoul Elnour Ismail, M. Mohamedshafee, Y. Mohamed, E. E. Abuobaida Banaga Hag El Tayeb, H. Abuobaida, A. S. Ahmed, A. Elbalal, I. M. G. Ahmed, G. N. El Hunjul, G. M. G. Ahmed, A. Mustafa, H. A. Fadlalmola, A. Mohammed, E. Yousuf, E. Hamed, S. Ibrahim, O. Morgan, N. Omer, M. Zaigham, A. Al Mukhtar, M. Nikberg, D. Fenner, D. Salinovic, X. Papazarkadas, T. V. Pham, C. Brasset, A. Litchinko, F. Ris, C. Golliez, M. Chevallay, J. Gass, J. Mühlhäusser, J. Metzger, A. Scheiwiller, A. Tampakis, C. Riboni, U. Dietz, E. Brolese, C. Seiler, M. Kalisvaart, J. N. Marx, L. Eisner, G. Peros, F. Solimene, M. Gramellini, A. Lareida, M. Adamina, E. Betz, L. Dubs, K. Geiger‐Timm, L. Gantner, N. Seeger, K. Richetti, K. Hofmann, M. A. Schneider, D. Gero, K. Lehmann, P. Limani, S. Hügli, S. Gerdes, F. Mazzola, A. Hiller, M. A. Farho, M. Mohammad, A. Y. Arnaout, Y. Nerabani, Y. Maktabi, W. Alsado, A. Anadani, M. Morjan, M. Nasani, W. Mayo, S. Kreid, M. Arnaout, M. N. Sawas, M. Aloulou, M. K. Marawy, A. Kezze, I. Arnaout, A. Kelzia, A. Ghazal, A. Ghazal, E. Dabbagh, R. Masri, M. H. Nabhan, A. Alniemi, A. Alhaj, S. Ward, Y. Haido, N. Dadoush, M. K. Abu albahrain, A. Niazi, W. Abbas, A. Hasan, S. Alshab, S. Kamari, R. Kalouk, Z. Toutounji, D. Sharl Ajami, B. Alsaid, A. Alusef, K. Abo zaal, A. R. Hammadieh, Z. Klib, M. R. Mslmani, A. Alhaj zain, M. Klib, L. Shammas, A. J. Chekfa, Z. Odeh, R. Joumaa, H. Al‐zoubi, Q. Mashlah, H. O. Odah Bashi̇, H. Zwaraa, I. Adham, L. Hasan, A. Khatib, S. Jomaa, A. Alfarwan, A. Torbey, A. Rashid, A. Hawarah, A. Ali, M. Alhimyar, L. Al‐Boukhari, M. A. Al‐yusuf, A. N. Aldirani, Y. Alhammoud, Y. Al‐Junaidi, M. Daher, Z. Asaad, A. Abbas, K. Khalil, J. Khoury, L. Hasan, J. Jahjah, J. Alaji, S. Turkmani, S. Mahfoud, A. Ahmad, M. Derattani, G. Massarra, J. Skaff, Z. A. Hannouneh, H. Amoudi, Z. A. Zaher, G. Zaza, A. Wassouf, A. Alahmad Alismael, S. Nofal, A. Mansour, M. Mansour, Z. Alkhaier, J. Suliman, M. Sabboh, M. Haj Hussein, M. Ibrahim, Z. A. Abo alaros, G. Alhadwah, N. Kheyrbek, D. Ibrahim, G. Hamdan, Y. Hasan, A. Abo al Shamat, A. Roumieh, B. Khattab, H. Alkhatib, S. Hassan, S. Abdul Rahman, A. Abdul Rahman, F. Aliskander, J. Alkharish, G. Kafa, I. Suleiman, A. Bassma, A. Alloush, N. Ismaiel, J. Deeb, M. Alrantisi, E. Salloum, A. AlMouahhed, A. Baydoun, H. Yunes, S. Alkadi, F. Ali, D. Abdulrahman, I. Hussein, A. Bakri, H. Asaad, T. Ashkar, H. Daaboul, A. Marouf, F. Chahrour, B. Ranjous, B. Ibrahim, A. Sinjab, M. Alneasan, N. Ali, A. Alloush, J. Fahed, R. Attaf, S. Kanaan, A. Tansawet, W. Kasetsermwiriya, I. Laopeamthong, P. Sukhvibul, T. Techapongsatorn, N. Techapongsatorn, P. Kasetsermwiriya, P. Leungon, P. S. Tekam Wadje, M. J. Kacem, R. Elaifia, Y. Ouadi, S. Megdiche, Y. Jedidi, A. Ç. Bozkurt, H. Tümer, M. A. Koç, A. Çakmak, A. F. Kocaay, K. Y. Türker, T. B. Türkmen, O. Yalkin, D. Yigit, B. Yigit, A. Aslan, S. Yilmaz, A. N. Sanli, Y. İ. Tandoğan, A. Yildiz, A. İsler, A. Ozkomec, M. E. Seker, E. Ay, M. Erkaya, Y. O. Koyluoglu, A. Develioğlu, G. M. Kurtoglu, G. K. Kurtoglu, A. F. Cetişli, E. Tunçcan, A. Aghayeva, Z. Durna, B. Baca, A. E. Dönmez, B. Togay, E. Ada, I. E. Yavuz, I. A. Bilgin, E. C. Karabulut, A. M. Uysal, Y. Karataş, B. Ağca, M. K. Aktas, F. Demiral, B. Duman, K. Kabulov, I. Hamzaoglu, T. Karahasanoğlu, M. Tanal, E. Tuzuner, S. Meriç, M. Tokocin, H. Yigitbas, A. Barcin, G. Alici, E. Yavuz, O. B. Gülcicek, N. Bugdayci, K. Özdoğan, I. Çakir, Y. E. Aktimur, Y. Altinel, Ö. P. Zanbak Mutlu, R. E. Sönmez, M. Şermet, M. S. Ozsoy, H. Baysal, F. Buyuker, M. Oncel, S. Bektas, A. E. Askin, M. Yashar, A. İzgi̇ş, S. S. Uludağ, M. F. Ozcelik, S. Yumurtacilar, A. Özcan, E. Somuncu, S. Yilmaz, A. Sapmaz, H. Bolukbasi, C. Özkan, E. Bozdağ, M. C. Kizilkaya, H. Telci, Y. Kara, A. Z. Kaan, M. Acar, E. O. Yildirim, G. Akcakoca, C. Hacialioğlu, Y. Tosun, V. Çalik, T. E. Yilmaz, H. Alfakeer, M. I. Ateş, S. N. Karahan, M. Kalender, A. E. Narin, D. Yi̇ği̇t, O. Agcaoglu, S. Toprak, B. Celik, E. Ozoran, D. S. Uymaz, S. Yigman, E. Bozkurt, A. Rencuzogullari, E. Balik, S. Sucu, A. Akmercan, K. Oğur, A. Hajali, Q. K. Dolatzay, E. Unal, N. Kiziltoprak, M. S. Genç, B. Özcan, Z. Şenol, O. F. Ozkan, M. Çuhadar, E. D. Terzi, T. Gülşen, M. T. Demirpolat, B. Citgez, H. Ozsahin, C. Ersavas, C. Bi̇li̇r, A. E. Boztaş Demi̇r, A. D. Hacioglu, G. Ozyuksel, H. Ulman, A. M. Öztürk, B. Calik, A. C. Yaşar, E. Colak, M. A. Avci, E. Aybar, M. Gün, A. B. Ciftci, M. S. Uyanik, M. E. Kara, C. Akgün, A. C. Sari, Ö. Küpçüoğlu, G. O. Kucuk, S. Polat, N. Kavak, M. A. Kara, G. Karadeniz Cakmak, B. Kum, S. Öztürk, B. Eyduran, B. Kigwe, M. Arafat, M. Nnabagulanyi, S. Stonelake, Z. A. Fozo, A. Shoker, K. R. Rahman, M. W. Saqib, R. Faisal, C. S. Ong, A. Pillai, H. Unwin, A. Huws, M. Maybury, H. Ejaz, E. Daketsey, A. K. Lala, M. A. K. Sarker, B. Chkir, M. R. Peris, S. Khan, M. A. Tahir, N. Sharma, R. Doherty, H. Alhusaini, M. F. Butt, H. Afzal, S. Handa, N. Maharjan, A. Mostafa, G. Lee, C. K. Lim, A. Anand, A. Krishna, W. T. Yew, Y. Lu, R. Hall, E. Mohammed, F. Georgiades, S. Karim, K. Rajaratnam, J. Abbasy, A. Bibi, S. Karandikar, L. Johnstone, N. Fazili, A. Singh, A. Athanasiou, H. Lidbetter, J. Siby, M. Kaur, A. Fatima, S. M. Reddy, A. Campbell, A. Cardoso Almeida, K. Smith, C. J. Bradshaw, K. Tambudze, H. Delacave, I. Norman, M. San, S. Babu, S. Midya, H. Bradly, S. Tontus, H. Chauhan, R. Jurdon, M. Corcos, E. Jose, N. Eardley, B. Davies, M. Ransome, S. Ahmed, S. Suresh, A. Bavaharan, R. Batir, R. Sato, N. Chidumije, M. Ahmed, T. Y. Kwan, F. Olaniru, I. Parwaiz, L. P. Cheng, N. Gokhare Viswanath, K. Nanayakkara, A. Tibude, O. Olajumoke, M. Shams, D. George, A. Amin, M. Kausar, S. Sellahewa, H. Kamal, A. Kamal, M. Kamal, O. Pryer, H. Sagar, B. Lulham‐Robinson, S. Dawo, A. Nada, R. Bethune, G. Chillarge, A. M. Myintmo, M. Horga, L. Andreski, E. Ruiz‐Daum, T. Finlay, I. Rakshit, J. Bryan, D. Joshi, R. Marlin, M. Battili, Y. L. Aung, L. Zeng, S. Mathew, I. Njere, S. Gopaul, A. Abbas, G. Singh, M. Asarbakhsh, S. Staight, R. Govindaraju, M. Quaunine, S. Yassin, A. Wilkins, J. Walshaw, L. Chang, A. Mahendran, F. Hammett, D. Fairbrass, U. A. Kalu, E. Dexter, T. Nadeem, N. Karunaratne, T. Lo, M. Pellen, S. H. Sarwary, J. Otote, D. Anbu, H. Islam, N. Morricone, K. Lee, E. Gimson, M. Wilson, C. Chiam, M. Solkar, M. Bautista, N. S. Blencowe, A. Gupta, J. Sutcliffe, A. Ahmad, A. Peckham‐Cooper, E. O'Connell, K. E. Dey, R. Lunevicius, M. M. Barakat, G. R. Goodwin, B. Devkaran, I. C. Nzenwa, A. Pilavas, K. Bananis, A. Alamin, S. Bennett, M. D. Barcelona, A. Sharp, F. Soggiu, E. Baili, H. Ebied, A. Botha, M. Haghighat Ghahfarokhi, M. M. T. Youssef, K. Theodoropoulou, A. Quddus, R. Hegy, M. Mahran, A. Ghanbari, P. Kapsampelis, T. Chouari, J. Saunders, C. Boven, I. Gerogiannis, N. Karthikeyan, C. Karagianni, E. Spanoudakis, H. Younus, R. Ben Hmida, D. Eaton, C. Seet, R. Bradley, R. Roberts, F. N. Amir, M. Durrani, S. Khan, A. Tahir, A. Khalil, E. O'Neill, S. Ingley, V. Bill, P. Wilson, M. Elmousili, E. Chin, C. Alphonse, P. Suresh, L. Devi, C. Shelton, S. Bugren, M. Abdelreheem, M. M. R. Azzuz, F. Gareb, D. J. Dhillon, M. F. Khan, B. Peter, I. Fagiri, T. Harris, P. Thambi, M. Tomlinson, C. Hidalgo Salinas, A. Mwanjoka, M. Catterall, B. Ali, S. Tingle, K. Waddell, F. Peters, T. Akharaekpanya, S. Robinson, N. A. Binti Yusri, A. Ashiru, S. A. Chowdhury, J. Reilly, S. Malek, S. Kumaran, A. Doghaim, A. B. A. AlHajjaj, L. R. Chieng, Z. Y. Wong, R. Olatunji, J. Bundred, B. Down, A. Ang, K. A. Shamiyah, G. Bond‐Smith, M. Elmesalmi, S. Monkhouse, O. Mohamed, B. Robertson‐Jones, P. Patel, B. Z. Hao, S. Ghattas, N. Beharry, A. Maraqa, N. Uttam, S. Rafiq, A. Abdelhamid, E. Mazumdar, M. Dyer, M. E. E. A. Abdelsalam, B. Johnson, M. Abdelkarim, A. Murtada, M. M. S. Tora, Z. Azhar, O. Whitehurst, K. Bhatti, S. Silvestre, L. N. Bin Aizan, A. Patel, Z. Khan, A. Maqsood‐Shah, O. Webster, H. Reilly, S. Boyes, S. Bandyopadhyay, B. McDermott, H. Kynaston, B. Neall, M. West, G. Hart, R. Titcombe, H. Kaur, A. Ekerin, A. Demetriou, N. Harrison, J. J. Q. Chen, M. Hammoda, L. Robine‐Durnell, E. Mansour, A. Evans, E. Baker, I. Abdullah, O. Osunlusi, S. Soelling, R. Askari, M. Asaad, J. Leong, M. Perkins, D. Ozal, S. S. Budhwani, A. Maxwell, A. Shah, S. Schimpke, D. Moris, C. Nicholson, H. E. Rice, D. Ridder, M. Tsuruta, K. Noguchi, D. Mikami, R. Kitamura, J. Ng‐Kamstra, R. J. Robitsek, K. Fretwell, J. Chan, R. Laskowski, R. Zerna Encalada, R. Ghaleb, N. Alnamari, A. Al‐Bahla, B. Al soudi, B. Alshaikh, M. Al‐Shehari, M. Al‐Dhaheri, Y. S. S. M. Ghaleb, M. I. Issa

**Affiliations:** ^1^ Department of Applied Health Sciences, School of Health Sciences University of Birmingham UK

**Keywords:** anaesthesia, developing countries, inguinal hernia, surgery

## Abstract

**Introduction:**

Restoration of surgical capacity is essential to post‐COVID‐19 recovery. This study explored the use and safety of anaesthesia options for inguinal hernia surgery, a common tracer condition, to describe current global practice and highlight opportunities to build the capacity of health systems.

**Methods:**

This is a secondary analysis of an international prospective cohort study of consecutive patients who underwent elective inguinal hernia surgery. We used a consensus process to define generalisable outcomes to measure patient selection, utilisation of hospital capacity and peri‐operative safety in patients who received locoregional, spinal or general anaesthesia for their surgery.

**Results:**

In total, 16,554 patients from 83 countries were included. Locoregional anaesthesia was performed in 1536 (9.2%) of patients, compared with 9165 (55.4%) who had general and 55,853 (35.4%) who had spinal anaesthesia. Patient selection outcomes were comparable across anaesthesia groups. As a measure of hospital capacity, adjusted day‐case rates were higher for locoregional anaesthesia (OR 6.62, 95%CI 5.13–8.54, p < 0.001) but not for spinal anaesthesia (OR 0.97, 95%CI 0.84–1.12, p = 0.68) compared with general anaesthesia. Complications were lower in patients who underwent locoregional anaesthesia (OR = 0.67, 95%CI 0.52–0.87, p = 0.001) but not for spinal anaesthesia (OR = 0.90, 95%CI 0.77–1.05, p = 0.167) compared with general anaesthesia after risk adjustment.

**Discussion:**

This study has filled knowledge gaps of anaesthesia practice in common surgeries across the world. Locoregional and spinal anaesthesia could be adopted as safe options to increase surgical volume when there is limited access to general anaesthesia.

## Plain Language Summary

After the COVID‐19 pandemic, it's important to get hospitals back to doing regular surgeries again. This study looked at the types of anaesthesia (medicine that helps patients not feel pain during surgery) used during a common operation called inguinal hernia surgery. The goal was to see how safe the different types are and how they are being used around the world. The study used information from another big study that followed patients from many countries who had planned hernia surgeries. The researchers looked at three types of anaesthesia: general anaesthesia (makes you sleep during surgery); spinal anaesthesia (numbs the lower part of your body); and locoregional anaesthesia (numbs just the area being operated on). The researchers compared how doctors chose which anaesthesia to use and if patients had any problems after surgery. The study looked at 16,554 patients from 83 countries: 9% had locoregional anaesthesia; 55% had general anaesthesia; and 35% had spinal anaesthesia. People who had locoregional anaesthesia were more likely to go home the same day. People who had locoregional anaesthesia also had fewer problems after surgery. Spinal anaesthesia didn't show big differences compared to general anaesthesia in those areas. This study helps us understand how anaesthesia is used for a common surgery around the world. It showed that locoregional and spinal anaesthesia can be safe choices, especially in places where general anaesthesia is harder to get. Using these types can help hospitals do more surgeries safely and quickly.

## Introduction

The COVID‐19 pandemic reduced elective surgical capacity and led to a backlog of cases [1]. This has added to the global burden of surgical morbidity, with an estimated 143 million people in low‐ and middle‐income countries (LMICs) now in need of elective surgery [2]. Patients waiting for elective surgery are more likely to suffer from disability and present as an emergency, with increased pressure on services and risk to patients [3]. To meet the global surgical burden, the volume and availability of elective surgery must be increased safely.

Anaesthesia workforce constraints remain one of the main limitations to upscale global surgical capacity [4, 5]. Low‐income countries (LICs) have an estimated 0.3 anaesthetists per 100,000 population, far fewer than the recommended 20/100,000 [2, 6]. Within LMICs, first referral hospitals are less likely than referral hospitals to have a full‐time anaesthetist [7]. Anaesthetic delivery is further constrained by infrastructure, medication availability and hospital bed capacity [8, 9].

Several solutions have been proposed to address these constraints. Non‐physician anaesthetic providers may share some tasks (e.g. spinal anaesthesia) for selected cases, although appropriate supervision models from medically trained anaesthetists are essential [10]. For example, task sharing of spinal anaesthesia by non‐anaesthetic physicians was shown to be non‐inferior to spinal anaesthesia delivered by consultant anaesthetists for patients who underwent selected surgical procedures in India [11]. Spinal anaesthesia is popular in resource‐limited settings as it requires minimal equipment and is safe [8]. Previous work suggests that surgeons could share workload with anaesthetists through delivery of local‐only anaesthesia for carefully selected cases, with appropriate monitoring escalation plans in place [12]. To date, however, few studies have explored international variation in anaesthesia delivery across common surgery types.

We proposed that inguinal hernia surgery was an appropriate tracer condition to research global anaesthesia practice; this procedure can be performed under general, spinal or locoregional anaesthesia. Inguinal hernias are common and contribute substantially to surgical waiting lists [13, 14, 15]. This study aimed to describe global delivery of anaesthesia for inguinal hernia surgery. To capture patient and system‐level data, we assessed composite outcomes of patient selection, capacity utilisation and peri‐operative safety across anaesthetic modalities. This was a secondary analysis of a previously described international prospective cohort study of inguinal hernia surgery [13].

## Methods

Full details on ethical approval are available from the published study protocol [16]. In brief, where possible, this study was registered as a clinical audit. If formal ethical approval was required, the local principal investigator sought this according to national and hospital regulations. No data were uploaded until proof of appropriate study registration was shown. Informed patient consent was obtained in hospitals that required it. A reflexivity statement for global health research is available in online Supporting Information Appendix S2.

An international, multicentre prospective cohort study of patients who underwent inguinal hernia surgery was conducted. Full methods including robust data management procedures have been reported previously [16]. In summary, any hospital that performed inguinal hernia repair was considered eligible. For this secondary analysis, consecutive patients of any age and ASA physical status 1–4 who underwent elective primary laparoscopic, robotic or open inguinal hernia repair between 30 January and 21 May 2023 were included. Open surgeries via a midline incision were not included due to the added complexity of surgery.

Anaesthetic delivery was defined as general anaesthesia (including inhaled or total intravenous anaesthesia); spinal anaesthesia; and locoregional anaesthesia (including locally infiltrated anaesthetic or regional blocks). For this study, anaesthesia was classified by primary mode of delivery. We did not collect data on anaesthesia failure or cross‐over. Sedation‐only surgeries were not included due to low numbers and incomplete data on airway management for these cases. Anaesthesia provider was binary, defined as either ‘operating surgeon’ or ‘anaesthetist/anaesthetic nurse/anaesthetic technician’ (undifferentiated). The study did not further differentiate between clinical and non‐clinical providers of anaesthesia.

A steering group, consisting of a diverse network of anaesthetists and peri‐operative clinicians from high‐, middle‐ and low‐income countries, was formed to define outcome measurements. The steering group was constructed using members of the National Institute for Health and Care Research Global Health Unit on Global Surgery network, who have participated in previous research outputs [17, 18]. Outcomes were organised into three domains: patient selection; utilisation of hospital bed and workforce capacity; and peri‐operative safety. Measures of patient selection included patient and disease‐related factors used commonly in decision‐making about anaesthesia modality, such as age; sex; ASA physical status; comorbidities; hernia size; and indication for surgery. Day‐case rates as a primary outcome and anaesthetic administrator grade as a secondary outcome were chosen as proxies of hospital capacity to deliver surgery. Measures of safety were assessed at 30 days and included Clavien‐Dindo score for overall complication rate [19]; postoperative infection; or re‐operation. Surgical site infection was assessed according to the US Centers for Disease Control and Prevention definition, as described previously [20].

Non‐parametric data were explored using the Mann–Whitney U‐test and parametric data analysed using two‐tailed Student's *t*‐test or one‐way analysis of variance depending on the number of comparator groups. Categorical variables were presented as frequencies and proportions and were analysed using the χ^2^ test. Countries were mapped to country income groups defined by the World Bank: LICs; LMICs; upper‐middle income countries (UMICs); and high‐income countries (HIC). We also classified countries by their Human Development Index (HDI), a summary metric of health, standard of living and education indices [21]. This is a holistic metric to describe development, reflecting investment in human capital. The Human Development Index ranges from 0.4 to 1.0; countries < 0.550 are classified as low HDI; 0.550–0.699 as medium HDI; 0.700–0.799 as high HDI; and > 0.800 as very high HDI [21]. Multilevel logistic regression models were created using hospitals within countries as random effects, and results presented with 95%CIs. Statistical analysis was performed using R (Version 4.02, R Foundation for statistical computing, Vienna, Austria). A p‐value of < 0.05 was considered statistically significant.

## Results

**Figure 1:**
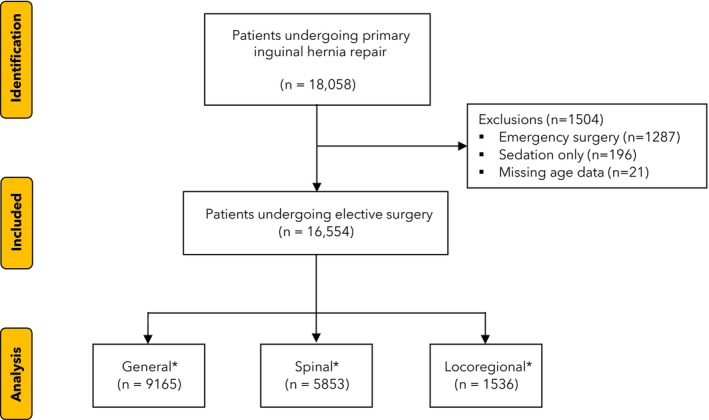
Flow chart of included patients. Patients with sedation only (without local anaesthesia) not included due to low numbers and as we were unable to ascertain if they underwent tracheal intubation.

**Figure 2:**
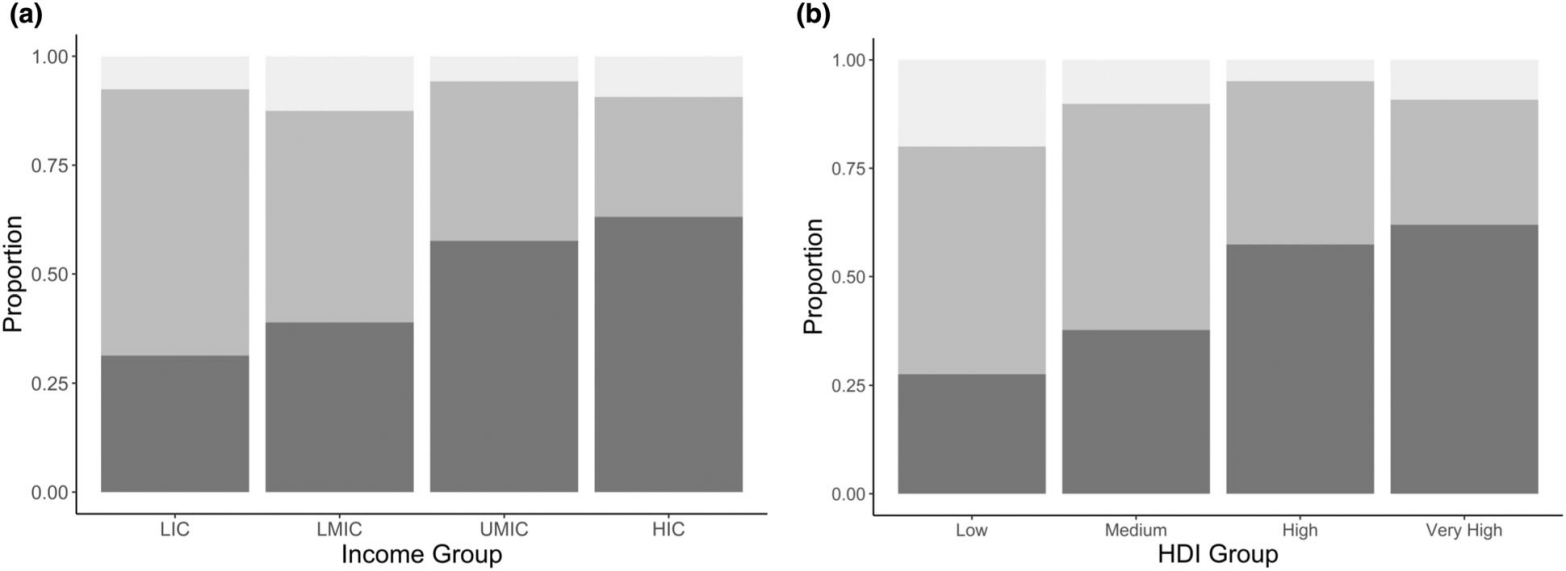
Anaesthesia delivery against (a) income group and (b) HDI group. Darkest shading, general anaesthesia; mid shading, spinal anaesthesia; light shading, locoregional anaesthesia; LIC, low‐income countries; LMIC, low‐ or middle‐income countries; UMIC, upper middle‐income countries; HIC, high‐income countries; HDI, Human Development Index.

The study included data from 16,554 patients from 640 hospitals in 83 countries (Fig. 1). Overall, 9165/16,554 (55.4%) of patients had general, 5853/16,554 (35.4%) had spinal and 1536/16,554 (9.24%) had locoregional anaesthesia. General anaesthesia was more common in HICs and UMICs while spinal was more common in LMICs and LICs (Table 1, Fig. 2). Tertiary hospitals (838/10,663, 7.9%) and private hospitals (60/833, 7.2%) were less likely to perform inguinal hernia surgery under locoregional anaesthesia than other hospital types. Total intravenous anaesthesia was used more commonly in HICs (online Supporting Information Table S1). Mean (SD) age was similar across general, spinal and locoregional anaesthesia (47.3 (26) y, 56.5 (17.9) y and 57 (18.6) y, respectively); however, children aged < 16 were much more likely to have general anaesthesia than other modalities (online Supporting Information Table S2). Comorbidities and ASA physical status were comparable across anaesthetic groups; most patients had no comorbidities and were ASA physical status 1–2 (Table 2). Hernia size was also very similar across anaesthetic modalities, with most limited to the inguinal region.

**Table 1:** Country and hospital characteristics across types of anaesthetic. Values are number (proportion).

	General	Spinal	Locoregional	Total
	n = 9165	n = 5853	n = 1536	n = 16,554
Income group				
LIC	252 (2.7%)	491 (8.4%)	61 (4.0%)	804 (4.9%)
LMIC	1382 (15.1%)	1720 (29.4%)	445 (29.0%)	3547 (21.4%)
UMIC	1771 (19.3%)	1127 (19.3%)	177 (11.5%)	3075 (18.6%)
HIC	5760 (62.8%)	2515 (43.0%)	853 (55.5%)	9128 (55.1%)
HDI group				
Low	295 (3.2%)	562 (9.6%)	214 (13.9%)	1071 (6.5%)
Medium	924 (10.1%)	1275 (21.8%)	248 (16.1%)	2447 (14.8%)
High	1670 (18.2%)	1096 (18.7%)	143 (9.3%)	2909 (17.6%)
Very high	6269 (68.5%)	2920 (49.9%)	931 (60.6%)	10,120 (61.2%)
Hospital type				
Primary	765 (8.4%)	404 (7.0%)	184 (12.0%)	1353 (8.3%)
Secondary	2321 (25.6%)	1551 (26.7%)	510 (33.3%)	4382 (26.7%)
Tertiary	5975 (65.9%)	3850 (66.3%)	838 (54.7%)	10,663 (65.0%)
Hospital funding				
Public	7422 (81.9%)	4889 (84.2%)	1374 (89.7%)	13,685 (83.5%)
Private	1136 (12.5%)	646 (11.1%)	98 (6.4%)	1880 (11.5%)
Public–private	503 (5.6%)	270 (4.7%)	60 (3.9%)	833 (5.1%)
Hospital payment				
Other	344 (3.8%)	272 (4.7%)	46 (3.0%)	662 (4.0%)
Other insurance	970 (10.7%)	236 (4.1%)	79 (5.2%)	1285 (7.8%)
Government insurance	7206 (79.5%)	4737 (81.6%)	1199 (78.3%)	13,142 (80.1%)
Cost borne by patient	541 (6.0%)	560 (9.6%)	208 (13.6%)	1309 (8.0%)

LIC, low‐income countries; LMIC, low or middle‐income countries; UMIC, upper middle‐income countries; HIC, high‐income countries; HDI, Human Development Index.

**Table 2:** Measures of patient selection across types of anaesthetic. Values are mean (SD) or number (proportion).

	General	Spinal	Locoregional	Total
	n = 9165	n = 5853	n = 1536	n = 16,554
Age; y	47.3 (26.0)	56.5 (17.9)	57.0 (18.6)	51.4 (23.3)
Age groups				
< 16	1780 (19.4%)	123 (2.1%)	37 (2.4%)	1940 (11.7%)
16–40	1051 (11.5%)	903 (15.4%)	224 (14.6%)	2178 (13.2%)
40–60	2608 (28.5%)	1906 (32.6%)	510 (33.2%)	5024 (30.4%)
60–80	3255 (35.5%)	2504 (42.8%)	634 (41.3%)	6393 (38.6%)
> 80	468 (5.1%)	414 (7.1%)	131 (8.5%)	1013 (6.1%)
Sex; male	8127 (88.7%)	5410 (92.4%)	1385 (90.2%)	14,922 (90.2%)
ASA physical status				
1–2	8004 (87.4%)	5112 (87.4%)	1349 (87.8%)	14,465 (87.4%)
3–4	1115 (12.2%)	726 (12.4%)	164 (10.7%)	2005 (12.1%)
Not recorded	44 (0.5%)	14 (0.2%)	23 (1.5%)	81 (0.5%)
Comorbidities				
0	7342 (80.1%)	4514 (77.1%)	1240 (80.8%)	13,096 (79.1%)
1	1415 (15.4%)	1026 (17.5%)	219 (14.3%)	2660 (16.1%)
2	309 (3.4%)	251 (4.3%)	63 (4.1%)	623 (3.8%)
≥ 3	92 (1.0%)	57 (1.0%)	12 (0.8%)	161 (1.0%)
Missing	7 (< 0.1%)	5 (< 0.1%)	1 (< 0.1%)	13 (< 0.1%)
Hernia size				
Limited to inguinal region	7362 (80.3%)	4333 (74.0%)	1212 (78.9%)	12,907 (78.0%)
Limited to scrotum	1749 (19.1%)	1425 (24.4%)	310 (20.2%)	3484 (21.1%)
Extend to mid‐thigh or beyond	52 (0.6%)	94 (1.6%)	14 (0.9%)	160 (1.0%)
Asymptomatic hernia	1718 (18.7%)	896 (15.3%)	279 (18.2%)	2893 (17.5%)

**Figure 3:**
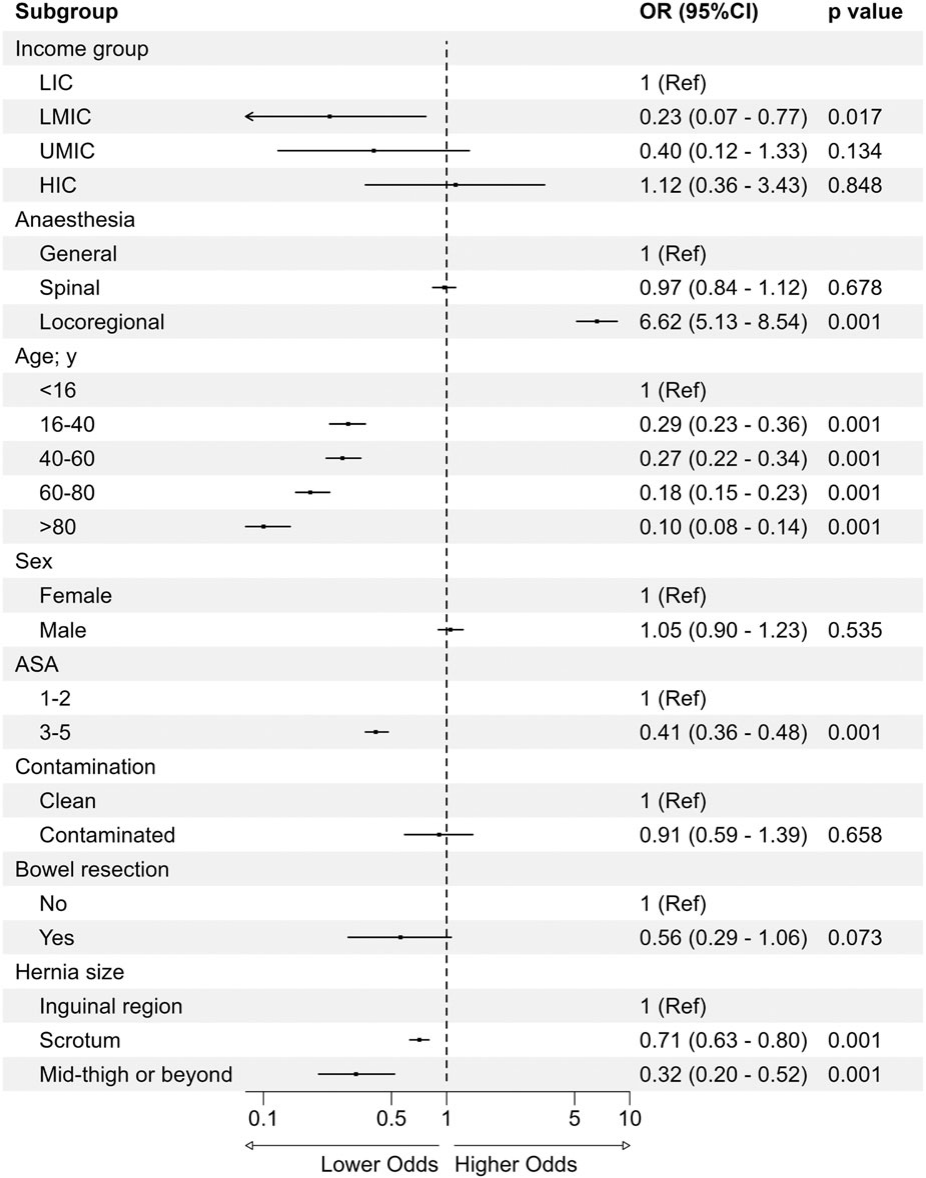
Multilevel logistic regression model showing odds ratios for day‐case rates. LIC, low‐income countries; LMIC, low‐ or middle‐income countries; UMIC, upper middle‐income countries; HIC, high‐income countries.

Most patients who had locoregional anaesthesia went home the same day (1151/1536, 75.1%); day‐case rates were lower in patients who had general anaesthesia (5036/9165, 55.0%) and spinal anaesthesia (2626/5853, 45.0%) (Table 3). Compared with general anaesthesia, patients receiving locoregional anaesthesia were significantly more likely to undergo day‐case procedures (OR 6.62, 95%CI 5.13–8.54, p < 0.001), while those with spinal anaesthesia were less likely (OR 0.97, 95%CI 0.84–1.12, p = 0.68) (Fig. 3). In 1087/1536 (70.8%) of patients, locoregional anaesthesia was performed without an anaesthetist, compared with 422/9165 (4.6%) in general and 257/5853 (4.4%) in spinal anaesthesia (Table 3). Complications between patients who had a surgeon or anaesthetic provider deliver anaesthesia were comparable when grouped by anaesthetic modality (online Supporting Information Table S3).

**Table 3:** Peri‐operative outcomes across types of anaesthetic. Values are number (proportion).

	General	Spinal	Locoregional	Total
	n = 9165	n = 5853	n = 1536	n = 16,554
Day‐case	5036 (55.0%)	2626 (45.0%)	1151 (75.1%)	8813 (53.3%)
Anaesthetic administrator				
Anaesthetist*	8742 (95.4%)	5596 (95.6%)	449 (29.2%)	14,787 (89.3%)
Surgeon	422 (4.6%)	257 (4.4%)	1087 (70.8%)	1766 (10.7%)
Clavien‐Dindo complications	991 (10.8%)	859 (14.7%)	178 (11.6%)	2028 (12.3%)
1	764 (8.3%)	688 (11.8%)	132 (8.6%)	1584 (9.6%)
2	148 (1.6%)	130 (2.2%)	36 (2.3%)	314 (1.9%)
3a	32 (0.3%)	24 (0.4%)	9 (0.6%)	65 (0.4%)
3b	42 (0.5%)	15 (0.3%)	0	57 (0.3%)
4a	2 (< 0.1%)	0	1 (0.1%)	3 (< 0.1%)
5 (death)	3 (< 0.1%)	2 (< 0.1%)	0	5 (< 0.1%)
Postoperative infection	197 (2.2%)	232 (4.0%)	62 (4.0%)	491 (3.0%)
Re‐operation	54 (0.6%)	24 (0.4%)	3 (0.2%)	81 (0.5%)

* Includes anaesthetists, anaesthetic nurses and anaesthetic technicians.

Most patients experienced no complications (14,503/16,554, 87.6%). Crude complication rates between locoregional (178/1536, 11.6%), general (991/9165, 10.8%) and spinal (859/5853, 14.7%) anaesthesia were compared (Table 3). Multilevel logistic regression showed that locoregional anaesthesia had a significant reduction in complications (OR = 0.67, 95%CI 0.52–0.87, p = 001). Spinal anaesthesia did not have a significant reduction in complications (OR = 0.90, 95%CI 0.77–1.05, p = 0.167) compared with general anaesthesia, after adjustment for: income group; age; ASA physical status; contamination; bowel resection; mesh use; and hernia size (online Supporting Information Figure S1). Re‐operation rates were very low (81/16554, < 1.0%) and comparable across all groups.

Sevoflurane was the most common anaesthetic volatile agent used in HICs (2570/3107, 82.7%), UMICs (1059/1386, 76.4%) and LMICs (748/1239, 60.4%), but LICs used more halothane (84/199, 42.4%) than sevoflurane (76/199, 38.2%) (online Supporting Information Figure S2). Nitrous oxide use was very low across all country groups, ranging from 7/1386 (0.5%) in UMICs to 61/3107 (2.0%) in HICs (online Supporting Information Table S4).

## Discussion

This study shows a wide variation of anaesthetic delivery across the world for a single procedure. Overall, use of locoregional anaesthesia was low, although patients who had this were less likely to stay in hospital overnight or have complications. This adds to existing literature suggesting that locoregional and spinal anaesthesia are safe options for selected patients and can boost global surgical capacity by reducing inpatient admissions [15, 22, 23, 24, 25]. Further work is required to prospectively evaluate the selection, safety and acceptability of locoregional and spinal anaesthesia at scale.

In LICs, more inguinal hernia surgeries are performed under spinal than general anaesthesia. This finding is supported by other studies in LMICs, particularly for obstetric surgery [8, 26]. Spinal anaesthesia in low‐resource settings may be preferred by providers as it is safe and requires less equipment, such as ventilation, and by patients due to lower costs [27, 28, 29]. In our study, patients given spinal anaesthesia were less likely to go home the same day, reducing bed capacity and increasing the chance of future elective list cancellation. However, this finding may be confounded by unmeasured factors, such as limited planned day‐case lists in these regions.

In HICs, use of general anaesthesia was more common than spinal. General anaesthesia makes use of greenhouse gases, which contribute to climate change [30]. Of all anaesthetic volatile agents, desflurane has the highest global warming potential, 2540 times more than carbon dioxide and 20 times more than sevoflurane. In HICs, 11% of cases used desflurane, highlighting an urgent need to complete the transition to less polluting agents such as sevoflurane. Fortunately, our study also showed low global usage of nitrous oxide, which likewise has a high global warming potential, 273 times higher than carbon dioxide [30]. Halothane, an older anaesthetic agent which can cause liver injury, is still more common in LICs. This is particularly concerning as Piramal Pharma Ltd, one of the largest suppliers to sub‐Saharan Africa, has recently stopped halothane production [31].

Global variation and low levels of locoregional anaesthesia use are likely due to surgeon, anaesthetist and patient preference [32]. Locoregional anaesthesia may be perceived by clinicians and patients to increase the risk of peri‐operative pain, although this is not supported by the evidence [33, 34]. Regional block is a skill that needs to be learned and thus may not be suitable for all surgeons and practitioners. Target nerves, such as ilioinguinal, iliohypogastric and genitofemoral, follow aberrant courses frequently, and can be difficult to locate [35]. Adjuncts such as ultrasound can help the efficacy of the regional block, but this requires additional resources and training [36, 37]. Additionally, surgeons may prefer general or spinal anaesthesia; inguinal hernia surgery has a steep learning curve, and training is easier when the patient has received neuromuscular blocking drugs. This may be a particular barrier in HICs, where training opportunities are increasingly limited [38]. This study did not assess patient preferences; this is a key factor and requires more exploration globally.

Our study shows that 70% of cases using locoregional anaesthesia were performed by surgeons, although we are unable to comment on whether continuous intra‐operative monitoring was performed, or on the availability of an anaesthetist in case of patient safety issues. Shared anaesthesia provision, for example with surgeon‐delivered locoregional anaesthesia, may help circumvent workforce bottlenecks and free up senior anaesthetists to cover multiple operating theatres or support more complex procedures [39, 40, 41]. This may be particularly beneficial in LMICs, which have the lowest total number and lowest density of anaesthetists, well below the recommended 20 per 100,000 population, and the greatest burden of unmet surgery [2, 42].

In addition, our study showed relatively large numbers of surgeons who provided general and spinal anaesthesia. While this may represent practice in very remote regions with severe workforce constraints, supervision of all modes of anaesthesia delivery by trained anaesthetists is essential to ensure peri‐operative safety and provide alternative anaesthetic options if required. Many countries have begun to ‘task‐shift’ anaesthesia care to non‐physician anaesthesia providers [10], with variability in safety and supervision models around this. Although guidance advocates for supervision of non‐physicians by physician anaesthetists, non‐physician anaesthesia providers may act unsupervised in some LMICs, due to the limited anaesthesia workforce [43, 44, 45]. Further research is needed to understand how current anaesthesia provision by physician and non‐physician anaesthesia providers in these regions impacts patient selection, capacity and safety.

While external stressors on health systems such as COVID‐19 increased delays and cancellations for elective surgery, it is possible the pandemic had a divergent effect on anaesthesia capacity in some settings. Retraining and redeployment of the healthcare workforce into anaesthesia and critical care roles and strengthening of infrastructure (e.g. for piped oxygen supply) may have boosted net overall capacity for peri‐operative services in some hospitals [46]. Despite this, many health systems are still to reach pre‐pandemic levels of activity [47].

This study has both strengths and limitations. We present, to our knowledge, the largest prospective cohort study describing the variety of anaesthetic practice in inguinal hernia surgery. This captures data from a wide range of healthcare facilities and countries. However, the study over‐represents tertiary referral hospitals; primary and secondary referral hospitals perform the most elective surgery and are the least resourced. Our pragmatic cohort study was not able to collect specific anaesthetic complications (e.g. haematoma, local anaesthesia toxicity). However, the Clavien‐Dindo classification system is agnostic to causes of adverse events and captured both surgery and/or anaesthesia‐related complications. Patients were not contacted routinely 30 days following surgery and readmissions were not recorded so some complications may have been missed. However, major complications resulting in re‐admissions were recorded. Due to heterogeneity in country participation, different practices might be over‐represented. This study also did not capture data on conversion from local to general or spinal anaesthesia; presence of intra‐operative monitoring; or postoperative pain. These factors may be influenced by anaesthesia technique, and/or patient and healthcare worker preferences. Additionally, we did not record whether patient tracheas were intubated; the grade of anaesthetist; whether anaesthesia was delivered by a non‐physician or non‐specialty trained physician; or the selection criteria used when deciding the modality of anaesthesia. More detailed exploration of the safety of non‐physician anaesthesia providers is a timely and important focus for future research. We used a pragmatic definition of general anaesthesia which did not mandate the use of cuffed tracheal tubes; this may have meant that some patients given intravenous drugs did not have a protected airway. To address this, we did not include patients who were coded as ‘sedation only’.

In conclusion, this study describes measures of patient selection, capacity and safety across global anaesthesia delivery for a single common general surgical procedure. Future studies are required to determine if local and spinal anaesthetic procedures are acceptable to patients and can be upscaled to potentially increase capacity. There is an opportunity for reverse innovation and learning about anaesthetic delivery from the global south to north. Surgical volume may be increased by the adoption of context‐sensitive approaches that may include locoregional anaesthesia and/or spinal anaesthesia lists in some regions.

## Supporting information


Plain Language Summary.

**Appendix S1.** Authorship.

**Appendix S2.** Reflexivity statement.

**Figure S1.** Multilevel logistic regression model showing odds ratios for complications against types of anaesthetic.
**Figure S2.** Map showing halothane use globally.

**Table S1.** Country and hospital characteristics across anaesthetic groups.
**Table S2.** Country, hospital and patient selection, capacity and safety outcomes across paediatric age groups.
**Table S3.** Subgroup table of complications in locoregional anaesthesia across anaesthesia provider, grouped by anaesthetic type.
**Table S4.** Anaesthetic volatile agents and gases across income groups.
