# Supplementary material for: Global anaesthesia practice using inguinal hernia surgery as a tracer condition: a secondary analysis of an international prospective cohort study
Source: Anaesthesia. 2025 Sep 9;80(11):1343–51. doi: 10.1111/anae.16686 (PMC12519944; doi:10.1111/anae.16686)
Supplement: Supplementary file 1 — Plain Language Summary. [file ANAE-80-1343-s001.docx]

**Plain Language Summary**

After the COVID-19 pandemic, it’s important to get hospitals back to doing regular surgeries again. This study looked at the types of anaesthesia (medicine that helps patients not feel pain during surgery) used during a common operation called inguinal hernia surgery. The goal was to see how safe the different types are and how they are being used around the world. The study used information from another big study that followed patients from many countries who had planned hernia surgeries. The researchers looked at three types of anaesthesia: general anaesthesia (makes you sleep during surgery); spinal anaesthesia (numbs the lower part of your body); and locoregional anaesthesia (numbs just the area being operated on). The researchers compared how doctors chose which anaesthesia to use and if patients had any problems after surgery. The study looked at 16,554 patients from 83 countries: 9% had locoregional anaesthesia; 55% had general anaesthesia; and 35% had spinal anaesthesia. People who had locoregional anaesthesia were more likely to go home the same day. People who had locoregional anaesthesia also had fewer problems after surgery. Spinal anaesthesia didn’t show big differences compared to general anaesthesia in those areas. This study helps us understand how anaesthesia is used for a common surgery around the world. It showed that locoregional and spinal anaesthesia can be safe choices, especially in places where general anaesthesia is harder to get. Using these types can help hospitals do more surgeries safely and quickly.
