# Supplementary material for: Global anaesthesia practice using inguinal hernia surgery as a tracer condition: a secondary analysis of an international prospective cohort study
Source: Anaesthesia. 2025 Sep 9;80(11):1343–51. doi: 10.1111/anae.16686 (PMC12519944; doi:10.1111/anae.16686)
Supplement: Supplementary file 2 — Appendix S1. Authorship. [file ANAE-80-1343-s004.docx]

**Appendix S1 Authorship**

## Authorship Group

*International writing group*

Cortland Linder (UK)*, Maria Picciochi (UK)* Sakina Bhaloo (UK); Ebenezer Amofa (Ghana); (Canada Gaston (Rwanda) Jose Andres Calvache (Colombia) Sivesh Kamarajah (UK) Palesa Motshabi (South Africa) Dmitri Nepogodiev (UK), Dhruv Ghosh (India); Laura Kudrna (UK); Maame Jenny (Ghana); Muyiwa Rotimi (Nigeria); Telesphore Kabera (Rwanda); Virginia Ledda (UK); Sam Lawday (UK), Craig McClain (USA); Cliff Shelton (UK); Abdul Ghaffar (Switzerland or Pakistan); Adedogni (Nigeria); Allen Jean De La Croix Ingabire (Rwanda);; Asad Latif (Pakistan); Nana Boateng (Ghana); Jean Pierre Nganabashaka (Rwanda); Larissa Cronje (South Africa); Mogane Palesa (South Africa); Sylvestre Nzahabwanayo (Rwanda); Pritpal Singh (India); Revatrams (India); Rotimi Aaron (Nigeria); Samuel Jerry Cobbina (Ghana); Sandrine Zola (Benin); Suryakiran Sharma (India); Teena Gill (India); Tony Thomson (India); James Glasbey (UK); Janet Martin (Canada) ^$^; Christina George (India) ^$^; Aneel Bhangu (UK) ^$^

*Joint first authors, ^$^Joint senior authors

*Statistical Analysis*

Cortland Linder (UK), Maria Picciochi (UK), Sivesh Kamarajah (UK), Bryar Kadir (UK), James Glasbey (UK), Aneel Bhangu (UK)

Operational Committee:

Aneel Bhangu (UK), Abdul Gaffar (Pakistan), Adewale Adisa (Nigeria); Andrew Dove (UK); Christina George (Unknown); Dmitri Nepogodiev (UK); Elizabeth Li (Unknown); Ewen Harrison (UK); Fareeda Galley (Ghana); Ian Thomson (UK); J.C. Allen Ingabire (Rwanda); James Glasbey (UK); Janet Martin (Canada); Laura Kudrna (UK); Mwayi Kachapila (Malawi); Natalie Rowland (UK); Omar Omar (Somalia or Kenya); Parvez Haque (Bangladesh or India); Pritpal Singh (India); Rachel Lillywhite (UK); Richard Lilford (UK); Rob Lillywhite (UK); Samuel Cobbina (Ghana); Sylvestre Nzahabwanayo (Rwanda); Telesphore Kabera (Rwanda); Tracy Roberts (UK).

Dissemination Committee (listed by country): Albania: I Dajti; Algeria: Z Djama; Argentina: M Lucchini, RM Palacios Huatuco; Australia: K Atherton, AC Dawson, E Lun; Austria: F Aigner; Belgium: F Berrevoet; Benin: I Lawani, S Lawani, C Bokossa; Bosnia and Herzegovina: S Delibegovic; Bulgaria: M Slavchev; Burkina Faso: AF Sanon, A Sanou; Burundi: JB Gusa, JC Mbonicura; Cameroon: A Bang, O Gabom, C Nwegbu; Canada: A Brar, J Martin; Chile: MM Modolo, M Olivos; Colombia: JA Calvache; Croatia: J Mihanovic; Cyprus: N Gouvas, A Yiallourou; Czech Republic: B East; Dominican Republic: S Batista, R Rivas; Ecuador: EP Lincango; Egypt: S Emile; Ethiopia: AB Aregawi; France: AP Arnaud; Gabon: N Boumas; Georgia: Z Demetrashvili; Germany: H Lederhuber, MW Löffler; Ghana: AE Agbeko, NB Sam, S Tabiri, F Agyei, FE Gyamfi, S Mohammed; Greece: I Katsaros, G Tsoulfas; India: L Bains, J Dhiman, D Ghosh, PD Haque, A Suroy; Ireland: S Ramjit; Israel: G Marom; Italy: F Pata, G Gallo; Jordan: F Ayasra; Kazakhstan: I R Fakhradiyev; Kenya: IHS Hamdun; Kyrguzstan: A Iqbal; Liberia: E Mbanzabugabo; Libya: M Elhadi; Lithuania: A Gulla; Madagascar: L Samison; Malawi: M Nyirenda, R Nyirenda; Malaysia: AC Roslani; Mali: B Bengaly; Malta: J Psaila; Mexico: L Martinez, A Ramos-De la Medina; Namibia: PR Nashidengo; New Zealand: M McGuinnes, D Wright; Niger: A Ousseini; Nigeria: A Adisa, AO Ademuyiwa; North Macedonia: T Risteski; Oman: Z Al Balushi, B Dawud, A AlSharqi, F Ali; Pakistan: AU Qureshi; Palestine: H Abu-Arish; Paraguay: H Gomez-Fernandez; Philippines: JM Faylona, MD Sacdalan; Poland: W Krawczyk; Portugal: JG Goncalves-Nobre, M Sampaio-Alves, I Santos; Romania: I Negoi; Russian Federation: A Butyrskii; Rwanda: JC Allen, F Ntirenganya; Sierra Leone: I Fortune; Slovenia: J Kosir; South Africa: N Parker, K Chu; Spain: A Minaya Bravo; Sri Lanka: D Wickramasinghe, U Jayarajah; Sudan: M Elmujtaba; Sweden: M Nikberg; Switzerland: E Gialamas; Syria: M Alshaar; Tanzania: M Nkoronko; Turkey: A Isik; Uganda: I Mubesi; United States: J NgKamstra; Venezuela: O Bahsas-Zaky.

Hospital leads (listed by country and city): Albania: E Agastra (Korca; Regional Hospital of Korca); I Dajti (Tirana; University hospital Koco Gliozheni). Algeria: R Belouz (Algiers; CHU Isaad Hassani); Z Djama (Constantine; University Hospital Abdelhamid Ben Badis); A Mouffokes (Oran; EHU-1st November 1954). 2 Argentina: ME Muriel (Allende, Cordoba; Sanatorio Allende - Sede Cerro); RM Palacios Huatuco (Buenos Aires; Hospital Italiano de Buenos Aires); M Santillan (Buenos Aires; Hospital Universitario CEMIC); A Duro (Buenos Aires; Hospital municipal de vicente lópez); JI Valenzuela (City of Buenos Aires; Hospital Velez Sarsfield); DA Pantoja Pachajoa (Cordoba; Clinica Universitaria Reina Fabiola); G Romero reyna (Cordoba; Sanatorio Allende - Sede Nueva Cordoba); CM Florián Villa (San Francisco; Clinica Regional del Este). Aruba: M Gosselink (Oranjestad; Dr. Horacio E Oduber Hospital). Australia: YH Lam, A Nguyen (Adelaide; Northern Adelaide Local Health Network); JA Duffield (Adelaide; Royal Adelaide Hospital); A Frankel (Brisbane; Princess Alexandra Hospital); S Bowman (Brisbane; Queen Elizabeth 2 Jubilee Hospital); D Mitchell (Brisbane; Surgical Treatment and Rehabilitation Service); H Iswariah (Brisbane; The Prince Charles Hospital (TPCH)); S Abeykoon (Campbelltown; Campbelltown Hospital); S Gananadha (Canberra; Calvary Hospital); S Gananadha (Canberra; Canberra Hospital); S Salindera (Coffs Harbour NSW; Coffs Harbour Health Campus); S Stevens (Colac; Colac Area Health); AC Dawson (Gosford; Gosford Hospital); M Issa (Hamilton; Hamilton Base Hospital); E Wong (Melbourne; Angliss Hospital); L Bromley (Melbourne; Austin Hospital); E Wong (Melbourne; Box Hill Hospital); K Jaffry (Melbourne; Casey Hospital); M Bickford (Melbourne; Knox Private Hospital); E Wong (Melbourne; Maroondah Hospital); R Nataraja (Melbourne; Monash Childrens Hospital); R Hodgson (Melbourne; Northern Hospital); A Fox (Melbourne; St Vincent’s Hospital); M Wichmann (Mount Gambier; Mount Gambier and Districts Health Service); A Davis (Mount Nasura; Armadale Health Service); S Zhang (Newcastle; Calvary Mater Newcastle); M Ishak (Newcastle; John Hunter Hospital); R Liang (Robina; Robina Hospital); P Tang (Sale; Sale Hospital / Central Gippsland Health Service); R Liang (Southport; Gold Coast University Hospital); M Park (Sydney; Royal North Shore Hospital); C Cornwell (Sydney; The Royal Prince Alfred Hospital); E Page-Taylor (Toowoomba; Toowoomba Hospital); A Hameed (Westmead; Westmead Hospital); R McGee (Wyong; Wyong Public Hospital). Austria: I Königsrainer (Feldkirch; Landeskrankenhaus Feldkirch); F Aigner (Graz; Barmherzige Brüder Krankenhaus, Graz); S Mikalauskas (Graz; Medical University of Graz); L Havranek (Linz; Ordensklinikum Linz Elisabethinen); A Binder (Tulln; Universitätsklinikum Tulln). Bangladesh: S Islam (Dhaka; Dhaka Medical College Hospital); MP Singh (Dinajpur; Lamb Hospital). Benin: G Gbessi (Cotonou; Centre National Hospitalier et Universitaire Hubert Koutoukou Maga); H Aouagbe Behanzin (Cotonou; Hopital de Menontin); M Agbadebo (Dassa-Zoumè; Hôpital de Zone de Dassa-Zoumè); E Bara (Kandi; Hôpital de zone de Kandi); AB Yevide (Klouékanme; Hopital de Zone de Klouékanme); TK Hessou (Natitingou; Centre Hospitalier Départemental de l’Atacora); AM Hodonou (Parakou; Centre Hospitalier Universitaire Borgou Alibori); I Lawani (Porto Novo; Centre Hospitalier Universitaire et Departemental Oueme Plateau). Bosnia and Herzegovina: Z Matkovic (Doboj; Genera Hospital ‘Sveti aposto Luka’ Doboj); N Lalovic (Foča; University Hospital Foča); J Miskovic (Mostar; SKB University Clinical Hospital Mostar); M Salibašić (Sarajevo; Clinical Center University of Sarajevo); A Cerovac (Tešanj; General Hospital Tešanj); A Tursunovic (Tuzla; University Clinical Center Tuzla). Brazil: C Panis (Francisco Beltrão; Universidade Estadual do Oeste do Paraná). 3 Bulgaria: T Ivanov (Pleven; Heart and Brain - Pleven Hospital); M Karamanliev (Pleven; University Hospital Dr Georgi Stranski, Medical University - Pleven); R Donchev (Plovdiv; MHAT St. Karidad); D Hadzhiev (Plovdiv; UMHAT Sveti Georgi); T Yotsov (Ruse; University Hospital Medika); E Hristova (Sofia; Fifth City Hospital Sofia - 5th MBAL). Burkina Faso: AF Sanon (Ouagadougou; Tengandogo University Hospital). Burundi: JC Mbonicura (Bujumbura; Centre Hospitalo-Universitaire de Kamenge); N Diomede (Bujumbura; Kamenge Military Hospital); J Gusa (Bujumbura; Prince Regent Charles Hospital). Cambodia: S Stock (Battambang; Handa Medical Centre). Cameroon: O Ndizeye (Bamenda; Nkwen Baptist Hospital). Canada: R Spence (Halifax; Queen Elizabeth II); S Lee (Port Moody; Eagle Ridge Hospital); S Lee (Vancouver; Royal Columbian Hospital). Chile: MM Modolo (Santiago; Hospital Barros Luco Trudeau). China: W Yang (Guangzhou; The First Affiliated Hospital of Jinan University). Colombia: CJ Perez Rivera (Bogota; Fundacion Cardioinfantil-IC); S Sierra (Medellin; Clínica CES); DS Garcés Palacios (Popayan; Hospital Susana Lopez de Valencia); JA Calvache (Popayán; Hospital Universitario San José). Croatia: J Mihanovic (Zadar; Zadar General Hospital); G Augustin (Zagreb; University Hospital Centre Zagreb). Cyprus: A Yiallourou (Nicosia; Nicosia General Hospital). Czech Republic: J Moravik (Decin; Krajská zdravotní as - Hospital Decin); A Al Kaddah (Hradec Kralove; Charles University Hospital); Z Musilová (Ivančice; Hospital Ivančice); M Schön (Novy Jicin; Hospital & Oncological Centre Novy Jicin); J Roman (Ostrava; University Hospital Ostrava); B East (Prague; Motol University Hospital). Dominican Republic: R Rivas (Santo Domingo; CEDIMAT - Centro de Diagnóstico, Medicina Avanzada, Laboratorio y Telemedicina). Egypt: G Abouelnagah (Alexandria; Alexandria Main University Hospital); D Attia (Alexandria; Alexandria Medical Research Institute); G Abouelnagah (Alexandria; Smouha University Hospital); A Maher (Assiut; Assiut University Children Hospital); A Kedwany (Assiut; Assiut University Hospital); S Abdelmohsen (Aswan; Aswan University Hospital, Aswan University); R Adel Diab (Cairo; Al Zahraa University Hospital); A Al-Mallah (Cairo; Al-Azhar University Hospitals); H Taher (Cairo; Cairo University Children’s Hospitals (CUSPH & CUCH)); H Abozied (Cairo; EL-Hussein University Hospital, Al-Azhar University, Faculty Of Medicine); ASM Abdelrahman (Cairo; Giza International Hospital); M ElFiky, N Shehata (Cairo; Kasr Al Ainy Faculty of Medicine, Cairo University); B Fahmy (Giza; The Memorial Soaad Kafafi University Hospital); H Elghadban (Mansoura; Mansoura University Hospital); E Adel Mahmod Sultan (Menofia; Menofia University Hospital); M Omar (Qena; Qena University Hospital); EA Ahmed (Sohag; Sohag University Hospital); AM Elkhouly (Tanta; Tanta University Hospital); A Asla (Zagazig; Al Ahrar Zagazig Teaching Hospital). Ethiopia: F Terefe (Addis Ababa; Yekatit 12 hospital medical college); A Yeshitila (Deberebirhan; Hakim Gizaw Hospital); M Taeme (Dessie; Dessie Referral Hospital); A Demessie (Gondar; Gondar University Comprehensive specialized hospital); AB Aregawi (Hawassa; Hawassa University 4 Comprehensive Specialized Hospital); N S.Bayleyegn (Jimma; Jimma University Medical Center); B Sime (Yirgalem; Yirgalem Hospital Medical College). France: A Police (Eaubonne; Hôpital Simone Veil); A Castaldi (Nimes; Hôpital Carèmeau); E Reitano (Strasbourg; Nouvel Hopital Civil de Strasbourg). Gabon: N Boumas (Libreville; Centre Hospitalier universitaire mère enfant Fondation Jeanne Ebori). Georgia: Z Demetrashvili (Tbilisi; N.Kipshidze Central University Clinic). Germany: C Kamphues (Berlin; Park-Klinik Weissensee); D Hackner (Erlangen; Universitätsklinikum Erlangen); U Ronellenfitsch (Halle; University Hospital Halle); J Rolinger (Moenchengladbach; Kliniken Maria Hilf); D Reim (Munich; Klinikum Rechts der Isar TUM School of Medicine); N Börner (Munich; Ludwig Maximilian University of Munich - Großhadern); J Goedeke (Munich; Ludwig Maximilian University of Munich - Innenstadt); AE Gut (München; Isarklinikum); M Janda (Rostock; University Hospital Rostock); J De Deken (Saarbruecken; Klinikum Saarbruecken); MW Löffler (Tuebingen; University Hospital Tuebingen). Ghana: R Armah (Accra; Greater Accra Regional Hospital); A Bediako Bowan (Accra; Korle-Bu Teaching Hospital); E Kafui Ayodeji (Accra; Pentecost Hospital); EA Arkoh (Ankaful; Ankaful Leprosy General Hospital); FE Gyamfi (Berekum; Berekum Holy Family Hospital); A Davor (Bolgatanga; Upper East Regional Hospital); MT Morna (Cape-Coast; Cape Coast Teaching Hospital); N Agboadoh (Damongo; St. Anne’s Hospital); EA Nachelleh (Ho; Ho Teaching Hospital); AE Agbeko (Kumasi; Komfo-Anokye Teaching Hospital); S Mensah (Kumasi; University Hospital, KNUST); P Taah-Amoako (Nsawkaw; Tain District Hospital); K Collins (Sunyani; Brong-Ahafo Regional Hospital); AS Seidu (Tamale; Tamale Teaching Hospital); BK Seshie (Tema; Tema General Hospital); G Ansong (Walewale; Walewale Government Hospital). Greece: K Kambouri (Alexandroupolis; Alexandroupolis University General Hospital); A Kyriakidis (Amfissa; General Hospital of Amfissa); D Korkolis (Athens; Agios Savvas Anticancer Hospital); N Memos (Athens; Aretaieion Hospital); D Kelgiorgi (Athens; Athens Euroclinic); C Chouliaras (Athens; Athens Medical Center); E Fradelos (Athens; Athens Naval and Veterans Hospital); N Michalopoulos (Athens; Attikon University General Hospital); N Dimitrokallis (Athens; Evaggelismos General Hospital); A Paspala (Athens; Evgenideio Hospital); P Christodoulou (Athens; General Hospital Asklepieio Voulas); EC Tampaki (Athens; KAT Athens General Hospital); D Schizas (Athens; Laiko University Hospital); K Kontzoglou (Athens; National and Kapodistrian University of Athens); M Spartalis (Athens; Sotiria General Hospital of Thoracic Diseases); M Billis (Filiates; General Hospital of Filiates); N Tsakiridis (Florina; Florina General Hospital ‘Eleni Th. Dimitriou’); A Karakosta (Ioannina; University Hospital of Ioannina); D Panagopoulos (Kyparissia; General Hospital of Messinia, Hospital Unit of Kyparissia); G Koukoulis (Larissa; General Hospital of Larissa ‘Koutlimpaneio and Triantafylleio’); G Christodoulidis (Larrisa; General University Hospital of Larissa); K Bouchagier (Patras; General University Hospital of Patras); V Mousafeiris (Patras; Karamandaneio Prefecture Children Hospital of Patras); A Papadopoulos (Piraeus; General Hospital of Nikaia); L Katsiaras (Piraeus; Metaxa Cancer Hospital); N Zampitis (Piraeus; Tzaneio General Hospital); O Ioannidis (Thessaloniki; George Papanikolaou General Hospital of Thessaloniki); M Drogouti 5 (Thessaloniki; O Agios Dimitrios General Hospital); C Kaselas (Thessaloniki; Papageorgiou General Hospital); D Lytras (Volos; Achillopoyleio General Hospital of Volos). Guatemala: M Aguilera-Arevalo (Guatemala City; Hospital General San Juan De Dios); L TaléRosales (Guatemala City; Hospital Juan Jose Arevalo Bermejo); ST Torres Rodríguez (Guatemala City; Hospital de Referencia Nacional de Enfermedades Respiratorias). India: N Krishnappa (Bangalore, Karnataka; BGS Global Institute of Medical Sciences); S Kumar Venkatappa (Bangalore; Victoria Hospital); TS Mishra (Bhubaneswar; All India Institute Of Medical Sciences - Bhubaneswar); Y Sakaray (Chandigarh; Postgraduate Institute of Medical Education & Research, Chandigarh, India); R Kottayasamy Seenivasagam (Coimbatore; PSG Institute of Medical Sciences and Research); R Sharma (DELHI; St Stephen’s Hospital); R Gupta (Dehradun; Synergy Institute of Medical Sciences); M Luthra (Delhi; Holy Family Hospital); T Longkumer (Dimapur; Christian Institute of Health Sciences and Research); A Chhabra (Faridkot; Guru Gobind Singh Medical College & Hospital (Baba Farid University of Health Sciences)); T Doma Bhutia (Gangtok; Sir Thutob Namgyal Memorial Hospital Sochakgang); M Pathak (Jodhpur; All India Institute of Medical Sciences (AIIMS), Jodhpur); J Rathod (Karamsad; Shree Krishna Hospital); MK Agrawal (Lucknow; King George’s Medical University); D Jain (Ludhiana; Christian Medical College & Hospital); NK Chaudhry (Ludhiana; Satguru Partap Singh Hospital); A Mathew (Madhepura; Madhepura Christian Hospital); P Alexander (Manali; Lady Willingdon Hospital); V Kumar (Manipal; Kasturba Medical College Hospital, Manipal); RD Sharma (Mumbai; Lilavati Hospital & Research Centre); B Sarang (Mumbai; Terna Medical College and Hospital); D Singh (Nandurbar; Chinchpada Christian Hospital); N Gupta (New Delhi; ABVIMS Dr RML Hospital); S Kulkarni (New Delhi; Army Hospital Research & Referral New Delhi); T Rashid (New Delhi; Hamdard Institute of Medical Sciences & Research); L Bains (New Delhi; Maulana Azad Medical College); T Iahmo (Padhar; Padhar Hospital); A Kumar (Patiala; Government Medical College Patiala); M Kumar (Patna; All India Institute of Medical Sciences, Patna); R Abhinaya (Pondicherry; Jawaharlal Institute of Postgraduate Medical Education and Research); VS Jha (Pune; Command Hospital, Southern Command); D Dugar (Raipur; All India Institute of Medical Sciences Raipur); S Basu (Rishikesh; All India Institute Of Medical Sciences); K Singh (SAS Nagar (Mohali) ; BR Ambedkar State Institute of Medical Sciences Mohali); C Mahakalkar (Sawangi (Meghe), Wardha; Acharya Vinoba Bhave Rural Hospital); FQ Parray (Srinagar; Sher-iKashmir Institute of Medical Sciences); JA Kalyanapu (Tezpur; Baptist Christian Hospital); M Chisthi (Thiruvananthapuram; Government Medical College Thiruvananthapuram); A Kavalakat (Thrissur; Jubilee Mission Medical College & Research Institute); B Roopavathana. S (Vellore; Christian Medical College & Hospital). Iran, Islamic Rep.: N Yousefzadeh Kandevani (Bastak; Farabi hospital); M Pourfridoni (Jiroft; Imam Khomeini Hospital). Iraq: R Raheem Attallah Al_obaidy (Anbar; Heet General hospital); Z Alkhuzaie (Najaf; Al Batool private hospital); MA Al-Juaifari (Najaf; Al-Najaf Al-Ashraf Teaching Hospital). Ireland: S Ramjit (Dublin; St James’s Hospital); R Tummon (Kerry; University Hospital Kerry). Israel: S Abu Salem (Jerusalem; Hadassah Medical Center). 6 Italy: R Sulce (Arezzo; Ospedale San Donato USL Toscana Sud Est); PM Cicerchia (Ariccia; Ospedale dei Castelli (N.O.C.)); E Marra (Aversa; San Giuseppe Moscati); M Rottoli (Bologna; IRCCS Azienda Ospedaliero-Universitaria di Bologna); J Andreuccetti (Brescia; ASST Spedali Civili, Ospedale di Brescia); E Locci (Cagliari; Cagliari University Hospital); F Cappellacci (Cagliari; Chirurgia Generale e Polispecialistica, Cagliari University Ospital ‘Duilio Casula’); N Cillara (Cagliari; Santissima Trinità - ATS Sardegna); E Abate (Carate Brianza (MB); Ospedale Vittorio Emanuele III - Carate Brianza); F Ascari (Carpi; Ramazzini); S Romano (Casarano; Francesco Ferrari Hospital); M Veroux (Catania; Azienda Ospedaliero- Universitaria Policlinico San Marco); B Nardo (Cosenza; Azienda Ospedaliera di Cosenza); D Sasia (Cuneo; Santa Croce e Carle Hospital, Cuneo); G Baronio (Esine; ASST Valcamonica Ospedale di Esine); N Fabbri (Ferrara; Azienda Unità Sanitaria Locale di Ferrara); J Martellucci (Firenze; Azienda Ospedaliera Universitaria Careggi); G Canonico (Firenze; Ospedale San Giovanni di Dio); V Lizzi (Foggia; Ospedali Riuniti Azienda Ospedaliera Universitaria); F D’acapito (Forlì; Morgagni-Pierantoni); D Merlini (Garbagnate Milanese; ASST Rhodense - Ospedale di Garbagnate Milanese); A Barberis (Genoa; E.O. Ospedali Galliera); MF Amisano (Genoa; IRCCS Ospedale Policlinico San Martino); A Luzzi (Genoa; Ospedale Villa Scassi); F Palmieri (Gravedona ed Uniti; Ospedale Moriggia Pelascini); CL Bertoglio (Magenta; Ospedale ‘G.Fornaroli’, ASST-OVEST Milanese); E Baldini (Melzo; Ospedale Santa Maria delle Stelle, ASST Melegnano Martesana); M Ceolin (Milan; Humanitas Research Hospital); P De Nardi (Milan; IRCCS San Raffaele Scientific Institute, Milan); MG Piacentini (Milan; Ospedale Fatebenefratelli e Oftalmico); F Ferrara (Milan; San Carlo Borromeo); F Brucchi (Milan; Sesto San Giovanni Hospital); F Di Marco (Modica; Ospedale Maggiore); N Tamini (Monza; Fondazione IRCCS San Gerardo dei Tintori Monza, Scuola di Medicina e Chirurgia, Università Milano Bicocca); P Anoldo (Naples; Federico II University of Naples); R Patrone (Naples; Istituto Nazionale Tumori Fondazione, Pascale IRCCS); F Selvaggi (Naples; Primo Policlinico di Napoli); G Bellio (Padova; Piove di Sacco Hospital); P Venturelli (Palermo; Policlinico Universitario Paolo Giaccone); L Conti (Piacenza; G. Da Saliceto); L Morelli (Pisa; Azienda Ospedaliero Universitaria Pisana); SMM Basso (Pordenone; Azienda Sanitaria Friuli Occidentale (AS FO)); F Biolchini (Reggio Emilia; Azienda Unità Sanitaria Locale - IRCCS di Reggio Emilia); C Marafante (Rivoli; Ospedale degli Infermi di Rivoli); A Antinori (Rome; Fondazione Policlinico Universitario Agostino Gemelli); M Campanelli (Rome; Policlinico Tor Vergata Hospital, Rome); P Lapolla (Rome; Policlinico Umberto I Sapienza University of Rome); G Palomba (Salerno; San Giovanni di Dio e Ruggi d’Aragona); L Cardinali (San Benedetto del Tronto; Madonna del Soccorso Hospital); E Andolfi (Sansepolcro; Valtiberina); L Verre (Siena; Azienda Ospedaliero Universitaria Senese); G Poillucci (Spoleto (PG); San Matteo degli Infermi); E Pontecorvi (Sulmona; SS Annunziata); S Novello (Treviso; Ospedale Ca’ Foncello - Università di Padova (DISCOG)); M Santarelli (Turin; Città della Salute e della Scienza); L Cobellis (Vallo Della Lucania; Casa di Cura Prof. Dott. Luigi Cobellis); G Ietto (Varese Lombardy; University of Insubria, Ospedale di Circolo e Fondazione Macchi (Varese)); F Pederiva (Varese; Filippo Del Ponte Hospital, University of Insubria); A Iacomino (Venezia; Ospedale Civile - Santi Giovanni e Paolo); D Verdi (Venice; Mirano Hospital); A Broglia (Voghera; Ospedale Civile di Voghera); P Cianci (andria; Lorenzo Bonomo); M Angelucci 7 (rome; policlinico universitario campus bio medico of rome); G Calini (udine; santa maria della misericordia). Japan: H Yonekura (Aichi; Fujita Health University Bantane Hospital). Jordan: S Alananzeh (Ajloun; Al Iman Hospital); A Qasem (Amman; Al-Basheer Hospital); Y Alawneh (Amman; Ibn Al Haitham Hospital); S Al-Tahayneh (Amman; Islamic Hospital); A Khamees (Amman; Jordan University Hospital); MEH Albanna (Amman; Marka Specialty Hospital); RKZ Almahadin (Amman; Prince Hamza hospital); M Mahafdah (Ar Ramtha; King Abdullah University Hospital/ Jordan University of Science and Technology); O Mansour (As-Salt; Al Hussain New Salt Hospital); M Mubarak (Irbid; Ar Ramtha Govermental Hospital); A Alrababah (Irbid; Princess Basma Hospital). Kazakhstan: M Kulimbet (Almaty; City Clinical Hospital No.7, Asfendiyarov Kazakh National Medical University); I Fakhradiyev (Almaty; JSC ‘Central Clinical Hospital’, Asfendiyarov Kazakh National Medical University). Kenya: R Parker (Bomet; Tenwek Hospital). Lebanon: D Rahme (Beirut; Hopital Libanais Geitaoui); H Hamdar (Jbail ; Maritime Hospital). Libya: W Ebrahim (Albayda; Albayda Medical Center); M Saleh (Benghazi; Al-jalaa Teaching/Trauma Hospital); S Alsaeiti (Benghazi; Benghazi Children’s Hospital); R Michael (Benghazi; Benghazi Medical Center); A Bojazyah (Darna; Al-Wahda Hospital); H Bileid Bakeer (Gharyan; Gharyan Central Hospital); M Abudabbous, N Albahloul (Misurata; Misurata Central Hospital); A Egdeer (Nalut; Nalut Central Hospital); H Embarek (Sebha; Al-Majd Clinic); M Abdelkabir (Sebha; Aseel Alghad Clinic); H Idheiraj (Sebha; Sabha Medical Center); R Salim (Tobruk; Tobruk Medical Center); K Ayad (Tripoli; Alkhalil hospital); A Alragheai (Tripoli; Metiga Hospital); S Egreara (Tripoli; Sabratha teaching hospital); S Timmalah (Tripoli; Tripoli Medical Center/ Tripoli University Hospital); N Lahmer (Zawia; Zawia Teaching Hospital); N Ben Hasan (Zliten; Zliten Teaching Hospital). Lithuania: D Venskutonis (Kaunas; LUHS Kaunas Hospital); A Dauksa (Kaunas; Lithuanian University of Health Sciences Kaunas Clinics); A Gulla (Vilnius; Vilnius University Hospital). Madagascar: F Rasoaherinomenjanahary (Antananarivo; Joseph Ravoahangy Andrianavalona Hospital). Malaysia: MS Mohd Shah (Kelantan; Hospital Universiti Sains Malaysia); R Noor (Kota Bharu; Hospital Raja Perempuan Zainab II); SN Loke (Kuching, Sarawak; Sarawak General Hospital); MA Yunus (Malacca; Hospital Jasin); H Amin-Tai (Serdang; Hospital Pengajar Universiti Putra Malaysia (HPUPM)). Mali: KS Dembele (Ségou; District Hospital of Tominian). Malta: J Psaila (Msida; Mater Dei Hospital). Mexico: CM Nuño-Guzmán (Guadalajara; Antiguo Hospital Civil de Guadalajara); LA Flores Chávez (Guadalajara; Clínica de Especialidades más Centro de Cirugía Simplificada); CM Nuño-Guzmán, G Yanowsky-Reyes (Guadalajara; Hospital Civil Fray Antonio Alcalde); A Gonzalez Ojeda (Guadalajara; Hospital de Especialidades, CMNO-IMSS); G Ambriz González (Guadalajara; UMAE Hospital de Pediatria Centro Medico Nacional de Occidentes IMSS); EE Lozada Hernandez (León; Hospital Regional e Alta Especialidad del Bajio); M Trejo-Avila (Mexico City; Hospital General Dr. Manuel Gea González); C Moreno-Licea (Mexico City; Instituto Nacional de Ciencias Médicas y Nutrición 8 ‘Salvador Zubirán’); A Navarrete-Peón (Pachuca; Sociedad Española de Beneficencia); M Noguez Castillo (Querétaro; Hospital de especialidades del niño y la mujer); A Ramos-De la Medina (Veracruz; Hospital Español Veracruz). Morocco: I Gouazar (Marrakech; Centre Hospitalier Universitaire Mohammed VI, Marrakech); A Ouachhou (Rabat; Centre Hospitalier Universitaire Ibn Sina Rabat). Namibia: PR Nashidengo (Windhoek; Windhoek Central Academic Hospital). New Zealand: S Rennie (Masterton; Wairarapa Hospital); M Haimona (Wellington; Wellington Regional Hospital); F Fadzlullah (Whanganui; Whanganui Hospital); L Paterson (Whangarei; Whangarei Hospital). Nigeria: A Adeyeye (Ado Ekiti; Afe Babalola University Multi-System Hospital); J Olaogun (Ado-Ekiti; Ekiti State University Teaching Hospital); N Oloko (Bauchi; Abubakar Tafawa Balewa University Teaching Hospital Bauchi); P Agbonrofo (Benin City; University of Benin Teaching Hospital); A Abiodun (Bida; Federal Medical Centre Bida); U Ezomike (Enugu; University of Nigeria Teaching Hospital); SA Sani (Gwagwalada; University of Abuja Teaching Hospital); TA Lawal (Ibadan; University College Hospital); AO Lawal (Idi Araba; Lagos University Teaching Hospital); AI Okunlola (Ido Ekiti; Federal Teaching Hospital, Ido Ekiti); OM Williams (Ikeja; Lagos State University Teaching Hospital); A Adisa (Ile-Ife; Obafemi Awolowo University Teaching Hospitals Complex); T Mohammed (Ilesa; Obafemi Awolowo University Teaching Hospitals Complex Wesley Guild Hospital Unit); P Elemile (Ilishan-Remo; Babcock University Teaching Hospital); II Aremu (Ilorin; General Hospital); L Abdur-Rahman (Ilorin; University of Ilorin Teaching Hospital); JG Makama (Kaduna; Barau Dikko Teaching Hospital); IU Garzali (Kano; Aminu Kano Teaching Hospital); TT Ibiyeye (Lokoja; Federal Teaching Hospital Lokoja); OH Ekwunife (Nnewi; Nnamdi Azikiwe University Teaching Hospital); OH Ekwunife (Onitsha; Holy Rosary Specialist Hospital); O Ojewuyi (Osogbo; UNIOSUN Teaching Hospital); I Ogundele (Sagamu; Olabisi Onabanjo University Teaching Hospital); M Daniyan (Zaria; Ahmadu Bello University Teaching Hospital). North Macedonia: T Risteski (Skopje; University Clinic for Pediatric Surgery). Oman: H Al-Aamri (Ibra; Ibra Hospital); AH ALSharqi (Muscat; Sultan Qaboos University Hospital); M Al Hinai (Nizwa; Nizwa Hospital). Pakistan: S Ahmed (Islamabad; Dr Akbar Niazi Teaching Hospital); SH Waqar (Islamabad; The Pakistan Institute of Medical Sciences); M Shahid (Karachi; PAF Faisal Hospital); F Ashraf (Karachi; Patel Hospital); AN Syed (Karachi; The Indus Hospital); AS Ammar (Lahore; Bahria International Hospital, Bahria Orchard); K Hayat (Lahore; Services Hospital Lahore); N Talat (Lahore; The Children’s Hospital & The Institute of Child Health Lahore); W Mabood (Peshawar; Mercy Teaching Hospital); HW Bhatti (Rawalpindi; Benazir Bhutto Hospital); M Usman Malik (Sargodha; District Headquarter & Teaching Hospital - Sargodha). Palestine: B Mohamad (Bethlehem, West Bank; Beit Jala Governmental Hospital (Al Hussein)); A Alwali (Gaza; Al-Shifa Hospital); A AbuNemer (Gaza; Nasser Hospital); S Alijla (Gaza; Palestine Red Crescent Society - Al-amal Hospital); H Ayesh (Hebron, West Bank; Al-Ahli Hospital); H Abu-Arish (Hebron, West Bank; Governmental Hebron Hospital-Alia); D Rabaia, A Jaber (Jenin, West Bank; The 9 Martyr Dr. Khalil Sulaiman Hospital (Jenin Governmental Hospital)); M MohammedAli (Nablus, West Bank; Rafidia Hospital); A Attili (Tulkarm, West Bank; Martyr Thabet Thabet Govermental Hospital). Paraguay: OM Cuenca Torres (Asuncion; Hospital de Clínicas, II Cátedra de Clínica Quirúrgica, Universidad Nacional de Asunción). Peru: V Panduro-Correa (Huánuco; Hospital Regional Hermilio Valdizán Medrano); L Fuentes Rivera Lau (Lima; British American Hospital); CF Huaroto Landeo (Lima; Clinica Internacional); G Mendiola (Lima; Hospital Santa Rosa de Lima); Y Carpio Colmenares (Lima; SANNA - Clínica El Golf); C Shiraishi Zapata (Paita; Hospital I Miguel Cruzado Vera EsSalud); R Díaz-Ruiz (Piura; Jose Cayetano Heredia III Regional Hospital). Poland: Ł Nawacki (Kielce; Wojewódzki Szpital Zespolony w Kielcach); M Kisielewski (Krakow; 5th Military Clinical Hospital); Z Orzeszko (Krakow; Brothers Hospitallers Hospital); M Matyja (Krakow; Jagiellonian University Medical College); W Krawczyk (Sosnowiec; Wojewódzki Szpiital Specjalistyczny nr 5 im. Św Barbary); M Walędziak (Warsaw; Military Institute Of Medicine); JK Zajac (Wroclaw; Regional Specialist Hospital in Wroclaw); F Brzeszczyński (Łódź; Copernicus Memorial Hospital). Portugal: S Henriques (Almada; Hospital Garcia de Orta); J Frazão (Amadora; Hospital Prof. Doutor Fernando Fonseca, E.P.E.); S Gaspar Reis (Barreiro; Centro Hospitalar Barreiro Montijo, EPE); AR Mateus Loureiro (Caldas da Rainha; Hospital das Caldas da Rainha - Centro Hospitalar do Oeste, E.P.E); M Reia (Elvas; Hospital Santa Luzia Elvas); J Pinho (Figueira da Foz; Hospital Distrital da Figueira da Foz); DG Alves (Funchal; Hospital Dr. Nélio Mendonça); R Silva Borges (Horta; Hospital da Horta, E.P.E.); E Borges (Lisbon; Centro Hospitalar Lisboa Norte); M Nunes (Matosinhos; Unidade Local de Saude de Matosinhos - Hospital Pedro Hispano); A Faustino (Ponta Delgada; Hospital do Divino Espírito Santo); G Fialho (Portalegre; Hospital Doutor José Maria Grande); J Dias-Ferreira (Porto; Centro Hospitalar e Universitário de São João); M Santos (Porto; Hospital da Prelada); J Marques Antunes (Santa Maria da Feira; Centro Hospitalar Entre o Douro e Vouga); H Devesa (Santarem; Hospital de Santarem); J Ricardo (Santiago do Cacém; Hospital do Litoral Alentejano); R Branquinho (Tomar; Centro Hospitalar Médio Tejo); J Fernandes (Vila Franca de Xira; Hospital Vila Franca de Xira); J Pereira-Macedo (Vila Nova de Famalicao; Centro Hospitalar do Medio Ave); B Vieira (Vila Real; Centro Hospitalar de Trás-os-Montes e Alto Douro, E.P.E.). Qatar: CE Guldogan (Doha; Turkish Hospital). Romania: ST Makkai-Popa (Brasov; Regina Maria); F Grama (Bucharest; Coltea Clinical Hospital); EA Toma (Bucharest; Elias Emergency Hospital); I Negoi (Bucharest; Emergency Clinical Hospital Bucharest); M Muresan (Cluj-Napoca; Medicover Hospital Cluj). Russian Federation: V Kakotkin (Kaliningrad; Immanuel Kant Baltic Federal University, Regional Clinical Hospital); A Bedzhanyan (Moscow; Petrovsky National Research Centre of Surgery); S Katorkin (Samara; Hospital Surgery Clinic of Samara State Medical University); A Butyrskii (Simferopol; Municipal Emegency Hospital No.6); V Ten (Yuzhno-Sakhalinsk; Private healthcare institution ‘RZD-Medicine’). Rwanda: N Christian (Huye, Gisagara; Butare university teaching hospital (CHUB)); C Mpirimbanyi (Kigali; Kibagabaga Hospital); A Costas-Chavarri (Kigali; Rwanda Military Hospital). 10 Saudi Arabia: N Alzerwi (Al-Majmaah; King Khalid General Hospital); A Shabkah (Jeddah; International Medical Center); A Nawawi (Jeddah; King Abdulaziz University Hospital); N Alzerwi (Riyadh; King Salman Hospital in Riyadh); S Chowdhury (Riyadh; King Saud Medical City); DY Alalawi (Tabuk; King Salman Armed Forces Hospital); S Awad (Taif City; King Faisal Medical Complex). Serbia: A Karamarkovic (Belgrade; Zvezdara University Medical Center). Slovenia: JA Košir (Ljubljana; University Medical Centre). South Africa: M Flint (Cape Town; Groote Schuur Hospital); A Victor (Cape Town; Karl Bremer Hospital); SS Verhage (Cape Town; Khayelitsha District Hospital); F Gool (Cape Town; Mitchell’s Plain District Hospital); T Mabogoane (Cape Town; Victoria Hospital Wynberg); B Phakathi (Durban; King Edward VIII Hospital); V Pillay (Durban; Stanger Hospital); R Jayakrishnan (East London; Cecilia Makiwane Hospital); Y Manickchund (East London; Frere Hospital); O Jolayemi (Empangeni; Ngwelezana Hospital); H Stark (George; George Hospital); C Molewa (Johannesburg; Edenvale); H Wain (Pietermaritzburg; Edendale Hospital); D Montwedi (Pretoria; Kalafong Academic Hospital); G De Wee (Upington; Dr Harry Surtie Hospital); C Dempers (Worcester; Worcester Provincial Hospital). Spain: H Aguado López (Albacete; Hellín Hospital); MDM Martí-Ejarque (BARCELONA; Hospital Universitari Sagrat Cor); A Torroella (Barcelona; HM Nou Delfos); O Martin Sole (Barcelona; Hospital Sant Joan de Deu); A Landaluce-Olavarria (Bizkaia; Hospital Urduliz); V Alonso (Burgos; Hospital Universitario de Burgos); Á Fernández Camuñas (Ciudad Real; Hospital General Universitario de Ciudad Real); M Estaire Gómez (Leganés; Severo Ochoa University Hospital); AG Barranquero (Lleida; Hospital Universitari Arnau de Vilanova); L Marquez (Madrid; Hospital Central de la Cruz Roja San Jose y Santa Adela); P Serrano Méndez (Madrid; Hospital Clinico San Carlos); A Vilar (Madrid; Hospital Universitario Principe de Asturias); J Guevara (Madrid; Hospital Universitario la Paz); AM Minaya Bravo (Madrid; Hospital del Henares); JL Rodicio Miravalles (Oviedo; Hospital Universitario Central de Asturias (HUCA)); R Díaz Pedrero (Rivas-Vaciamadrid, Madrid; Hospital Universitario HM Rivas); L Tallon-Aguilar (Sevilla; Hospital Universitario Virgen del Rocio); A Curado Soriano (Seville; Hospital Universitario Virgen Macarena); Z Balciscueta (Valencia; Hospital Arnau de Vilanova); D Moro-Valdezate (Valencia; Hospital Clínico Universitario de Valencia); B De Andrés-Asenjo (Valladolid; Hospital Clínico Universitario de Valladolid); A Vazquez Melero (Vitoria-Gasteiz; Hospital Universitario Araba); J Escartin (Zaragoza; Hospital Royo Villanova); C Gracia-Roche (Zaragoza; Hospital Universitario Miguel Servet). Sri Lanka: S Srishankar (Anuradhapura; Teaching Hospital Anuradhapura); D Wickramasinghe (Colombo; National Hospital of Sri Lanka); U Jayarajah (Dehiwala; Colombo South Teaching Hospital); S Gobishangar (Jaffna; Teaching Hospital, Jaffna); W Wijenayake (Werahera; University Hospital, Kotelawala Defence University). Sudan: I Abdalla (Ed Dueim; Ed Dueim Teaching Hospital); S Ibrahim Tour Harakan (El Geneina; El Geneina teaching Hospital); MM Yassin (Gadarif city; Gadarif teaching hospital); Z Aljalabi (Kassala; Police hospital); I Adel (Khartoum; Bashair Teaching Hospital); IMG Ahmed (Khartoum; Ibrahim Malik Teaching Hospital); M Hajalamin (Khartoum; Omdurman Teaching Hospital); EE Abuobaida Banaga 11 Hag El Tayeb (Khartoum; Ribat university hospital); HA Fadlalmola (Khartoum; Soba University Hospital); E Alkhalifa (Wad Madani; University of Gezira Hospital). Sweden: H Zaigham (Malmö; Skåne University Hospital); M Nikberg (Vasteras; Västmanlands Hospital Västerås). Switzerland: P Probst (Frauenfeld; Spital Thurgau AG); E Gialamas (Geneva; Geneva University Hospitals); J Gass (Luzern; Luzerner Kantonsspital); A Tampakis (Olten; Kantonsspital Olten); G Peros (Winterthur; Kantonsspital Winterthur); MA Schneider (Zurich; University Hospital of Zurich). Syrian Arab Republic: MA Farho (Aleppo; Abd Al Wahab Agha Hospital); AA Kayali (Aleppo; AlShahbaa Private Hospital); M Aloulou (Aleppo; Aleppo Private Hospital); A Ghazal (Aleppo; Aleppo University Hospital); B Alsaid (Damascus; Al assad university hospital); M Klib (Damascus; AlMouwasat University Hospital); H Dalati (Damascus; Children’s University Hospital); S Jomaa (Damascus; Damascus Hospital); Y Alhammoud (Homs; Al-Basel Specialized Hospital in Karm ElLouz); S Abbas (Homs; The Military Hospital); G Hneino (Latakia; Al Saydeh Surgical Hospital); I Ali (Latakia; National Hospital); G Bashour (Latakia; Othaman Hospital); A Hammed (Latakia; Tishreen University Hospital). Thailand: S Techapongsatorn (Bangkok; Vajira hospital). Togo: F Alassani (Lomé; CHU Sylvanus Olympio). Tunisia: A Sebai (Tunis; La Rabta Hospital). Turkey: GC Bulbuloglu (Adana; Adana Seyhan State Hospital); MA Koç (Ankara; Ankara University Medical School); MY Uzunoglu (Bursa; Bursa City Hospital); B Yigit (Elazig; Elazig Fethi Sekin City Hospital); AN Sanli (Gaziantep; Abdulkadir Yuksel State Hospital); GK Kurtoglu (Istanbul; Acibadem Altunizade Hospital); E Tuzuner (Istanbul; Acibadem Maslak Hospital); Y Altinel (Istanbul; Bagcilar Research And Training Hospital); ÖP Zanbak Mutlu (Istanbul; Bahçelievler State Hospital); RE Sönmez (Istanbul; Istanbul Medeniyet University, School of Medicine); S Bektas (Istanbul; Istanbul Medipol University Hospital); E Erginöz (Istanbul; Istanbul universty - Cerrahpaşa Medical faculty); A Özcan (Istanbul; Kanuni Sultan Suleyman Training and Research Hospital); Y Tosun (Istanbul; Kartal Dr. Lutfi Kirdar Training and Research Hospital); İH Özata (Istanbul; Koç University Medical School); TK Uprak (Istanbul; Marmara University, School of Medicine); E Unal (Istanbul; Sehit Prof.Dr. İlhan Varank Training and Research Hospital); N Kiziltoprak (Istanbul; Sultan 2. Abdulhamid Han Training and Research Hospital, University of Health Sciences); M Ergenç (Istanbul; Sultanbeyli State Hospital); MT Demirpolat (Istanbul; University of Health Science Umraniye Education and Research Hospital); B Citgez (Istanbul; Uskudar University Faculty of Medicine, Memorial Hospital); H Ulman (Izmir; Bakircay University Cigli Training and Research Hospital); YK Şen (Izmir; University of Health Sciences Izmir Bozyaka Training and Research Hospital); E Colak (Samsun; Samsun University Samsun Training and Research Hospital); N Kavak (Zonguldak; Zonguldak Bulent Ecevit University School of Medicine Research and Training Hospital); E Kamer (İzmir; University of Health Sciences Tepecik Training and Research Hospital). Uganda: I Mubezi (Iganga; Iganga district hospital); H Lule (Kigumba; Kiryandongo Hospital); S Stonelake (Luwero; Kiwoko Hospital). 12 United Kingdom: CS Ong (Bangor, North Wales; Ysbyty Gwynedd); P Patel (Barrow in Furness; Furness General Hospital); S Dindyal (Basildon; Basildon University Hospital); F Georgiades (Bedford; Bedford Hospital); J Abbasy (Birmingham; Heartlands Hospital); M Kaur (Brighton; Royal Sussex County Hospital); B Martin (Bristol; Bristol Royal Hospital for Children); M Chauhan (Camberley; Frimley Health NHS FT - Frimley Park); S Ahmed (Chester; Countess of Chester Hospital); M Tutton (Colchester; Colchester Hospital University); N Chidumije (Coventry; University Hospitals Coventry and Warwickshire NHS Trust); W Al-Khyatt (Derby; Royal Derby Hospital); A Sukumar (Dudley; Russell’s Hall Hospital); H Kamal (Dundee; Ninewells Hospital); A Nada (Durham; University Hospital North Durham); A Chaudhary (Exeter; Royal Devon and Exeter Hospital); M Bogdan (Great Yarmouth; James Paget Univeristy NHS Foundation Trust Hospital); M Peter (Huddersfield; Huddersfield Royal Infirmary); J Walshaw (Hull; Hull University Teaching Hospitals NHS Trust); M Ewedah (Ilford; King George Hospital); L Rampersad (Larbert; Forth Valley Royal Hospital); A Peckham-Cooper, NS Blencowe (Leeds; Leeds Teaching Hospitals); R Lunevicius (Liverpool; Aintree University Hospital); P Panahi (London; Ealing Hospital); E Baili (London; Guy’s and St Thomas’ Hospitals); K Theodoropoulou (London; Homerton University Hospital); P Kapsampelis (London; Kingston); MMH Mohammed (London; Queen Elizabeth Hospital, Woolwich); C Parmar (London; The Whittington Hospital); MMH Mohammed (London; University Hospital Lewisham); C Smart (Macclesfield; Macclesfield District General Hospital); P Wilson (Manchester; Wythenshawe Hospital); F Gareb (Margate; Queen Elizabeth the Queen Mother Hospital Margate); G Sundaram Venkatesan (Middlesbrough; James Cook University Hospital); C Hidalgo Salinas (Morecambe; Royal Lancaster Infirmary); S Tingle (North Shields; Northumbria NHS Hospital Trust); N Marzouqa (Nottingham; Queens Medical Centre); T Theivendrampillai (Oxford; John Radcliffe Hospital); M Zahed Abdalla (Portsmouth; Queen Alexandra Hospital); A Rahman (Redhill; East Surrey Hospital); M Abdelkarim (Rhyl; Glan Clwyd Hospital); O Whitehurst (Salford; Salford Royal Hospital); E Tokidis (Sheffield; Sheffield Teaching Hospital NHS Foundation Trust); S Bandyopadhyay (Southampton; Southampton General Hospital); B Al-Sarireh (Swansea; Morriston Hospital Swansea); A Salam (Walsall; Walsall Manor Hospital). United States: M Sulciner (Boston; Brigham and Women’s Hospital); G Chang (Chicago, IL; Mount Sinai Hospital); A Alecci (Chicago, IL; Rush University Medical Centre); HE Rice (Durham, NC; Duke University Medical Center); D Ridder (Honolulu, Hawaii; The Queen’s Medical Center); K McKenzie (Jamaica; Jamaica Hospital); A Choudhry (Syracuse; SUNY Upstate University Hospital). Yemen, Rep.: MY Abdualqader (Hajjah; Kowaydina hospital); B Alshaikh (Sana’a; Al-Thawra Modern General Hospital).

Collaborators (listed by country and city):

Albania: B Ibi, S Faber (Korca; Regional Hospital of Korca); I Dajti (Tirana; University hospital Koco Gliozheni). Algeria: K Bensmain, ZR Benamrouche , IE Boumakhlouf, IE Boudis, H Abdoun , M Benamrouche , M Saidani (Algiers; CHU Isaad Hassani); Z Djama, A Chied, HA Mimouni (Constantine; university 13 hospital abdelhamid ben badis); AK Awad, B Radja, B Abdennour , MN Bouhafs, MEA Meghaizerou (Oran; EHU-1st November 1954). Argentina: P Carmignani, J Mondino, R Figueroa, J Morales, FR Pascual (Allende, Cordoba; Sanatorio Allende - Sede Cerro); MA Bequis, F Suldrup, C Korzin, J Napoli , N Feijoo, F Mahnic, ME Duran, L Chantada, C Brandi, JF Viñas, F Lucero, C Samojeden, LJ Caram, S Bertone, F Corvatta (Buenos Aires; Hospital Italiano de Buenos Aires); L Garciandia (Buenos Aires; Hospital Universitario CEMIC); M Rius, S Matthiess, J Paredes, MC Kalaydjian , A Veira (Buenos Aires; Hospital municipal de vicente lópez); MA Fernández Zurita, JI Valenzuela (City of Buenos Aires; Hospital Velez Sarsfield); S Gomez, GR Viscido, MA Doniquian (Cordoba; Clinica Universitaria Reina Fabiola); R Badra, JS García, CI Ferrero, M Garcia, L Granero (Cordoba; Sanatorio Allende - Sede Nueva Cordoba); M Pagani (San Francisco; Clinica Regional del Este). Aruba: M Gosselink, J Ringers (Oranjestad; Dr. Horacio E Oduber Hospital). Australia: M Lie, B Mao, C Stennard, EMA Murphy, JE Do, M Harris, B Fosh (Adelaide; Northern Adelaide Local Health Network); M Watson, J Petric, M Maclean, XY Po, M Pham, D Patterson, V Gunasaegaram, E Hopping, P Holt, JA Duffield (Adelaide; Royal Adelaide Hospital); E Schmidt, R Colbran (Brisbane; Princess Alexandra Hospital); V Liu, E Tan, JST Tefay, R Shen, S Bowman (Brisbane; Queen Elizabeth 2 Jubilee Hospital); D Mitchell, M Kelly, A Edmundson (Brisbane; Surgical Treatment and Rehabilitation Service); H Iswariah, R Franz, M Chandrasegaram, P Yuide, SS Hlaing (Brisbane; The Prince Charles Hospital (TPCH)); S Abeykoon, D Kaushal (Campbelltown; Campbelltown Hospital); C Leung (Canberra; Calvary Hospital); S Davis, NF Franco (Canberra; Canberra Hospital); T Rawther, R McClen, W Petrushnko, E Roussos, K Das (Coffs Harbour NSW; Coffs Harbour Health Campus); S Stevens, F Alnimri, S McClintock, J Maritz (Colac; Colac Area Health); S Hariharan, S Laura, B Wang, V Ng, J Linker, A Li, I Dong, R Bhatia, S Cai, WKH Lai, AC Dawson, SYD Chia, M Binks, N Tran, SHM Ng, D Shen, EWY Lun, E Reid, J Cui, M Roussos (Gosford; Gosford Hospital); M Issa, M Anandan, P Devlin, U Naidoo (Hamilton; Hamilton Base Hospital); B Balaravi Pillai, D Abeysirigunawardana , T Parker, T Valizadeh Elizeh (Melbourne; Angliss Hospital); D Liu, S Ng, J Jones, O Ladlow, JV Maida, D Proud, A Vu, N Shulman, L Bromley, V Muralidharan, K Hall, C Cheong, C Jamieson-Grigg (Melbourne; Austin Hospital); A Hilder, T Manickam, L Barnard (Melbourne; Box Hill Hospital); K Jaffry, A Gray, A Lim, R Kattini (Melbourne; Casey Hospital); M Bickford, S Kenworthy (Melbourne; Knox Private Hospital); A Crowe, J Zhu (Melbourne; Maroondah Hospital); M Pacilli, A Comella, K Taghavi, R Nataraja, SJA Robinson (Melbourne; Monash Childrens Hospital); D Lowen, A Khan, S Samadi, S Tan, AL Surkitt, S Condron, E Haege, E Francis, A Boynes, S Gill, B D’Souza, H Xiao, E Fraser, J Wong, S Fennelly, K Mori, M Muir, Y Huang, R Pajtak (Melbourne; Northern Hospital); J You, C Banal, T Abelman, N Chen, L Chong, H Jalilehvand, W Santucci, B McKay (Melbourne; St Vincent’s Hospital); I Murshed, D Makary, L Green, M Wichmann, A Lim, M Kang, EA Dontoh (Mount Gambier; Mount Gambier and Districts Health Service); P Walker, A Fani, S Sundararajan, E Downes, A Davis, M Bajwa, R Geow, KK Sim (Mount Nasura; Armadale Health Service); S Smith, L Peters, S Zhang, ACK Cheung, I Caitens (Newcastle; Calvary Mater Newcastle); M Ishak, E Zhang, KKA Yu, L Beukes, L Kang, , F Amico (Newcastle; John Hunter Hospital); M Bowles, E Downing, B Williams, G Cox, V Bakshi 14 (Robina; Robina Hospital); N Ensor (Sale; Sale Hospital / Central Gippsland Health Service); J Ng, CT Petcu, J Hwang (Southport; Gold Coast University Hospital); TJ Hugh, S Quoy, C Knee, D Siriwardena, C Tung, G Smith (Sydney; Royal North Shore Hospital); O Camilleri, J Vu, J Hong, C Cornwell (Sydney; The Royal Prince Alfred Hospital); E Chan, C Killoran, J Mackenzie, D Fry (Toowoomba; Toowoomba Hospital); V Mahendravarman, A Shanmugalingam, C Li, L Allan, P Shivashankar, H Pleass (Westmead; Westmead Hospital); V Lin, R McGee, B Bereket Araya, CW Un, Y De Silva, D Maan, J Wang, M Kwon, R Kibuuka, J Chew, J Siu, J Barklimore (Wyong; Wyong Public Hospital). Austria: N Klammer, R Schmidt-Branden, P Tschann, P Horvath (Feldkirch; Landeskrankenhaus Feldkirch); N Koter, G Moitzi, E Wallner, C Allmer, F Aigner (Graz; Barmherzige Brüder Krankenhaus, Graz); J Kahn, A Belarmino, R Sucher, V Wolfschluckner, G Singer (Graz; Medical University of Graz); R Függer, M Biebl, A Punzengruber, H Fehrer (Linz; Ordensklinikum Linz Elisabethinen); A Binder, E Haiden, P Riedl, M Enßlin (Tulln; Universitätsklinikum Tulln). Bangladesh: K Nahar, T Akter (Dhaka; Dhaka Medical College Hospital); A Oosterkamp (Dinajpur; Lamb Hospital). Benin: G Gbessi, J Avakoudjo, M Fiogbe, P Assouto, SP Chigblo (Cotonou; Centre National Hospitalier et Universitaire Hubert Koutoukou Maga); H Aouagbe Behanzin , M Seto, G Mevognon (Cotonou; Hopital de Menontin); M Agbadebo, A Hada, SFA Houndji (Dassa-Zoumè; Hôpital de Zone de Dassa-Zoumè); E Bara (Kandi; Hôpital de zone de Kandi); AB Yevide, ZF Tamou, E Hatangimana, B Cakpo , R Soglonou (Klouékanme; Hopital de Zone de Klouékanme); TK Hessou, SR Tobome, M Zounon (Natitingou; Centre Hospitalier Départemental de l’Atacora); AM Hodonou, C Bokossa , F Hounde , R Alinde (Parakou; Centre Hospitalier Universitaire Borgou Alibori); F Dossou, R Goudou, ACS Toi , G Natchagande (Porto Novo; Centre Hospitalier Universitaire et Departemental Oueme Plateau). Bosnia and Herzegovina: M Stjepanovic (Doboj; Genera Hospital ‘Sveti aposto Luka’ Doboj); O Čančar, M Pejović (Foča; University Hospital Foča); J Miskovic, M Boras, M Kajic, V Dragisic, Z Brekalo, I Mikulic, N Soldo, M Bevanda, M Faletar (Mostar; SKB University Clinical Hospital Mostar); M Salibašić, E Hodžić, E Halilović, M Kruščica, E Bičakčić (Sarajevo; Clinical Center University of Sarajevo); A Cerovac, H Škiljo, E Hodžić, O Bedak, M Kalabić, E Begunić (Tešanj; General Hospital Tešanj); A Huremovic, E Alić (Tuzla; University Clinical Center Tuzla). Brazil: RA Tenfen Carneiro (Francisco Beltrão; Universidade Estadual do Oeste do Paraná). Bulgaria: D Georgiev, I Fidoshev, V Neykov, E Daleva, I Ilieva (Pleven; Heart and Brain - Pleven Hospital); M Karamanliev, D Dimitrov, A Shanker, P Vladova, MD Shoshkova, A Mehta, M Abdullahi, V Ratheesh, V Kamalathevan, C Wiesner, S Shittu, M Galasyuk, S Shanker (Pleven; University Hospital Dr Georgi Stranski, Medical University - Pleven); M Imirski, A Soumpasis (Plovdiv; MHAT St. Karidad); E Hadzhieva, D Chakarov (Plovdiv; UMHAT Sveti Georgi); T Yotsov, P Kamenova, A Vricheva, I Yotsov (Ruse; University Hospital Medika); E Hristova, K Spassov (Sofia; Fifth City Hospital Sofia - 5th MBAL). Burkina Faso: A Sanou, M Windsouri, R Doamba, IW Bahikoro , AST Sanon (Ouagadougou; Tengandogo University Hospital). 15 Burundi: N Ildephonse, N Steve , Y Fulgence , GD Nibogora, C Nimbona , B Paul (Bujumbura; Centre Hospitalo-Universitaire de Kamenge); G Kazobinka, C Rukundo, N Renovat, E Ndizeye , M Dauphin (Bujumbura; Kamenge Military Hospital); FF Irakiza, L Niyidukunda, G Nkunguzi, N Oscar, N Theophile, B Révérien (Bujumbura; Prince Regent Charles Hospital). Cambodia: S Oum, S Eam (Battambang; Handa Medical Centre). Cameroon: NS Bibila, NN Cabrel, J Dongmo (Bamenda; Nkwen Baptist Hospital). Canada: R Spence, G Berger, D Hannedige, C Hoogerboord, A Abidali (Halifax; Queen Elizabeth II); M Mozel, L Monteiro, R Lertnamvongwan, D Konkin, R Kaur (Port Moody; Eagle Ridge Hospital); M Mozel, R Lertnamvongwan, L Monteiro, S MacKenzie, D Konkin (Vancouver; Royal Columbian Hospital). Chile: MM Modolo, E Sepúlveda, Á Molero, M Perez, LV Torres Bavestrello, ÓA Soublett Rivas , C Carrillo Sarango, L Paredes, P Sornoza, A Pinto, C González (Santiago; Hospital Barros Luco Trudeau). China: J Wang (Guangzhou; The First Affiliated Hospital of Jinan University). Colombia: L García-Zambrano, PA Cabrera Rivera, N Paez, SV Agudelo Mendoza, MS Mosquera Paz, A Kadamani Abiyomaa, CF Roman Ortega, F Casas J, B Guerra (Bogota; Fundacion Cardioinfantil-IC); JD Molina Marin, C Maya, C Vasquez Maya, B Dieck, F Zapata (Medellin; Clínica CES); VA Ruiz López, MA Ñañez (Popayan; Hospital Susana Lopez de Valencia); DC Cardona Gomez, A Rojas, DC Patiño García , LI Bolaños, C Pastás, DA Pérez Muñoz (Popayán; Hospital Universitario San José). Croatia: J Mihanovic, I Bacic, D Vukosav , V Žufić, O Jurić, E Dijan, N Jović, I Ćoza, I Rakvin, Z Katusic, T Soric, I Vidić, D Rukavina (Zadar; Zadar General Hospital); I Separovic, R Radojković, J Mavrek (Zagreb; University Hospital Centre Zagreb). Cyprus: M Theodoridou, A Pilavas, R Moukarzel, R Andreou, N Gouvas, K Lambri, S Charitonos , IC Mylona, G Kokkinos, N Dimitriou, MM El Ghoul Miliotou, V Ioannou, NA Ververidis, N Kalampokis, A Yiallourou, R Sokratous, D Tsiardas , P Evangelou, D Evripidou, C Thrasyvoulou (Nicosia; Nicosia General Hospital). Czech Republic: T Reichelt, P Hudáč (Decin; Krajská zdravotní as - Hospital Decin); K Akter, L Moolla, F Rudisch, A Ibrahim Hassan, O Ahmad, E ELShennawy, M Shalaby, M Khaled, A Akiba, F Philips, E Bankart, R Elshennawy, A Al Kaddah, H Al Atassi, S Ashry, N Salgadoe (Hradec Kralove; Charles University Hospital); L Majerčák , P Levíček, A Lukáč, L Pánči (Ivančice; Hospital Ivančice); J Roman, L Tulinsky, I Mrazkova, P Ostruszka, A Varga, L Martinek (Ostrava; University Hospital Ostrava); H Novák, J Woleský, P Francúz (Prague; Motol University Hospital). Dominican Republic: R Rivas, B Calcaño, J Michel, Y Perez, R Ubiñas, P Garcia-Dubus, S Batista, S Strachan (Santo Domingo; CEDIMAT - Centro de Diagnóstico, Medicina Avanzada, Laboratorio y Telemedicina). Egypt: Y Tanas, Y Kerolous, Y El Okazy, M Mokhtar, M Lotfy, M AL Sayed, H Altabbaa, AGMM Abouelnagah, O Al Shaqran, D M. Awad, A Sabry, G Nagy, E Amer, M Khalil, A El Shamarka, B Sharaf eldin, AAA Aboshosha, A Farrag, H Sherif Farouk Ahmed Hassan, Y Badr (Alexandria; Alexandria Main University Hospital); Y Orabi, M Kamal matter, A Alrifaee (Alexandria; Alexandria 16 Medical Research Institute); M Elnour , M Zahran, A Aladl, M Bahnacy, Y Seada, M Kotb, A Ragab, Y Farag, L Khalifa, M Elmiesiry , D Abdalaziz, I Maharem, O AbouHiekal, O Hany, S Hanna, Y Dean, A Faisal (Alexandria; Smouha University Hospital); M Mostafa, I Ali, T Sabra, H Ibrahim , A K. Ali, M Osman , A Eltayeb (Assiut; Assiut University Children Hospital); A Morad, MO Herdan , A Abdelshafi, M M. Nathan, M Shalkamy, M Hamada Takrouney, R Sayad, FA Monib, A A Elhars, MM Saad, A Rashad Temerik, AM Abbas, O Mohamed Mokbel, E AbdElBaset, A Barakat, Z Bady, S Arafa, Z Osama, A Elzanaty , S Salama (Assiut; Assiut University Hospital); MEM Madany (Aswan; Aswan University Hospital, Aswan University); A Khaity , R Adel Diab, A Ghazal, A Ehab, A Abd Elsattar (Cairo; Al Zahraa University Hospital); A El-bastwesy (Cairo; Al-Azhar University Hospitals); A Eisa, M Elesseily, R Radwan, Y Asar, D Waleed, S Tawfik , AF Nixon Fulli , MJI Albert, A Autiak Ayii Chol, AR AbdelHalim, B Azhar, H Al-derume , M Alqadasi, N Alasbahi (Cairo; Cairo University Children’s Hospitals (CUSPH & CUCH)); H Abozied, Y Ashour, Y Mohamed , M Abdelmaboud, H Abdelazim, AES El kady, M Omar, A Haty, M Abd Al-Fattah, I Tagreda , IM Kereet, AG Montaser, M Faisal, M Masoud , M ElSayed Metwally (Cairo; EL-Hussein University Hospital, Al-Azhar University, Faculty Of Medicine); ASM Abdelrahman, S Mansour (Cairo; Giza International Hospital); A Nabil, MMA Marei, A Elmosalamy, L ElGebaly, AM Allam, T Awad, H Taher, K Fayed, M Abdelfattah, DH Khattab, N Ali, A Saleh, K Nassim, NK Aly, I Abo Elhagag, M Doss, M Elzayat, Y Samer Morsy, M ElFiky (Cairo; Kasr Al Ainy Faculty of Medicine, Cairo University); M Erfan, M Zaazou , M Reda, M Kouta, M Mohamad Amin, I Guirguis, H Amir , S Aboseif, M Abdelhafez, O Agha, A Khairy , A Dawoud, M Hamoud Almahly, S Mostafa Yassin , A El-Sherbiney, A Adel, H Foda, D Ahmed, AS Elkhodary , AA Mansour (Giza; The Memorial Soaad Kafafi University Hospital); A Elghrieb, M Natey, A Elshazli Mahmoud , A Khalleefah, H Elfeki, M Shalaby, M Sadek, M Abdelmaksoud, M Mostafa, M Waseem, A Adel, A Azam, A Sakr, A Sanad, عبدالفتاح م) Mansoura; Mansoura University Hospital); H Foad, S Elnoamany, S Selim, DS Alrokh, A Hassanin (Menofia; Menofia University Hospital); M Alansary (Qena; Qena University Hospital); A Ragheb, M Fahmy , M Mehanny (Sohag; Sohag University Hospital); AGE Aboelnasr, KMG Mohammed, M Eissa, S Allam, M Kamar (Tanta; Tanta University Hospital); A Asla (Zagazig; Al Ahrar Zagazig Teaching Hospital). Ethiopia: F Terefe, WA Zerefa, E Gallo , A Yingess, T Kebede, M Mesfin, G Alemayehu , SM Djote, T Girma, DA Muhie (Addis Ababa; Yekatit 12 hospital medical college); E Yeshialem, G Seyfu, F Tsige, A Yeshitila, N Solomon (Deberebirhan; Hakim Gizaw Hospital); M Worku, S Lakew, Y Melkamu, ST Workineh, M Beletachew (Dessie; Dessie Referral Hospital); B Mengesha, G Getachew , I Tesfahun, M Teressa , BG Chiman, AB Aregawi (Hawassa; Hawassa University Comprehensive Specialized Hospital); N S.Bayleyegn, YY Metaferia, TG Moges, D Mengiste, A Teshome Sahilemariam (Jimma; Jimma University Medical Center); B Sime, T Jemal (Yirgalem; Yirgalem Hospital Medical College). France: E Volpin, H Braham, C Lionel, R Arena, Y Malki (Eaubonne; Hôpital Simone Veil); M Bertrand, A Castaldi, L Theuil, A L’Hostis, M Prudhomme (Nimes; Hôpital Carèmeau); P Riva, A Lapergola, D Mutter, S Perretta (Strasbourg; Nouvel Hopital Civil de Strasbourg). Gabon: PC Nze Obiang (Libreville; Centre Hospitalier universitaire mère enfant Fondation Jeanne Ebori). 17 Georgia: Z Demetrashvili, G Devidze, G Pisarevi, L Petashvili, E Ekaladze, N Lekiashvili, I Pipia, A Tvaladze, G Kenchadze, K Khutsishvili (Tbilisi; N.Kipshidze Central University Clinic). Germany: LD Lee (Berlin; Park-Klinik Weissensee); J Binder, A Denz, C Krautz, M Brunner, M Maak, GF Weber, A Stollberg, D Hackner, S Engel, F Krämer, R Grützmann (Erlangen; Universitätsklinikum Erlangen); M Schüler, J Kleeff, R Rüdrich (Halle; University Hospital Halle); A Kirschniak, J Rolinger, S Göller, L Van den Hil , J Miller, H Pehlivan (Moenchengladbach; Kliniken Maria Hilf); M Kießler, N Hüser, D Schippers, M Berlet, M Steffani, M Weber (Munich; Klinikum Rechts der Isar TUM School of Medicine); N Börner, M Albertsmeier, H Arbogast, M Mattis, S Jarmusch, P Zimmermann, U Wirth (Munich; Ludwig Maximilian University of Munich - Großhadern); F Anzinger (Munich; Ludwig Maximilian University of Munich - Innenstadt); FG Bader, M Sohn, ML Koschke, M Busch , N Hielscher (München; Isarklinikum); A Brosin, J Lindert, M Philipp, M Gumsheimer, F Wiese (Rostock; University Hospital Rostock); L Sahan, GA Stavrou, J De Deken, M Jabal (Saarbruecken; Klinikum Saarbruecken); MW Löffler, A Königsrainer, M Quante, C Yurttas (Tuebingen; University Hospital Tuebingen). Ghana: R Armah, NA Christian, D Daary, S Akuffo, A Twumasi, AD Andani , J Oppong, E Agbowada, J Daleku, J Ampadu, W Afedo, Z Robertson, A Obbeng, DN Lee, D Ofosuhene (Accra; Greater Accra Regional Hospital); GD Brown, F Osman, FJ Eshun, C Banka, I Amankwaa, E Ametefe , G Owusu, J Nyamekye - Baidoo , O Okrah, G Birikorang, P Kumassah, J Dei-Asamoa, J Annan, C Akli-Nartey , D Alifoe , S Tsatsu, C Ansah Larbi, U Una, K Yalley, A Bediako Bowan (Accra; Korle-Bu Teaching Hospital); KB Oduro-Boateng , S Anim , H Adjei, S Dognia , A Oppong , C Markin, JL Ahale, NKA Obuobi (Accra; Pentecost Hospital); S Agana , EB Akakpo, F Galley (Ankaful; Ankaful Leprosy General Hospital); FE Gyamfi, S Segnitome , S Agordjor , D Adjei, D Kyeremeh, Y Sarpong, F Opoku Twene, CK Ntow-Boahen, SAA Atupra , V Siepaal , IN Bakaweri, AJ Tabiim , R Agyei Boakye, A Asare Twumasi (Berekum; Berekum Holy Family Hospital); R Akankoatuesi Apatewen, U Kanyan Kassim , B Owusu Ansah, F Amoako (Bolgatanga; Upper East Regional Hospital); V Kudoh, K Boakye-Acheampong , B Boakye, R Kpangkpari, MT Morna, GA Rahman , EO Ofori, L Adagrah Aniakwo, M Amoako-Boateng , D T. Enti, S Debrah, M Nortey, P Koggoh , P Mensah, MM Agyapong, T Agyen, V Etwire, Y Adofo-Asamoah , S Yussif, M Yigah (Cape-Coast; Cape Coast Teaching Hospital); B Maanikuu, F Kuubetersob B. N, D Powell, A Gbeadese , F Tierenye (Damongo; St. Anne’s Hospital); EA Nachelleh, DYD Agbley , N Jiagge , R Akpaka, D Labadah, N Naabo, R Guzmán Lambert, FJ Eshun, P Ntem, E Setsoafia, N Affram, BY Hernandez Cervantes, DB Osei, K Ewool, F Nyarko, MA Ali, MA Oyortey, I Hagbevor, ME Ashong , JN Anyorigiya (Ho; Ho Teaching Hospital); J Yorke, A Lovi, EO Osei, PA Boateng, R Oppong-Amoah, K Agbedinu, C Dally, SG Brenu, F Galley, FM Agbemafoh, I Kyei, C Aboah, AY Appiah-Kubi, B Nimako, M Aikins, M Adinku , A Opoku-Agyapong, J Adjei, R Sagoe (Kumasi; Komfo-Anokye Teaching Hospital); A Gyedu, PK Boateng, S Mensah, E Frimpong-manso (Kumasi; University Hospital, KNUST); P Taah-Amoako, S Tabiri (Nsawkaw; Tain District Hospital); F Owusu, P Yeboah Owusu (Sunyani; Brong-Ahafo Regional Hospital); EMT Yenli, AS Seidu, M Dason , M Amadu, GA Adoro, M Kyereh, I Osman , J Quansah, C Doku, A Darkwa Boateng, AM Muntaka, MA Dokurugu, R Nesco, M Yahaya, EDF Konlan, A Issaka, RA Ramirez Calas , M Sheriff, M Dery, V Dassah (Tamale; Tamale Teaching Hospital); BK Seshie, F 18 Caiquo, M Dum, L Ackam, K Yalley, FDA Agbodo, A Baiden Amissah, D Ashitey , LD Bray, J Ofori, M Ishak (Tema; Tema General Hospital); G Ansong (Walewale; Walewale Government Hospital). Greece: I Gogoulis, K Bekiaridou, A Mitsala, S Botaitis, C Tsalikidis, M Asimakidou, C Nikolaou, E Efremidou, C Limas, P Chloropoulou, M Aggelidou, M Pitiakoudis, P Kostoglou, G Pappas Gogos, M Karanikas (Alexandroupolis; Alexandroupolis University General Hospital); E Kapasakis, M Karakeke, A Skarpas, C Floros, K Athanassiou, E Karakeke (Amfissa; General Hospital of Amfissa); N Tasis, A Sarafi, A Plastiras, G Kavalieratos, T Tsirlis (Athens; Agios Savvas Anticancer Hospital); L Chardalias, N Memos, V Themelidi, I Papaconstantinou, T Theodosopoulos, D Politis, K Iliakopoulos, K Bramis, P Antonakis, A Skreka, D Kotsaris, N Dafnios, A Vezakis, I Contis, T Petropoulou, KC Kordeni, T Kozonis, G Fragulidis, D Massaras (Athens; Aretaieion Hospital); E Apostolopoulos, I Karatsolis, A Mourtzouni, K Avgerinos, D Kelgiorgi, K Polychronopoulos, G Kostoulas, A Tsechpenakis, A Saridaki (Athens; Athens Euroclinic); A Ioannidis, C Chouliaras, I Tierris, MK Konstantinidis (Athens; Athens Medical Center); DK Manatakis, D Balalis, N Stamos, N Tasis, V Kalles (Athens; Athens Naval and Veterans Hospital); T Sidiropoulos, M Papadoliopoulou, N Arkadopoulos, D Sampanis, P Vassiliu, E Dylja, AI Nikolaou, I Margaris, P Kokoropoulos, V Tsaousis, S Christodoulou, E Poulios, A Chamzin (Athens; Attikon University General Hospital); S Kapiris, E Mavrodimitraki, M Sotiropoulou, N Dimitrokallis, N Papadogianni, V Vougas, M Papamichail, K Rekouna, K Pavlopoulos, N Roukounakis, A Thanasa, P Trakosari, M Christou, E Saitoglou (Athens; Evaggelismos General Hospital); C Nastos, D Dellaportas, P Lykoudis, N Garmpis, G Kouraklis (Athens; Evgenideio Hospital); P Christodoulou, G Kapogiannatos, A Nikitaras, SM Tsoti, J Katogiritis (Athens; General Hospital Asklepieio Voulas); EC Tampaki, C Papazacharias, O Bellou (Athens; KAT Athens General Hospital); N Machairas, P Dorovinis, C Doudakmanis, D Schizas, A Syllaios, MD Keramida, P Stamopoulos, S Davakis, M Despotidis, A Panagakis, S Kykalos, F Stavratis, M Vailas, L Karydakis, N Kydonakis, A Loizou, KS Giannakopoulos, P Sakarellos, A Kozadinos, I Katsaros (Athens; Laiko University Hospital); C Damaskos, E Antoniou, M Mavri, I Psilopatis, S Vernadakis , I Bokos, P Paraskeva, D Vardakostas, I Gomatos, A Barlas, A Smyrnis, D Prevezanos, NN Mathioudakis, I Kozadinos, A Kozadinos, P Kanavidis, N Garmpis (Athens; National and Kapodistrian University of Athens); E Spartalis, M Spartalis (Athens; Sotiria General Hospital of Thoracic Diseases); C Stefanou, S Gkogkos, L Fountoulis, I Sougkas, M Billis, A Kontokostopoulos, A Balta, T Padioti , MA Sotiriou, A Theochari (Filiates; General Hospital of Filiates); N Tsakiridis, I Tsakiridis, E Synekidou (Florina; Florina General Hospital ‘Eleni Th. Dimitriou’); E Athanasopoulou, C Ntagkas, E Samara, A Katsiou (Ioannina; University Hospital of Ioannina); D Panagopoulos, A Panagopoulos, L Katsiaras (Kyparissia; General Hospital of Messinia, Hospital Unit of Kyparissia); C Kolla, L Mansour, G Koukoulis, S Zourntou, D Papageorgouli, K Bouliaris, LI Fountarlis, M Bei, AA Kalidis, X Vagena, A Gkouniaroudi, A Migdanis, A Bakalis, E Gavriil (Larissa; General Hospital of Larissa ‘Koutlimpaneio and Triantafylleio’); K Koumarelas, MN Kouliou (Larrisa; General University Hospital of Larissa); K Bouchagier, F Mulita, G Verras, G Skroubis, I Maroulis (Patras; General University Hospital of Patras); V Mousafeiris, A Panagidis (Patras; Karamandaneio Prefecture Children Hospital of Patras); A Papadopoulos, P Grivas, F Spanos, A Kalogeropoulou , G Zeringa, C Kourouniotis, G Rados, E Barkolias , K Zakkas, I Demiris, V Nikolaou, K Tata, G Karakaidos (Piraeus; General Hospital of 19 Nikaia); E Kontis, E Papamattheou, A Efstathiou, E Efstathiou, N Kopanakis, K Ntatsis, I Katsaros, P Manikis, V Tselepidis, M Kyriazi (Piraeus; Metaxa Cancer Hospital); I Siannis, N Kouzakos, V Georgilaki , N Vlachakos, S Vederaki, A Tsiaka, A Zarafidou, N Zampitis, S Tsatsos, F Stefou, F Kyramargios, M Merrakos, G Bekakos, A Marinis (Piraeus; Tzaneio General Hospital); O Ioannidis, E Anestiadou, K Zapsalis, S Simeonidis, S Bitsianis (Thessaloniki; George Papanikolaou General Hospital of Thessaloniki); M Drogouti, A Gkoutoula, A Sarakatsanos, E Efthymiou, I Chatzis (Thessaloniki; O Agios Dimitrios General Hospital); I Spyridakis, C Kaselas, M Tsopozidi, M Florou, C Demiri, V Papadopoulos, D Giakoustidis, A Giakoustidis, D Alexandrou, P Chatzikomnitsa (Thessaloniki; Papageorgiou General Hospital); SC Liapis, K Perivoliotis, C Chatzinikolaou, N Tsantikos, ZR Karampotaki , D Lytras (Volos; Achillopoyleio General Hospital of Volos). Guatemala: M Aguilera-Arevalo, M Rodríguez-Ordoñez, DA Sosa Méndez, M Sebastián-Mendoza, TA Salazar-Lorenzana, R Herrera, DE Reyes Rodríguez, P Vásquez , S Morales , J Gomez , C García-Salas, JR Asturias Luna, J Tabora-Zepeda, S Vasquez, JR Hernández , JOM Herrera Batres , D Muñoz, E Ayala , O Coyoy-Gaitán (Guatemala City; Hospital General San Juan De Dios); JB Pellecer Cano, DA Palma Portillo, M Blanco (Guatemala City; Hospital Juan Jose Arevalo Bermejo); D Herrera, SA Villeda , O Lima Azurdia (Guatemala City; Hospital de Referencia Nacional de Enfermedades Respiratorias). India: S Jp, M Bhat, A Raheja, I Shariff, H Anand , B R Budihal, S Kashyap, B Arya, Y M S, N Krishnappa (Bangalore, Karnataka; BGS Global Institute of Medical Sciences); M Manangi, P Anandan, S Shivashankar chikkanayakanahalli, S Kumar Venkatappa (Bangalore; Victoria Hospital); TS Mishra, P Kumar, M Gureh, MK Sethi, AA Asharaf (Bhubaneswar; All India Institute Of Medical Sciences - Bhubaneswar); Y Sakaray, S Irrinki, S Subbiah Nagaraj, S Khare, C Tandup (Chandigarh; Postgraduate Institute of Medical Education & Research, Chandigarh, India); M K B, AB Muthunayagam, A Prasath S V , P Arunachalam, P John (Coimbatore; PSG Institute of Medical Sciences and Research); S Raul, R Vakil, R Sinha, E Dvivedi, A Thomas, S Joseph, A Sharma, B Khan, D Chatterjee (DELHI; St Stephen’s Hospital); R Gupta, A Khanduri, S Singh, DH Tyagi , U Daspal, N Rawal, R Varshney (Dehradun; Synergy Institute of Medical Sciences); M Luthra, R Handa, S Basu , P Chadha, R Sethi (Delhi; Holy Family Hospital); T Longkumer, KK Mishra, S Sundaramurthy, S Kumar, D Phom, J Kaippally, D Ommi, L Imchen, T Alinger (Dimapur; Christian Institute of Health Sciences and Research); A Chhabra, A Kumar (Faridkot; Guru Gobind Singh Medical College & Hospital (Baba Farid University of Health Sciences)); B Kharga, M Sarda, K Bhutia (Gangtok; Sir Thutob Namgyal Memorial Hospital Sochakgang); N Sharma, MS Rodha, N Banerjee, A Baksi, S Kaur, R Chaudhary, M Lodha, SP Meena, M Badkur, I Singh, A Sinha, KJ Rathod, R Saxena, J Tk, A Vig, M Pathak, A Sukhdev Jadhav, S Nayak, T Motiwala, K Shreyas (Jodhpur; All India Institute of Medical Sciences (AIIMS), Jodhpur); SR Pathan, J Rathod, C Agarwal, K Sharma, S Pandya (Karamsad; Shree Krishna Hospital); A Anand, A Kumar, HS Pahwa, AA Sonkar, MK Agrawal, AK Pal (Lucknow; King George’s Medical University); D Jain, PD Haque, V Michael, W Bhatti, J Dhiman, DRS Thind, A Bhatt, P Gupta, A Luther, S Khurana, RR Ranadive, A Suroy, H Kaur, S D A, P Shukla (Ludhiana; Christian Medical College & Hospital); NK Chaudhry, DA Hajela, P Patel, PK Arya, DD Dhawan, DR Tripathi, KK Luthra, A Kumar, DH Gupta (Ludhiana; Satguru Partap Singh 20 Hospital); A Mathew, C Pun, P Dummala, M Gurung (Madhepura; Madhepura Christian Hospital); P Alexander, N Aruldas (Manali; Lady Willingdon Hospital); PS Prabhu, S Payyanur Thotan, B L, B Sv (Manipal; Kasturba Medical College Hospital, Manipal); RD Sharma, R Redkar, R Nathani, S Karmarkar, A Sharma, S Singh, S Achugatla, A Bangar, DD Kulkarni, K Raghuwanshi, DN Nikam, SN Mahendra (Mumbai; Lilavati Hospital & Research Centre); B Sarang, D Belekar, K Gaikwad (Mumbai; Terna Medical College and Hospital); A John, PA Thomas, L Pramod, D Gavit, D Singh (Nandurbar; Chinchpada Christian Hospital); N Kansakar, N Gupta, N Kapur, N Narain (New Delhi; ABVIMS Dr RML Hospital); S Kulkarni (New Delhi; Army Hospital Research & Referral New Delhi); T Rashid, M Husain, F Tauheed, SV Manzoor , S Ohri (New Delhi; Hamdard Institute of Medical Sciences & Research); L Bains, P Lal, S Neogi, A Mishra, S Ahuja (New Delhi; Maulana Azad Medical College); T Iahmo, M George, M Singh , P Waghchoure, A Choudhrie (Padhar; Padhar Hospital); A Kumar, M Aggarwal, V Kanna D, S Vembar, TP Singh, DS Walia, V Singh, G Kaur, A Jindal, P Dhamija (Patiala; Government Medical College Patiala); M Kumar, A Sinha, AK Jha, M Sharma, A Bhadani (Patna; All India Institute of Medical Sciences, Patna); R Abhinaya, U Kumbhar, A Jain, S Chilaka , S P (Pondicherry; Jawaharlal Institute of Postgraduate Medical Education and Research); VS Jha, A Jayapalan, Y Vashishth, G Jalal, VV Nair, C Raphael , HK Prabhakar, Z Khan (Pune; Command Hospital, Southern Command); D Dugar, D Mohanty, TDB Tridip, DR Ramchandani (Raipur; All India Institute of Medical Sciences Raipur); S Basu, N Kumar, AG Goswami, H Panga, D Mallik, A Gupta, D Rajput, R Anjum T Siddeek, P Manjunath, S Edem, F Huda, SK Singh, S Karuppusamy Krishnasamy, S Katragadda, KMR Reddy, I Ahmed, E Yhoshu, L Manoj Joshua, A Das, P Kothari (Rishikesh; All India Institute Of Medical Sciences); K Singh , SS Malhi, R Kaur, HK Cheema, M Singh, N Saini, M Gupta, A Bhatti, A Gupta, P Kaur, N Pahuja , H Kaur, S Chopra, B Sehgal, G Singh (SAS Nagar (Mohali) ; BR Ambedkar State Institute of Medical Sciences Mohali); DS Kshirsagar, M Kaple, G Saxena, S Dhole, A Bhargava, C Mahakalkar, S Deshpande (Sawangi (Meghe), Wardha; Acharya Vinoba Bhave Rural Hospital); R Wani, RA Dar, AA Malik, N Bhat, ZA Shah, GA Bhat (Srinagar; Sheri-Kashmir Institute of Medical Sciences); S Kondpan, A Kutma, JA Kalyanapu (Tezpur; Baptist Christian Hospital); A Sundaram, K J B, R Krishna raj, G George, M Chisthi, H Jafarkhan, I P s, U Govindan, MKL K S D, C Narayan, G Pillai, VV Kollengode, D Jabbar (Thiruvananthapuram; Government Medical College Thiruvananthapuram); T Tony V, A Nair, A Kavalakat, A Moncy, A Johnson, A Joseph, A Appukuttan, A J, AN Oommen, S Francis, S M, N Srinivas, M Narayanan (Thrissur; Jubilee Mission Medical College & Research Institute); T Devabalan Koil, MR Jesudason, Y Myla, ASP Dhinakar, A Tirkey, B Roopavathana. S, R Mittal, S Surendran, N Paul Ambrose, D Joshiba, P Trinity, R Raghunath, NP Paul Sigamony, PY George, R Philip Sridhar, S Chase, SJ Arthur (Vellore; Christian Medical College & Hospital). Iran, Islamic Rep.: N Yousefzadeh Kandevani (Bastak; Farabi hospital); M Pourfridoni, H Askarpour, H Mohammadi sardoo, AA Kheirkhah Vakilabad, M Ali-Hassanzadeh (Jiroft; Imam Khomeini Hospital). Iraq: R Raheem Attallah Al_obaidy (Anbar; Heet General hospital); Z Alkhuzaie, S Salim , FRH Hassooni (Najaf; Al Batool private hospital); Y Zwain , HMA Oneizah, S Razaq, M Razaq, HHZ .zaini (Najaf; Al-Najaf Al-Ashraf Teaching Hospital). 21 Ireland: E Colton, KE Oderoha, A Gill, S Ramjit, F Ghazali, K Griffin, R Ahmed, C Weadick, S Nair, V Sharma, C Donohoe (Dublin; St James’s Hospital); R Tummon, B Maguire, S Wrenn, R Habib, P Owens (Kerry; University Hospital Kerry). Israel: A Shweiki, G Szydlo Shein, G Almogy, Y Mintz, A Pikarsky, R Elazary, O Cohen-Arazi, JA Demma, H Mahajna, B Helou, Y Fishman, G Marom (Jerusalem; Hadassah Medical Center). Italy: M De Prizio, K Kröning, R Sulce, LM Fatucchi, F Tofani, V Mariottini, R Malatesti, M Scricciolo, GA Pellicano’, A D’Ignazio, A Mazzoni, A Biancafarina, M Angelini, V Borgogni (Arezzo; Ospedale San Donato USL Toscana Sud Est); L Rossi, G Munzi, G Tarantino, M Castrovillari, A Serao , JR Casella Mariolo, G Del Corpo, A Natili, A Iodice (Ariccia; Ospedale dei Castelli (N.O.C.)); S Gargiulo, B Esposito, M Pannullo, L Bracciano, E Marra, A Alberico (Aversa; San Giuseppe Moscati); A Gori, S Cardelli, G Dajti, C Larotonda, IS Russo (Bologna; IRCCS Azienda Ospedaliero-Universitaria di Bologna); J Andreuccetti, S Molfino, D Alberti, G Pignata, G Emiliani, G Boroni, M Ruffoli, M Manfredini, L Sequi, G Zanni (Brescia; ASST Spedali Civili, Ospedale di Brescia); E Locci, M Podda, V Murzi, C Piras , A Carta, A Pisanu, T Pilia, P Marongiu, A Saba, M Pisano, F Campus, E Gessa, E Silanos, A Lai, F Frongia, F Corronca , S Montisci (Cagliari; Cagliari University Hospital); F Cappellacci, C Soddu, GL Canu, F Medas, PG Calò, S Puddu, M Biancu, M Abbas, F Casti (Cagliari; Chirurgia Generale e Polispecialistica, Cagliari University Ospital ‘Duilio Casula’); B Demurtas, A Deserra, F D’Agostino, C Margiani (Cagliari; Santissima Trinità - ATS Sardegna); L Laface, M Casati, M Mariani, S Guarriello, A Balconi (Carate Brianza (MB); Ospedale Vittorio Emanuele III - Carate Brianza); B Scotto, N Laquatra, R Ruccella, G De Angeli (Carpi; Ramazzini); G Gravante, A Chiappini , R Lopatriello, G Mammolo (Casarano; Francesco Ferrari Hospital); C Distefano, G Riccioli, R Granata, M Veroux, DC Centonze, S Costa, R Gioco, D Zerbo, A Licciardello, L Stella (Catania; Azienda Ospedaliero- Universitaria Policlinico San Marco); L Rende, M Osso, D Paglione, F Pata (Cosenza; Azienda Ospedaliera di Cosenza); D Sasia, G Giraudo, D Ribero, S Alberti, V Schirinzi (Cuneo; Santa Croce e Carle Hospital, Cuneo); P Belotti, L Taglietti (Esine; ASST Valcamonica Ospedale di Esine); V Giordano, A Pesce, CV Feo, MC Pignanelli, S Severi (Ferrara; Azienda Unità Sanitaria Locale di Ferrara); F Cammelli, F Natali, M Scheiterle, G Maltinti, L Fortuna, F Coratti, A Manetti , J Martellucci, E Monati (Firenze; Azienda Ospedaliera Universitaria Careggi); L Gabellini, R Fratarcangeli, A Damigella, E Adinolfi, A Anastasi (Firenze; Ospedale San Giovanni di Dio); M Montagna, A Giuliani, G Procaccini, N Tartaglia, F Vovola, D Merlicco, S Schirone , ST Massa, G Pavone, M Pacilli, F Maffei, A Gerundo (Foggia; Ospedali Riuniti Azienda Ospedaliera Universitaria); D Di Pietrantonio, S Quartarone , L Solaini, G Ercolani, L Ragazzini, V Zucchini (Forlì; MorgagniPierantoni); M Cammelli, D Scotto Di Carlo, FAN Marin (Garbagnate Milanese; ASST Rhodense - Ospedale di Garbagnate Milanese); A Azzinnaro, A Razzore, A Petrungaro, E Mina, B Sperotto (Genoa; E.O. Ospedali Galliera); G Carganico, D Pertile, D Soriero (Genoa; IRCCS Ospedale Policlinico San Martino); R Diaz, A Luzzi, S Carrabetta, C Meola, D Caruso, F Floris, P Grondona, E Romairone, S Marzorati, F Ré, C Righetti, A Viacava, L Epis (Genoa; Ospedale Villa Scassi); R Sampietro, D Gobatti, C Zandonella (Gravedona ed Uniti; Ospedale Moriggia Pelascini); VP Dinuzzi, U Rivolta, S Luciano, GMF Marini, L Scaravilli (Magenta; Ospedale ‘G.Fornaroli’, ASST-OVEST Milanese); R Magarini, G Saletta, AC Sironi, G Grava, M Mercurio (Melzo; Ospedale Santa Maria 22 delle Stelle, ASST Melegnano Martesana); D Zulian, A Izzo, M Gritti, S Giudici, E Desiato (Milan; Humanitas Research Hospital); M Molteni, L Ottaviani (Milan; IRCCS San Raffaele Scientific Institute, Milan); G Grande, E Mazzotta (Milan; Ospedale Fatebenefratelli e Oftalmico); S Grimaldi (Milan; San Carlo Borromeo); F Brucchi, F Ferraina, S Lauricella (Milan; Sesto San Giovanni Hospital); E Di Marco (Modica; Ospedale Maggiore); LC Nespoli, G De Carlo, EA Baccalini, D Palmisano, A Scacchi, N Tamini, L Ripamonti, M Rennis, C Vitiello, A Davolio, L Degrate, P Masseria, V Brocco, P Chiacchio, M Ceresoli, E Signaroli, A Finocchio, C Fumagalli, M Binda (Monza; Fondazione IRCCS San Gerardo dei Tintori Monza, Scuola di Medicina e Chirurgia, Università Milano Bicocca); M Milone, M Manigrasso, S Vertaldi, A D’Amore, GD De Palma, A Marello, L Fedele, C Sorrentino, D Pignatelli , G Luglio, FP Tropeano, M Cricrì, A Miele, G Aprea, G Palomba, M Capuano, R Basile, G Sorrentino (Naples; Federico II University of Naples); D Rega, A Ottaiano, V Granata, A Belli, F Izzo (Naples; Istituto Nazionale Tumori Fondazione, Pascale IRCCS); G Pellino, D Massaro, V Mosca, F Selvaggi (Naples; Primo Policlinico di Napoli); G Bellio, L Rubin, N De Santis, N Schiavon, A Zerbinati, S Corso, C Cecconi (Padova; Piove di Sacco Hospital); P Venturelli, G Cocorullo, G Carollo, R Tutino, A Bonelli, G Salamone, MP Proclamà, R Guercio, L Licari, N Finocchiaro, G Graziano, G Orlando, G Guercio, M Marcianò, G Galatioto, F Vassallo (Palermo; Policlinico Universitario Paolo Giaccone); G Palmieri, F Banchini (Piacenza; G. Da Saliceto); G Di Franco, F Porcelli, N Furbetta, A Comandatore, S Guadagni, M Palmeri (Pisa; Azienda Ospedaliero Universitaria Pisana); P Ubiali, F Maffeis, J Velkoski (Pordenone; Azienda Sanitaria Friuli Occidentale (AS FO)); M Giuffrida, GE Nita (Reggio Emilia; Azienda Unità Sanitaria Locale - IRCCS di Reggio Emilia); C Marafante, M Garino, SL Birolo , M Dugo, M Pisano, A Borello, R Barone, LD Bonomo, MV Facchino, M Caccetta, E Moggia, MR D’Anna, C Mosca, S Mungo, A Masciandaro (Rivoli; Ospedale degli Infermi di Rivoli); F Tirelli, I Neri , M Aulicino, C Vacca (Rome; Fondazione Policlinico Universitario Agostino Gemelli); M Campanelli, M Grande, L Siragusa, G Sica (Rome; Policlinico Tor Vergata Hospital, Rome); A Mingoli, G Brachini, B Cirillo, S Meneghini, S Giovampietro , G Duranti, F Ciccarone, L Simonelli, I Clementi, B Binda, GB Fonsi, E Spalice , E Cianci, MI Bellini, G Sgarzini, S Sorrenti, E Lori, P Palumbo, D Pironi (Rome; Policlinico Umberto I Sapienza University of Rome); A Amendola, C De Martino, E Bisogno (Salerno; San Giovanni di Dio e Ruggi d’Aragona); S Di Saverio, A Morello, L Lely, I Merlini, G Travaglini , S Sabbatini, M Zambon (San Benedetto del Tronto; Madonna del Soccorso Hospital); D Giulitti, L Barni, GE Poto (Sansepolcro; Valtiberina); D Fusario, L Resca, L Carbone, A Francia, GE Poto, AL Pesce, O Carpineto Samorani, F Roviello, A Ongaro, SA Piccioni, M Gambelli, L Catozzi, M Gjoka, A Bartalini Cinughi de Pazzi, NN Leonelle Lore, G Grassi, F Manasci, V Ricchiuti (Siena; Azienda Ospedaliero Universitaria Senese); E Basile, A Spaziani (Spoleto (PG); San Matteo degli Infermi); V Silvestri, M Favoriti, P Favoriti (Sulmona; SS Annunziata); S Novello, M Piccino, R Baldan, U Grossi, G Zanus, F Scolari, E De Leo, M Brizzolari, A Brun-Peressut, I Hoxhaj, M Scopelliti (Treviso; Ospedale Ca’ Foncello - Università di Padova (DISCOG)); LB Lo Piccolo, E Montanari, D Cianflocca, A Marano, S Galati, SL Gamba, F Velluti, A Caltagirone, M Giuliano , E Potenza, B De Zolt Ponte, D Visconti, VU De Donato, L Capello, S Chaifouroosh Mamagany, C Celano (Turin; Città della Salute e della Scienza); E Donnarumma, C Saviello, R Scola (Vallo Della Lucania; Casa di Cura Prof. Dott. Luigi Cobellis); S Megna, M Berselli, N Palamara, L Liepa, E Ferri (Varese Lombardy; University of 23 Insubria, Ospedale di Circolo e Fondazione Macchi (Varese)); V Gentilino, M Mogiatti, G Farris, N Pasqua (Varese; Filippo Del Ponte Hospital, University of Insubria); A Iacomino (Venezia; Ospedale Civile - Santi Giovanni e Paolo); I Mondi, C Da Lio, F Sulo, E Ciccioli, D Verdi (Venice; Mirano Hospital); M Martorana, M Filardo, L Schiavone (Voghera; Ospedale Civile di Voghera); I Conversano, M Cappiello, G Scialandrone, N Petrarota, R Tumolo (andria; Lorenzo Bonomo); M Angelucci, S Valeri, G Pascarella, A Strumia, R Alloni (rome; policlinico universitario campus bio medico of rome); D Muschitiello, V Morinelli, L Bonello, SG Intini, S Moschella (udine; santa maria della misericordia). Japan: H Kato, A Horiguchi, D Koike, Y Asano (Aichi; Fujita Health University Bantane Hospital). Jordan: S Alananzeh, S Al Momani, M Tanashat, O Altobaishat, S Alsmadi, N Al RABADI, L Sweidan (Ajloun; Al Iman Hospital); Z Alnajjar, G Alsheikh, N Mosleh, H Alzuhd, R Alkhatib, H Al-Abdallat, M Aljarawn, T Aloqaili, I Nadi, A Abdllah, O Al-Fahel, R Khalil, M Said, A Qasem, H Al-Fahel, M Hijazi, R Rabah, A Alaqtash, DB Badwan (Amman; Al-Basheer Hospital); Y Alawneh (Amman; Ibn Al Haitham Hospital); B Alrayes, M Salah, M Al-Qannas, H Abu Obead, I Alnimer, Y Alawneh, M Almaletti (Amman; Islamic Hospital); A Khamees, R Yousef Yassin, A Alsheikh, K Al-Shami, E Abu Siam, O Sarhan, KA Sawaftah, MAM Sawaftah, MA Sawaftah, M Sabri Massadi, Z Al-sheikh ali, N Raiq, O Ibrahim, J Al Karmi, M Diab, I Aburumman , AA Altawaiha , M Hasan, A AlZu’bi, L Yasin, B Yacoub (Amman; Jordan University Hospital); R Abu Salah, M Alqedrh , S Abu khousa, MEH Albanna (Amman; Marka Specialty Hospital); R Hussam Yacoub Hattar, D Samardali, R Refaie, L Hijazein , S Hammad, M Barbarawi, S Mamduh, J Al Daradkah, S Samardali , T Alshawabkeh (Amman; Prince Hamza hospital); M Mahafdah, Q Sabbah, SAM Ba-Shammakh, A Al Hammoud, M Bani hani, M Tabaza, H Malkawi, H Haj Freej, D Kasasbeh, B Dweik, K Ayyoub, OR Mahafdah (Ar Ramtha; King Abdullah University Hospital/ Jordan University of Science and Technology); R Hiary, I Shehadeh, L Dyab, R Daradkeh, R Raddad, R Alzu’bi, T Alhaj Hasan , B Alzoubi, M Alsharayri, M Nofal, S Ellouzy, M Al-Masri, S Fakhouri, N Absy, R Suleiman, O Mansour, JS Hadidi (As-Salt; Al Hussain New Salt Hospital); A Al-fandi (Irbid; Ar Ramtha Govermental Hospital); M Al-Fraijat, N Rabai, A Al-Zubeidy, R Abd Elkareem, S Bani Amer , T Alhusban, S AL-Doghme, R Abd Alkareem, R Damseh, M Alshami , T Majed, S Al Sharie, M Araydah, R Jaba’Teh, F Haddad, O Almomani, L M Mheidat, R Haddad, S Bataineh, S Ababneh (Irbid; Princess Basma Hospital). Kazakhstan: M Kulimbet, N Maulenov, N Lakhanov, M Ramazanov, A Kiyabayev (Almaty; City Clinical Hospital No.7, Asfendiyarov Kazakh National Medical University); D Amangaliyev, A Shamsutdinova, A Polatbekov (Almaty; JSC ‘Central Clinical Hospital’, Asfendiyarov Kazakh National Medical University). Kenya: R Parker, E Irungu, A Fadipe, F Ondago, G Waiyaki (Bomet; Tenwek Hospital). Lebanon: A Khoneisser, A Kachi, B Abboud (Beirut; Hopital Libanais Geitaoui); M Chaccour, G Bechara, N Eshak, R Hleyhel, M Barakat (Jbail ; Maritime Hospital). Libya: N Lindi, BH Hameed, A Abaidalla, N Mosbah, M Saleh khatab (Albayda; Albayda Medical Center); MAM Elghriani, A Ali, A Fathi, S Qwyder, M Saleh (Benghazi; Al-jalaa Teaching/Trauma Hospital); M Alshamikh, A Alhammali, A Aldurssi, A Gusibat, M Suleman, S Alashhab, M Denini, F Alowjaly, MM Almihashhish, F Elkhafeefi , H Altawati (Benghazi; Benghazi Children’s Hospital); S 24 Elfallah, F Benghalbon, R Michael , M Abosedra, A Ahmayda, H Mftah (Benghazi; Benghazi Medical Center); M Bohlala, M Muragi, S Alneihuom (Darna; Al-Wahda Hospital); A Alkaseek, A Alshiteewi, H Shames, H Bileid Bakeer (Gharyan; Gharyan Central Hospital); N Albahloul, M Abudabbous, A Belkhair, A Abdelmalik, M Assalhi, M Altajouri, A Alailesh , A Alshukre (Misurata; Misurata Central Hospital); B Alazabi, M Alazabi, G Birqeeq, AAY Almugaddami, A Egdeer (Nalut; Nalut Central Hospital); H Embarek, M Bilfaqirah (Sebha; Al-Majd Clinic); M Abdelkabir, S Abdeewi , A Abdalhadi, M Benghazi (Sebha; Aseel Alghad Clinic); H Idheiraj, M Yahmad, M Alfaid, M Abdu, E Abdu, M Khalifa, G Matroud, A Amaigl , H Aldare, E Ali, K Shwail, K Abdulrahman (Sebha; Sabha Medical Center); A Bouhuwaish, A Emran, A Abdraba (Tobruk; Tobruk Medical Center); AE Elzoubi, A Belaid (Tripoli; Alkhalil hospital); A Alragheai, D Omar, S Magrhi, H Farhat, S Alsuwiyah , S Abrayik, S Bensalem, B Algettawi (Tripoli; Metiga Hospital); S Egreara, TMA Abdulmola, I Kandil (Tripoli; Sabratha teaching hospital); F Alshreef, F Elhabishi , M Alsori, L Shawesh , S Timmalah, M Alnuwayli, A Alhamadi, K Ahmed Ibrahim, S Abdullateef, R Altayargh, A Essamei, S Altoume, A Haidar, M Khalil, A Abdulnabi, S Mohammed , A Ghummied (Tripoli; Tripoli Medical Center/ Tripoli University Hospital); E Younes, S Elfurdag , A Ali, S Ashini, M Edeeb, Z Al-azher El-hamel, M Akkawe , N AlWAER, A Khair Etareig (Zawia; Zawia Teaching Hospital); B Allbakosh, H Abusnina, A Awidan, L Alokshi, M Iqreewi , M Almahjoub , H Altounsi, M Almaqrahi (Zliten; Zliten Teaching Hospital). Lithuania: E Dainius, S Bradulskis, E Margelis, A Mačiulaitytė, A Subocius, A Parseliunas, E Kubiliute, D Zuikyte, J Kutkevičius, J Vaitekūnas (Kaunas; LUHS Kaunas Hospital); L Venclauskas, K Jasaitis, M Jokubauskas, Z Dauksa (Kaunas; Lithuanian University of Health Sciences Kaunas Clinics); A Gulla, E Daukšaitė (Vilnius; Vilnius University Hospital). Madagascar: MJ Rakotonaivo, CF Rahantasoa Finaritra, JB Razafindrahita, YM Razafimandimby , A Rakotondrainibe (Antananarivo; Joseph Ravoahangy Andrianavalona Hospital). Malaysia: AD Zakaria, MIS Ismail (Kelantan; Hospital Universiti Sains Malaysia); R Noor, M Che yaacob, SF Moh Pauzi, Z Chin (Kota Bharu; Hospital Raja Perempuan Zainab II); K Voon, JH Fu, JH Lim, SA Theivendran, NN Ramli (Kuching, Sarawak; Sarawak General Hospital); M Fitri, MA Yunus, AN Ramly, A Md Yunos, AAA Anuar (Malacca; Hospital Jasin); NS Abd Ghani, AAH Ahmad Zaidi, F Ashraf, M Mahadi, AA Abdul Rahim (Serdang; Hospital Pengajar Universiti Putra Malaysia (HPUPM)). Mali: OAA Dicko, A Dembele, H Dolo, D Kone (Ségou; District Hospital of Tominian). Malta: C Cini, A Sultana, M Farrugia, J Schembri Higgans, S Bowman, J Psaila, P Andrejevic, S Brincat, K Muscat, M Sammut, R Abela, M Zammit Vincenti, L Casingena, J Galea, M Portelli, M Sammut, N Spiteri, K Iles, R Cachia, D Hili (Msida; Mater Dei Hospital). Mexico: TR Ibarra-Hurtado (Guadalajara; Antiguo Hospital Civil de Guadalajara); LA Flores Chávez, JA Flores Prado, K Jasso García, NE López Bernal, EV Romo Ascencio, LM Flores Chávez, MP Mellado Tellez, SA Ibarra Camargo, G Delgado Hernandez, JA Guzman Barba, LA Rea Bocanegra, M Tello Jimenez, JA Tavares Ortega, E Gómez Mejía, I Esparza Estrada, AA Salinas Barragan, JA Jimenez Flores, SJ Vázquez-Sánchez, J Gonzalez Garcia , ZM Correa López, FJ Barbosa Camacho (Guadalajara; Clínica de Especialidades más Centro de Cirugía Simplificada); CM Nuño-Guzmán, AM Nava Franco, JF Martinez Martin del Campo , JJ Ulloa Robles , L Bravo, ME Gonzalez-Gonzalez, FD Romo Rosales, TR Ibarra-Hurtado, LG Peña Balboa, C Yanowsky-Gonzalez, R Santana Ortiz, SA 25 Trujillo Ponce, J Orozco-Perez, MDC Gonzalez, JE Gonzalez Aboytes, J Pizarro Lozano , JE Orozco Navarro, F Ibañez Ortiz , O Montaño Angeles, M Calderon, F Diaz , M Lazo Ramírez , JA Aguilar (Guadalajara; Hospital Civil Fray Antonio Alcalde); A Gonzalez Ojeda, C Fuentes Orozco, JM Chejfec-Ciociano, JM Carranza Rosales, MA Sánchez Audelo, CI Lupercio Figueroa, KV Ascencio Diaz, CE Gutierrez de la Rosa, F Mercado Sanchez, FY González Ponce, R Mares País, C González Baez, LÁ Pelayo Orozco, NG Barrera Lopez, A Ramírez Beas, MÁ Zaragoza Mendieta, MF Zarate casas , SL Trejo Ramos, P Salas Núñez, JA Gutiérrez Gómez (Guadalajara; Hospital de Especialidades, CMNO-IMSS); G Ambriz González, I Cabrera, HB Moya- Ambriz , FJ Silva Rivera, EM Torres De Anda, A Hernández, FJ León Frutos, VE Armenta Tapia, M Nieto Galvan, JM Alvarez Hernandez (Guadalajara; UMAE Hospital de Pediatria Centro Medico Nacional de Occidentes IMSS); AI Sánchez-Terán, N Muñoz Montes, AN Fuertes Muñoz, RL Smolinski kurek (León; Hospital Regional e Alta Especialidad del Bajio); K Bozada-Gutiérrez , A Nuñez Venzor, A Zubillaga-Mares, I Serrano (Mexico City; Hospital General Dr. Manuel Gea González); C Moreno-Licea, S Anaya Sanchez, A Trigos Díaz, CJ Pérez - Padrón, EY García-Villegas., RH Perez-Soto (Mexico City; Instituto Nacional de Ciencias Médicas y Nutrición ‘Salvador Zubirán’); MJ Rueda Medécigo, A Leon - del- Angel, HDJ Pérez Baca, C Chavarría Noya , L Castro (Pachuca; Sociedad Española de Beneficencia); D Herappe , MT Barrio Renteria (Querétaro; Hospital de especialidades del niño y la mujer); A Ramos-De la Medina, L Martinez, II Durán Sánchez , DS Gonzalez , MJ Martínez (Veracruz; Hospital Español Veracruz). Morocco: M Berrakkouch, F Hourri, A Benmansour, R Ait Ben Addi , M Melouane, A Tariq, O Boujidi, I Zerrouq , M Katif, S Amahmid, S Errami, S Jamil, O Nouhail, H Essalim, A Nidali, N Ouachou (Marrakech; Centre Hospitalier Universitaire Mohammed VI, Marrakech); A Aboumedian , S Kessab, N Lahnaoui, O Arsalan, S Kassad, A Hrora (Rabat; Centre Hospitalier Universitaire Ibn Sina Rabat). Namibia: JT Abebrese, M Van der Colf, FW Quayson , P Shimbulu, P Nambala (Windhoek; Windhoek Central Academic Hospital). New Zealand: S Rennie, A Herewini (Masterton; Wairarapa Hospital); S Kosna, A Olsen, J Gillingham, R Campbell, A Lin (Wellington; Wellington Regional Hospital); T Uiyapat, S Beavis, D Bardsley, M Lill (Whanganui; Whanganui Hospital); J McNab-Hand, O Ray, C Harmston (Whangarei; Whangarei Hospital). Nigeria: A Adeyeye, A Akinmade, E Afeikhena, AI Okunlola (Ado Ekiti; Afe Babalola University MultiSystem Hospital); D Idowu, J Olorunfunmi, A Olabode (Ado-Ekiti; Ekiti State University Teaching Hospital); N Oloko, KJ Bwala, A Ningi (Bauchi; Abubakar Tafawa Balewa University Teaching Hospital Bauchi); P Agbonrofo, D Osifo , P Idjerhe , BO Izedomi, O Omoike, O Irowa, S Ideh, J Enaholo, C Agbonrofo , O Emuze , PV Odigie, M Ediale, RA Eghonghon, M Edena, A Ekpeti , M Momoh, C Osime, O Osagie, A Arekhandia, M Ibadin (Benin City; University of Benin Teaching Hospital); T AbdulRahman, R Ediru, J Abutu John, O Owolanke (Bida; Federal Medical Centre Bida); U Ezomike, N Agugua-Obianyo, J Ede, S Aliozor, EI Nwangwu, C Ilo, C Amah, L Onyebulu, I Ugwueke , I Obianyo, C Onwuzu, U Dilibe , I Orji, V Enemuo, N Celestine , N Ekwo (Enugu; University of Nigeria Teaching Hospital); SA Sani, S Olori, I Pius Ogolekwu , O Attawodi , R Hauwa SANI, P Chimezie Andrew (Gwagwalada; University of Abuja Teaching Hospital); A Ishola, O 26 Ayandipo, N Akinbami, A Fakoya, TA Lawal, V Osoka, H Ogundipe (Ibadan; University College Hospital); A Ademuyiwa, F Alakaloko, O Oluseye , N Duru, M Ojo, T Olobatoke , AO Lawal, C Nwanmah , O Alaba, R Eloka, C Bode, O Elebute, J Seyi-Olajide, K Onyekachi, O Balogun, O Christianah, L Omomeji , F Akinwande, A Damola-Okesiji, J Okei (Idi Araba; Lagos University Teaching Hospital); H Abiyere, O Fatudimu, B Mustapha, O Babatunde, AI Okunlola (Ido Ekiti; Federal Teaching Hospital, Ido Ekiti); OM Williams, O Faboya, C Ónyeka, F Oni, K Shodunke, G Eke, M Abdulsalam , O Oso, A Ayodele, M Okechukwu (Ikeja; Lagos State University Teaching Hospital); C Adumah, A Talabi, O Oyinloye, O Olajide , V Agbakwuru, A Aderounmu, A Agbaje, YL Balogun, MO Ameen, DO Komolafe, O Olasehinde, O Ajiboye, M Fagbayimu, G Aduroja, O Fasoro, A Adisa, AM Olugbami, H Oyinlola, E Adebunmi (Ile-Ife; Obafemi Awolowo University Teaching Hospitals Complex); T Mohammed, A Lawal, F Bello, P Adebayo, O Salako, A Akinkuolie, MA Adetoyi, A Akeem Aderogba, T Oyeyemi , O Ojo (Ilesa; Obafemi Awolowo University Teaching Hospitals Complex Wesley Guild Hospital Unit); E Osaze, H Ekwuazi , I Ogundele, P Elemile, A Ayeni, I Okoro , C Onuoha (Ilishan-Remo; Babcock University Teaching Hospital); M Mobolaji-Ojibara , J Mohammad mohammad (Ilorin; General Hospital); NT Abdulraheem, A Jimoh , A Lawal, OK Fasiku (Ilorin; University of Ilorin Teaching Hospital); B Aminu , S Kache, G Yohanna Abrak (Kaduna; Barau Dikko Teaching Hospital); AA Sheshe, L Anyanwu, AB Muhammad, A Abubakar Abdulkarim , TN Nagwamutse, IU Garzali, S Muhammad, C Nwachukwu, IE Suleiman, M Abdullahi , SA Aji, A Dahiru, LB Abdullahi, SA Yunusa , A Yahaya, M Bello, I Wasiu, U Mohammed Bello, B Yunusa, N Umar (Kano; Aminu Kano Teaching Hospital); RE Enejo, N Nwafulume, A Oke, J Taiwo (Lokoja; Federal Teaching Hospital Lokoja); OH Ekwunife, OA Egwuonwu, OA Okoye, N Nwanne, C Ugwunne, J Ugwu, U Ezidiegwu, CD Nwosu, K Oluchukwu , V Modekwe, C Uche, EA Obiesie, C Osuigwe (Nnewi; Nnamdi Azikiwe University Teaching Hospital); OH Ekwunife, J Aseme, U Edith, J Ezeh, J Nnoli, H Willy-Chidire, D Chimkaomasiri (Onitsha; Holy Rosary Specialist Hospital); A Ojewuyi (Osogbo; UNIOSUN Teaching Hospital); I Ogundele, A Adekoya, L Amosu, A Oyedele, A Ayoade, BA Ayoade, A Asekun , A Ajayi, O Popoola, M Yinusa, AAA Oyelekan, O Oluyemi, C Nwosu, M Okudero, M Agunloye, A Ojo, C Nwokoro, I Babajimi-Joseph , A Williams, S Ogunlade (Sagamu; Olabisi Onabanjo University Teaching Hospital); M Daniyan, TT Sholadoye, M Bashir, N Oyelowo, SE Nwabuoku , A Yakubu, O Ogunsua, M Abubakar , MA Tolani, MS Aliyu (Zaria; Ahmadu Bello University Teaching Hospital). North Macedonia: T Risteski, V Naunova, L Jovcheski (Skopje; University Clinic for Pediatric Surgery). Oman: PW Haque, A Albatanony, A Fajardo, M Omer, DP Talreja, AM Elsayed , H Habiba, H AlAamri (Ibra; Ibra Hospital); F Ali, S Alsibai, B Dawud, J Albalushi , AM Al Balushi, A Alzadjali, T Al Barhi, A Alkharusi, F Alfarsi, K Al Hinai, H Al Qadhi, R Al Shehhi, H Al Miskry, O Al Hamdani, O Al Alyani, B Rehmani , D Ghosh, M Al-Attraqchi, Z Al Balushi (Muscat; Sultan Qaboos University Hospital); M Al Hinai, S Alwardi, L AlRiyami, A ALJamoudi, M Masaaod (Nizwa; Nizwa Hospital). Pakistan: A Raza, A Hai, S Ahmed, DS Maqsood, S Chaudhary, M Farooq, M Tayyab, Z Qureshi, M Ali, S Maqbool, U Abdullah, M Aziz, A Irshad, S Said, A Zafar, U Akram, I Sadiq, A Abbas, M Siddique , I Shahbaz (Islamabad; Dr Akbar Niazi Teaching Hospital); SH Waqar, M Raheem, F Akhtar, DM 27 Mehmood, DN Mahmood (Islamabad; The Pakistan Institute of Medical Sciences); M Shahid, A Ali, M Ahmed, M Mansoor Iqbal, Y Lakdawala, S Otho, M Khalid, M Masood, R Kumar, S Jabeen (Karachi; PAF Faisal Hospital); S Altaf (Karachi; Patel Hospital); M Abdullah, G Shamsi, I Ahmed , N Lodhi, G Awais, L Rai (Karachi; The Indus Hospital); S Khattak, A Janjua, A Liaquat, M Saleem (Lahore; Bahria International Hospital, Bahria Orchard); MN Rafique , MM Bin Khalid, H Ahmad, MS Khalid, MH Sadiq, A Hashmi , HH Shahid, MA Sadiq, MH Chishti, M Usama, M Kashif, AM Choudhary , H Basharat, K Khalid, MA Haider, MA Bashir, H Sabir, MF Tarar, M Usama (Lahore; Services Hospital Lahore); MB Mirza, WU Rehman, W Tahir, R Khalid, CE Azmat (Lahore; The Children’s Hospital & The Institute of Child Health Lahore); H Qayum, H Irfan Khan, A Mustafa (Peshawar; Mercy Teaching Hospital); HW Bhatti, MR Farooqui, F Rauf, NA Malik (Rawalpindi; Benazir Bhutto Hospital); M Usman Malik, S Hayat, D Riazhussain (Sargodha; District Headquarter & Teaching Hospital - Sargodha). Palestine: J Najajra, N Al-Hroub, M Abu Daoud, R Jubran, M Srour, M Taamreh, A Alsalahat, H Masalma (Bethlehem, West Bank; Beit Jala Governmental Hospital (Al Hussein)); A Alwali, A Alwali, L Mohammed, M Al zebda, S Mahdi, A Shaheen, G Alrayyes, L Tafesh, T M. Abubasheer, M Abu Jayyab, H Jaber, M Abuwarda, B M. J. Alhaj, T Aldirawi, F Mahmoud, M Ali, A Albhaisi, M Obaid, WJN Almadhoun, A Alroobi (Gaza; Al-Shifa Hospital); M Abo Abdo, A Abuthaher, A AbuNemer, M Abu Al Amrain, I Nasser, A Awad, A AlAgha, R Madi, D AbuNemer (Gaza; Nasser Hospital); N Kullab, H Elhallaq, A Abu Tair (Gaza; Palestine Red Crescent Society - Al-amal Hospital); H Ayesh (Hebron, West Bank; Al-Ahli Hospital); H Abu-Arish, A Zamareh, M Ahmoud, M H. Oweidat, I AlJada, M Anati, S Halabi, W Alhroub, A Abuhammad, S M. Udwan, H Yaghmour (Hebron, West Bank; Governmental Hebron Hospital-Alia); D Houmran , NA Awwad, M Abed, I HajMohammed, A Hewari, A Alqerem, E Zidan, H Abbadi, S Abed (Jenin, West Bank; The Martyr Dr. Khalil Sulaiman Hospital (Jenin Governmental Hospital)); M Shakhshir, M HajHamad , M Saifi, S Abuzahra, A Khouli, Z Shabello, Z Khraim , S Ismail, MF Dwikat (Nablus, West Bank; Rafidia Hospital); R Bassam, A Sabbah, A Gharib, R Alzughayyar , R Issa, A Abuhantash, O Matar, Y A. Omar, O Khalil, A Awwad (Tulkarm, West Bank; Martyr Thabet Thabet Govermental Hospital). Paraguay: A Rodriguez Gonzalez, ED Sosa Ferreira, R Ferreira Acosta , MN Martínez Bareiro, JE Giubi Bobeda, A Franco (Asuncion; Hospital de Clínicas, II Cátedra de Clínica Quirúrgica, Universidad Nacional de Asunción). Peru: A Walfor (Huánuco; Hospital Regional Hermilio Valdizán Medrano); L Poggi, L Poggi, L Fuentes Rivera Lau, MA Moreno Gonzales, F Camacho, O Ibarra, G Arredondo (Lima; British American Hospital); K Nieto Yrigoin, D Chavez Fernandez, DC Juan Carlos (Lima; Clinica Internacional); G Mendiola, A Salazar, R Casma Bustamante (Lima; Hospital Santa Rosa de Lima); G Borda-Luque, Y Carpio Colmenares, MR Li Valencia, F Palomino Escalante, K Quispe de la Roca (Lima; SANNA - Clínica El Golf); MM Caramantin Obando (Paita; Hospital I Miguel Cruzado Vera EsSalud); R Polo, V Serna-Alarcon (Piura; Jose Cayetano Heredia III Regional Hospital). Poland: R Mazurkiewicz, M Kołomańska, PJ Milewski (Kielce; Wojewódzki Szpital Zespolony w Kielcach); M Kisielewski, T Stefura, K Richter, W Wysocki, N Kłos , W Jabłoński, I Alsoubie, B Żaczek, T Wojewoda, J Bolanowski (Krakow; 5th Military Clinical Hospital); Z Orzeszko, M Wikar, R 28 Solecki, B Markowska, M Szura, T Gach (Krakow; Brothers Hospitallers Hospital); M Matyja, B Habrat, W Serednicki (Krakow; Jagiellonian University Medical College); Z Lorenc, M Święch, M Mietła, W Krawczyk, M Nycz (Sosnowiec; Wojewódzki Szpiital Specjalistyczny nr 5 im. Św Barbary); K Urbańska, K Komorowska, P Kowalewski, M Walędziak (Warsaw; Military Institute Of Medicine); JK Zajac, M Zawadzki (Wroclaw; Regional Specialist Hospital in Wroclaw); M Kusiński, M Pryt, F Brzeszczyński , H Dąbrowski, M Redynk (Łódź; Copernicus Memorial Hospital). Portugal: J Figueiredo, B Cismasiu, R Souto, S Henriques, AL Preto Barreira, J Vaz, JM Carlos, M Trindade, L Moreira, M Palas, J Simoes (Almada; Hospital Garcia de Orta); F Ramalho de Almeida, M Vasconcelos, A Neves, J Ribeiro, F Afonso , A Pita, R Miranda Pera, M Bernardo, C Rio Ferreira, T Branco, J Fontaínhas, S Pimentel Morais, B Pinto (Amadora; Hospital Prof. Doutor Fernando Fonseca, E.P.E.); S Patrocínio, L Moniz, C Rolo Santos, P Bernardo, F Nazareth (Barreiro; Centro Hospitalar Barreiro Montijo, EPE); C Silva, L Heeren, AR Mateus Loureiro, B Tinoco, A Abreu (Caldas da Rainha; Hospital das Caldas da Rainha - Centro Hospitalar do Oeste, E.P.E); DM Gonçalves Múrias Gomes, C Figueiredo, C Aguero, M Reia, M Guerrero, MA Fernandez Romero, J Dominguez (Elvas; Hospital Santa Luzia Elvas); I Colaço, M Nunes Luís, S Andrade, S Oliveira, D Pais (Figueira da Foz; Hospital Distrital da Figueira da Foz); DG Alves, F Castro, R Ribeiro, I Mogárrio, MDC Gama Caldeira (Funchal; Hospital Dr. Nélio Mendonça); B Gama, CS Rodrigues, A Cabral, A Silva (Horta; Hospital da Horta, E.P.E.); E Borges, J Cassiano Neves, R Bernardino, P David Santos, J Secchi (Lisbon; Centro Hospitalar Lisboa Norte); M Nunes, D Tavares, M Cruz , C Quintela, C Cardoso, IM Lourenço (Matosinhos; Unidade Local de Saude de Matosinhos - Hospital Pedro Hispano); D Vaz Acosta, P Rego Ponte, R Santos Pereira (Ponta Delgada; Hospital do Divino Espírito Santo); H Capote, MB Mourato, T Mogne, N Andrade, G Fialho, F Valente Costa Pinto, B Cordeiro, M Brito, G Santos, D Rosado, C Costa, N Pratas (Portalegre; Hospital Doutor José Maria Grande); J DiasFerreira, AL Carreira-Marques, R Ribeiro Dias, B Carvalho, M Gomes, C Soares-Aquino, F Gomes, S Barbosa Castelo Branco, C Coutinho, JP Vieira de Sousa, D Atouguia (Porto; Centro Hospitalar e Universitário de São João); L Cidade Costa (Porto; Hospital da Prelada); D Silva, P Correia, C Henriques, AM Pinheiro Pereira, J Marques Antunes (Santa Maria da Feira; Centro Hospitalar Entre o Douro e Vouga); H Devesa, R Barradas, S Fortuna Martins, N Marcos, A Jarimba, B Louro, L Rodrigues Madeira, AR Lourenço (Santarem; Hospital de Santarem); A Ferreira, A Abreu da Silva, D Stoian , M Ferreira (Santiago do Cacém; Hospital do Litoral Alentejano); R Branquinho, JC Domingues, MI Seixo, R Lalanda, C Bôto (Tomar; Centro Hospitalar Médio Tejo); J Fernandes, P Laranjo , M Reis, I Borges da Costa, C Assis, B Lopes Patrício, NM Freitas Oliveira (Vila Franca de Xira; Hospital Vila Franca de Xira); M Carvalho, J Mendes, C Macedo Cardoso de Oliveira, B Freire, R Pinheiro Duque (Vila Nova de Famalicao; Centro Hospitalar do Medio Ave); B Vieira, U Fernandes, A Dupont, J Ribeiro, R Vaz Pereira (Vila Real; Centro Hospitalar de Trás-os-Montes e Alto Douro, E.P.E.). Qatar: G Sarp, E Soyer Güldoğan (Doha; Turkish Hospital). Romania: É Gáspár (Brasov; Regina Maria); A Chitul, C Bezede, E Ciofic, D Cristian (Bucharest; Coltea Clinical Hospital); EA Toma, IM Matache, O Enciu, B Bogdan-Gabriel (Bucharest; Elias Emergency Hospital); I Negoi, C Ciubotaru, I Tanase, C Dina, VM Negoita, A Perja (Bucharest; 29 Emergency Clinical Hospital Bucharest); R Drasovean, A Trif, D Misca, C Hossu, I Imihteev (ClujNapoca; Medicover Hospital Cluj). Russian Federation: V Kakotkin, M Agapov, V Budyakova, S Dos Santos Rocha Ferreira, R Senin (Kaliningrad; Immanuel Kant Baltic Federal University, Regional Clinical Hospital); A Bedzhanyan, A Sumbaev, K Petrenko, E Bedzhanyan, E Tyurina, R Azimov, P Glushkov, K Shemyatovsky , S Husanov, A Sidorova (Moscow; Petrovsky National Research Centre of Surgery); G Yarovenko, E Shestakov, O Lisin, A Arustamyan, S Katorkin (Samara; Hospital Surgery Clinic of Samara State Medical University); J Sidorovskaia, K Cholah, I Cholah, D Kurochka (Simferopol; Municipal Emegency Hospital No.6); V Ten, Y Kudryavcev (Yuzhno-Sakhalinsk; Private healthcare institution ‘RZD-Medicine’). Rwanda: JP Rugambwa, CN Nelly Rosine, N Jeannette, A Dusabimana (Huye, Gisagara; Butare university teaching hospital (CHUB)); C Seneza , C Uwakunda, L Mukamazera, G Ntwari, I Didier (Kigali; Kibagabaga Hospital); A Costas-Chavarri, M Eugene, C Nyampinga, R Munyaneza, D Muyenzi (Kigali; Rwanda Military Hospital). Saudi Arabia: N Alzerwi, M Rayzah, A Almutairi, A Alsultan, B Ali (Al-Majmaah; King Khalid General Hospital); A Shabkah, O Ibrahim, H Said, A Alhebshi, A Mohsen, K Anaam, F Alnazawi, F Haddad, A Basalim, B Albaihani, S Al Athath, A Jowharji, A Aljahdali (Jeddah; International Medical Center); N Trabulsi, M Alharthi, A Farsi, M Ghunaim, A Nawawi, A Maghrabi (Jeddah; King Abdulaziz University Hospital); N Alzerwi, Y Aldebasi , F Al Abbood, A Elkhalifa, M Alshanwani , S Alshagrawi (Riyadh; King Salman Hospital in Riyadh); F Ahmad (Riyadh; King Saud Medical City); A Alayed, R AlQahtani, R Sugair, O Alruwaili, B Alsharari, H Albalawi, H Al SOHABI, A Alshahrani , S Asiri, M Alshehri, A Albalawi , R Alatawi , T Khewater, H Adi, J Akiely, N Musawa, F Alahmad, B Alqahtani, M Sersarah, Z Farraj, DY Alalawi (Tabuk; King Salman Armed Forces Hospital); A Alzahrani , N Al Amri, M AlThomali, D Elkafrawy (Taif City; King Faisal Medical Complex). Serbia: J Juloski, V Cuk, V Cijan, L Milic (Belgrade; Zvezdara University Medical Center). Slovenia: JA Košir, J Grosek, A Tomazic, T Košir Božič (Ljubljana; University Medical Centre). South Africa: S Gumede, C Kloppers, K Booyse, S Dos Santos, M Flint (Cape Town; Groote Schuur Hospital); Z Johnson, JJ Jordaan, G Steenkamp (Cape Town; Karl Bremer Hospital); K Nieuwenhuys, J Uys, SS Verhage, A Goliath, S Gilbert (Cape Town; Khayelitsha District Hospital); M Kariem, N Karimbocus (Cape Town; Mitchell’s Plain District Hospital); C Lategan, T Mabogoane (Cape Town; Victoria Hospital Wynberg); S Mewa Kinoo, R Naidoo, N Ntanzi, S Sibiya, S Ebrahim (Durban; King Edward VIII Hospital); S Govender, E Naidoo, P Moodley, K Maharaj (Durban; Stanger Hospital); H Le Roux, J Van Niekerk (East London; Cecilia Makiwane Hospital); A Sparke, P Omwansa (East London; Frere Hospital); CA Baars, S Marawu, K Sevnaran, A Szpytko (Empangeni; Ngwelezana Hospital); G Charalambous, B Van Zyl, O Pheiffer, F Roodt (George; George Hospital); D Rattray, N Rasool, M Nkogatse , R Mackay, G Urdang (Johannesburg; Edenvale); V Manchev, D Clarke, S Naidu, V Govindasamy (Pietermaritzburg; Edendale Hospital); D Montwedi (Pretoria; Kalafong Academic Hospital); LC Kolongi , C Elliot-Wilson, S Kalenga , I Serfontein, M Goga (Upington; Dr Harry Surtie Hospital); S Burger, R Duvenage (Worcester; Worcester Provincial Hospital). 30 Spain: H Aguado López, F Ruescas, A García Marín, M Scortechini, M Jurado Román , A Sanchez Gallego (Albacete; Hellín Hospital); E González Marín, S De la Cruz Ahufinger, M Mateu, MJ Medina, MDM Martí-Ejarque, R Soliva Domínguez, L Ruiz-Villa, E Montalbán Martínez (BARCELONA; Hospital Universitari Sagrat Cor); A Torroella, C Ginesta, G Cárdenas Rivera, VE Gonzabay, JD Acevedo Parrales (Barcelona; HM Nou Delfos); M Canals Sin, A Lombardero, B Capdevila Vilaró, M Carbonell Pradas, ME Muñoz Fernández , RA Hernandez Rodriguez, I De Haro Jorge, M Riba Martínez, L Tapia Moral, M Coronas Soucheiron, P Palazon Bellver, L Ortega Lechuga, X Tarrado, A Domenech Plana, J Prat-Ortells, M Bejarano Serrano, M Cuesta Argos, MP Martin Gimenez, SG Laura, R Ripoll i Palmés (Barcelona; Hospital Sant Joan de Deu); A Sainz Lete, JC Zevallos-Quiroz, D Gómez, B Estraviz, JM De Francisco Rios, J Barrutia Leonardo, M González de Miguel (Bizkaia; Hospital Urduliz); RL Ferlini, M Ortega Escudero, Y Galvañ Félix, C Hernandez Diaz, J Montero García (Burgos; Hospital Universitario de Burgos); Á Fernández Camuñas, EP Garcia Santos, FJ Redondo Calvo (Ciudad Real; Hospital General Universitario de Ciudad Real); M Estaire Gómez, RJ Castro Lara, A Ramos Bonilla, L Rodríguez Gómez, M Marqueta De Salas, A Alvarez Cuiñas, FM Bujalance Cabrera, MD Cancelas, A García Domínguez, G Chamoso Mialdea, EP Cagigal Ortega, D Enjuto, I Cervera (Leganés; Severo Ochoa University Hospital); R Villalobos Mori, Y Maestre González, C Gas, L Codina Corrons, C Semeraro (Lleida; Hospital Universitari Arnau de Vilanova); J García-Quijada, TW Jorgensen, L Marquez (Madrid; Hospital Central de la Cruz Roja San Jose y Santa Adela); MJ Peña Soria, N Tabatabaian , JL Garcia galocha, D Fra Corral, L Sante Serna (Madrid; Hospital Clinico San Carlos); M Diez Alonso, C Vera Mansilla, L Casalduero, S Soto Schütte, Y Allaoua (Madrid; Hospital Universitario Principe de Asturias); E Gutierrez, C Zapata Syro, F Prieto La Noire, MDM Olmedo Reinoso, S Salido, N Chavarrias, M Vicario Bravo, L Asensio Gomez, R Abad, A Gegúndez Simón, PC Arteaga Asensio (Madrid; Hospital Universitario la Paz); AM Minaya Bravo, E González , A Galvan, C Guijarro Moreno, G De la Peña González, A Sánchez Gollarte, A Robin Valle de Lersundi, MÁ García Ureña (Madrid; Hospital del Henares); JL Rodicio Miravalles, AA Suárez Álvarez, DW Silva-Cano, G Martínez Izquierdo, P Del Val Ruiz, M Moreno Gijon, S Amoza Pais, GP Ibero Casadiego , E López-Negrete Cueto, J Carrizo, S Sanz, R Rodríguez-Uria, A Cembellin, G García-Santos, A Fraile (Oviedo; Hospital Universitario Central de Asturias (HUCA)); D Córdova García, L Jiménez, J Martin Fernandez, R Alvarado Hurtado, AM Minaya Bravo, R Díaz Pedrero, N Cobeño Tamayo, V Ongil Rodríguez (Rivas-Vaciamadrid, Madrid; Hospital Universitario HM Rivas); F Aguilar del Castillo, Á De Jesús Gil, S Borrego Canovaca (Sevilla; Hospital Universitario Virgen del Rocio); JR Naranjo Fernández, Z Valera Sanchez, R Perez, M Infantes Ormad, M Sánchez Ramirez (Seville; Hospital Universitario Virgen Macarena); C Leal Ferrandis, C Esteo Verdu, S García López, J Febré, B Cuneo (Valencia; Hospital Arnau de Vilanova); C León-Espinoza, E Martí Cuñat, G Pou (Valencia; Hospital Clínico Universitario de Valencia); C Jezieniecki, S Alonso Marcos, A Vazquez Fernandez, J Beltrán de Heredia, B De Andrés-Asenjo, D Baños Méndez, JC Garcia Vera, M Ruiz Soriano, E Redondo, R Martínez Díaz , T Gómez Sanz, P Artigot, C Infante, C Ferreras García, LR Cabezudo, G Cabezudo, M Lainez Escribano, M Rodriguez-Lopez, H Nuñez Del Barrio, A Romero de Diego (Valladolid; Hospital Clínico Universitario de Valladolid); A Vazquez Melero, M Camuera, I Herrero, D Garcia López de Goicoechea, M Sánchez-Rubio (Vitoria-Gasteiz; Hospital 31 Universitario Araba); MDP Cebollero, JL Blas Laina (Zaragoza; Hospital Royo Villanova); V Duque Mallén, N Sánchez Fuentes, P Sancho Pardo , I Gascon Ferrer, MÁ Dobón Rascón, T Gimenez Maurel, J Chóliz, S Saudí-Moro, S Paterna -Lopez, A Martinez German, D Aparicio-López, MÁ Gascón Domínguez, P Royo Dachary (Zaragoza; Hospital Universitario Miguel Servet). Sri Lanka: S Srishankar, SPB Thalgaspitiya, KJ Senanayake, D Wickramarathna (Anuradhapura; Teaching Hospital Anuradhapura); D Subasinghe, D Wickramasinghe (Colombo; National Hospital of Sri Lanka); M Nandasena, K Wijesinghe, H Miyasika, J Senavirathna, Y Chamara (Dehiwala; Colombo South Teaching Hospital); S Rajendra, SI Thuraisamy Sarma, B Balagobi, V Sutharshan , S Giridaran (Jaffna; Teaching Hospital, Jaffna); W Wijenayake, MT Ekanayake, S Jayatilleke, S Jayasekara, M Perera, R Perera, R Ellawala, WDD De Silva (Werahera; University Hospital, Kotelawala Defence University). Sudan: S Alqurashi , N Rajab (Ed Dueim; Ed Dueim Teaching Hospital); TA Albushary , FA Mohammed Daoud , A Younis, MA Suliman, F Tahir Lwdie, S Ibrahim Tour Harakan, M Abdelhadi Suliman Adam , E Hegab, A Abdalla, T Almahdi , E Alabed (El Geneina; El Geneina teaching Hospital); M Ahmed, S Eldirdiri, M Salah, OA Eljizoly , A Mohammed, A Mohamed Ibrahim Mohamed (Gadarif city; Gadarif teaching hospital); A Albager, MA Ismael Alamin, M Alsalawi, MA Elgak (Kassala; Police hospital); EG Nubi Mohamed, A Eltahir, E Adel Hamdoun Aziz, I Adel, O Emadeldeen, OG Nubi, E Mohamed (Khartoum; Bashair Teaching Hospital); M Hamed, M Tageldin, E Elsheikh, U Omara, E ADAm, IMG Ahmed, GMG Ahmed, S Imam (Khartoum; Ibrahim Malik Teaching Hospital); AA Adam, S Amin Omar Alsiddig, A Ahmed, S Abdelrasoul Elnour Ismail, M Mohamedshafee, Y Mohamed (Khartoum; Omdurman Teaching Hospital); EE Abuobaida Banaga Hag El Tayeb, H Abuobaida , AS Ahmed (Khartoum; Ribat university hospital); A Elbalal, IMG Ahmed, GN El Hunjul, GMG Ahmed, A Mustafa, HA Fadlalmola (Khartoum; Soba University Hospital); A Mohammed, E Yousuf, E Hamed, S Ibrahim, O Morgan, N Omer (Wad Madani; University of Gezira Hospital). Sweden: M Zaigham, A Al Mukhtar (Malmö; Skåne University Hospital); M Nikberg (Vasteras; Västmanlands Hospital Västerås). Switzerland: D Fenner, D Salinovic (Frauenfeld; Spital Thurgau AG); X Papazarkadas, TV Pham, C Brasset, A Litchinko, F Ris, C Golliez, M Chevallay (Geneva; Geneva University Hospitals); J Gass, J Mühlhäusser, J Metzger, A Scheiwiller (Luzern; Luzerner Kantonsspital); A Tampakis, C Riboni, U Dietz, E Brolese, C Seiler, M Kalisvaart, JN Marx, L Eisner (Olten; Kantonsspital Olten); G Peros, F Solimene, M Gramellini, A Lareida, M Adamina, E Betz, L Dubs, K Geiger-Timm, L Gantner, N Seeger, K Richetti, K Hofmann (Winterthur; Kantonsspital Winterthur); MA Schneider, D Gero, K Lehmann, P Limani, S Hügli, S Gerdes, F Mazzola, A Hiller (Zurich; University Hospital of Zurich). Syrian Arab Republic: MA Farho, M Mohammad, AY Arnaout, Y Nerabani, Y Maktabi, W Alsado, A Anadani (Aleppo; Abd Al Wahab Agha Hospital); M Morjan, M Nasani, W Mayo, S Kreid, M Arnaout, MN Sawas (Aleppo; Al-Shahbaa Private Hospital); M Aloulou, MK Marawy, A Kezze, I Arnaout, A Kelzia, A Ghazal (Aleppo; Aleppo Private Hospital); A Ghazal, E Dabbagh, R Masri, MH Nabhan, A Alniemi, A Alhaj, S Ward, Y Haido, N Dadoush, MK Abu albahrain, A Niazi, W Abbas, A Hasan, S Alshab, S Kamari, R Kalouk, Z Toutounji (Aleppo; Aleppo University Hospital); D Sharl Ajami, B 32 Alsaid, A Alusef, K Abo zaal (Damascus; Al assad university hospital); AR Hammadieh, Z Klib, MR Mslmani, A Alhaj zain, M Klib, L Shammas, AJ Chekfa, Z Odeh, R Joumaa, H Al-zoubi (Damascus; Al-Mouwasat University Hospital); Q Mashlah, HO Odah Bashi̇, H Zwaraa (Damascus; Children’s University Hospital); I Adham, L Hasan, A Khatib, S Jomaa, A Alfarwan, A Torbey, A Rashid, A Hawarah, A Ali, M Alhimyar, L Al-Boukhari, MA Al-yusuf, AN Aldirani (Damascus; Damascus Hospital); Y Alhammoud , Y Al-Junaidi (Homs; Al-Basel Specialized Hospital in Karm El-Louz); M Daher, Z Asaad, A Abbas, K Khalil (Homs; The Military Hospital); J Khoury, L Hasan, J Jahjah, J Alaji, S Turkmani, S Mahfoud, A Ahmad, M Derattani, G Massarra , J Skaff, ZA Hannouneh, H Amoudi, ZA Zaher, G Zaza (Latakia; Al Saydeh Surgical Hospital); A Wassouf, A Alahmad Alismael , S Nofal, A Mansour, M Mansour, Z Alkhaier, J Suliman, M Sabboh, M Haj Hussein, M Ibrahim, ZA Abo alaros, G Alhadwah , N Kheyrbek, D Ibrahim, G Hamdan, Y Hasan, A Abo al Shamat, A Roumieh, B Khattab, H Alkhatib (Latakia; National Hospital); S Hassan, S Abdul Rahman, A Abdul Rahman, F Aliskander, J Alkharish, G Kafa, I Suleiman, A Bassma, A Alloush , N Ismaiel, J Deeb, M Alrantisi, E Salloum, A AlMouahhed , A Baydoun, H Yunes, S Alkadi, F Ali, D Abdulrahman, I Hussein (Latakia; Othaman Hospital); A Bakri, H Asaad, T Ashkar, H Daaboul, A Marouf, F Chahrour, B Ranjous, B Ibrahim , A Sinjab, M Alneasan, N Ali, A Alloush, J Fahed, R Attaf, S Kanaan (Latakia; Tishreen University Hospital). Thailand: A Tansawet, W Kasetsermwiriya, I Laopeamthong, P Sukhvibul, T Techapongsatorn, N Techapongsatorn, P Kasetsermwiriya , P Leungon (Bangkok; Vajira hospital). Togo: PS Tekam Wadje (Lomé; CHU Sylvanus Olympio). Tunisia: MJ Kacem, R Elaifia, Y Ouadi, S Megdiche, Y Jedidi (Tunis; La Rabta Hospital). Turkey: AÇ Bozkurt, H Tümer (Adana; Adana Seyhan State Hospital); MA Koç, A Çakmak, AF Kocaay, KY Türker, TB Türkmen (Ankara; Ankara University Medical School); O Yalkin, D Yigit (Bursa; Bursa City Hospital); B Yigit, A Aslan, S Yilmaz (Elazig; Elazig Fethi Sekin City Hospital); AN Sanli, Yİ Tandoğan, A Yildiz, A İsler, A Ozkomec (Gaziantep; Abdulkadir Yuksel State Hospital); ME Seker, E Ay, M Erkaya, YO Koyluoglu, A Develioğlu, GM Kurtoglu, GK Kurtoglu, AF Cetişli, E Tunçcan, A Aghayeva, Z Durna, B Baca (Istanbul; Acibadem Altunizade Hospital); AE Dönmez, B Togay, E Ada, IE Yavuz , IA Bilgin, EC Karabulut, AM Uysal, Y Karataş, B Ağca, MK Aktas, F Demiral, B Duman, K Kabulov, I Hamzaoglu, T Karahasanoğlu, M Tanal, E Tuzuner (Istanbul; Acibadem Maslak Hospital); S Meriç, M Tokocin, H Yigitbas, A Barcin, G Alici, E Yavuz, OB Gülcicek, N Bugdayci , K Özdoğan, I Çakir, YE Aktimur, Y Altinel (Istanbul; Bagcilar Research And Training Hospital); ÖP Zanbak Mutlu (Istanbul; Bahçelievler State Hospital); RE Sönmez, M Şermet, MS Ozsoy, H Baysal, F Buyuker (Istanbul; Istanbul Medeniyet University, School of Medicine); M Oncel, S Bektas, AE Askin, M Yashar, A İzgi̇ş (Istanbul; Istanbul Medipol University Hospital); SS Uludağ, MF Ozcelik, S Yumurtacilar (Istanbul; Istanbul universty - Cerrahpaşa Medical faculty); A Özcan, E Somuncu, S Yilmaz, A Sapmaz, H Bolukbasi, C Özkan, E Bozdağ, MC Kizilkaya, H Telci, Y Kara, AZ Kaan, M Acar, EO Yildirim (Istanbul; Kanuni Sultan Suleyman Training and Research Hospital); G Akcakoca, C Hacialioğlu, Y Tosun, V Çalik, TE Yilmaz, H Alfakeer (Istanbul; Kartal Dr. Lutfi Kirdar Training and Research Hospital); MI Ateş, SN Karahan, M Kalender, AE Narin, D Yi̇ği̇t, O Agcaoglu, S Toprak, B Celik, E Ozoran, DS Uymaz, S Yigman, E Bozkurt, A Rencuzogullari, E Balik, S Sucu 33 (Istanbul; Koç University Medical School); A Akmercan, K Oğur, A Hajali, QK Dolatzay (Istanbul; Marmara University, School of Medicine); E Unal (Istanbul; Sehit Prof.Dr. İlhan Varank Training and Research Hospital); N Kiziltoprak, MS Genç, B Özcan, Z Şenol, OF Ozkan, M Çuhadar, ED Terzi (Istanbul; Sultan 2. Abdulhamid Han Training and Research Hospital, University of Health Sciences); T Gülşen (Istanbul; Sultanbeyli State Hospital); MT Demirpolat (Istanbul; University of Health Science Umraniye Education and Research Hospital); B Citgez, H Ozsahin, C Ersavas (Istanbul; Uskudar University Faculty of Medicine, Memorial Hospital); C Bi̇li̇r, AE Boztaş Demi̇r, AD Hacioglu, G Ozyuksel, H Ulman (Izmir; Bakircay University Cigli Training and Research Hospital); AM Öztürk, B Calik, AC Yaşar (Izmir; University of Health Sciences Izmir Bozyaka Training and Research Hospital); E Colak, MA Avci, E Aybar, M Gün, AB Ciftci, MS Uyanik, ME Kara, C Akgün, AC Sari, Ö Küpçüoğlu, GO Kucuk, S Polat (Samsun; Samsun University Samsun Training and Research Hospital); N Kavak, MA Kara, G Karadeniz Cakmak, B Kum (Zonguldak; Zonguldak Bulent Ecevit University School of Medicine Research and Training Hospital); S Öztürk, B Eyduran (İzmir; University of Health Sciences Tepecik Training and Research Hospital). Uganda: B Kigwe, M Arafat (Iganga; Iganga district hospital); M Nnabagulanyi (Kigumba; Kiryandongo Hospital); S Stonelake (Luwero; Kiwoko Hospital). United Kingdom: ZA Fozo, A Shoker, KR Rahman, MW Saqib, R Faisal, CS Ong, A Pillai, H Unwin, A Huws, M Maybury, H Ejaz, E Daketsey, AK Lala (Bangor, North Wales; Ysbyty Gwynedd); MAK Sarker, B Chkir, MR Peris, S Khan , MA Tahir, N Sharma, R Doherty, H Alhusaini, MF Butt, H Afzal, S Handa, N Maharjan , A Mostafa, G Lee, CK Lim, A Anand, A Krishna, WT Yew, Y Lu, R Hall (Barrow in Furness; Furness General Hospital); E Mohammed (Basildon; Basildon University Hospital); F Georgiades, S Karim, K Rajaratnam (Bedford; Bedford Hospital); J Abbasy, A Bibi, S Karandikar, L Johnstone , N Fazili (Birmingham; Heartlands Hospital); A Singh, A Athanasiou, H Lidbetter, J Siby, M Kaur, A Fatima (Brighton; Royal Sussex County Hospital); SM Reddy, A Campbell, A Cardoso Almeida, K Smith, CJ Bradshaw, K Tambudze, H Delacave, I Norman (Bristol; Bristol Royal Hospital for Children); M San, S Babu, S Midya, H Bradly, S Tontus, H Chauhan, R Jurdon, M Corcos , E Jose (Camberley; Frimley Health NHS FT - Frimley Park); N Eardley, B Davies, M Ransome, S Ahmed (Chester; Countess of Chester Hospital); S Suresh, A Bavaharan, R Batir, R Sato (Colchester; Colchester Hospital University); N Chidumije, M Ahmed, TY Kwan, F Olaniru, I Parwaiz, LP Cheng (Coventry; University Hospitals Coventry and Warwickshire NHS Trust); N Gokhare Viswanath, K Nanayakkara, A Tibude (Derby; Royal Derby Hospital); O Olajumoke, M Shams, D George, A Amin, M Kausar, S Sellahewa (Dudley; Russell’s Hall Hospital); H Kamal, A Kamal, M Kamal (Dundee; Ninewells Hospital); O Pryer, H Sagar, B Lulham-Robinson, S Dawo, A Nada (Durham; University Hospital North Durham); R Bethune, G Chillarge, AM Myintmo, M Horga, L Andreski, E Ruiz-Daum , T Finlay, I Rakshit (Exeter; Royal Devon and Exeter Hospital); J Bryan, D Joshi, R Marlin, M Battili , YL Aung, L Zeng, S Mathew, I Njere, S Gopaul, A Abbas (Great Yarmouth; James Paget Univeristy NHS Foundation Trust Hospital); G Singh, M Asarbakhsh, S Staight (Huddersfield; Huddersfield Royal Infirmary); R Govindaraju, M Quaunine, S Yassin, A Wilkins, J Walshaw, L Chang, A Mahendran, F Hammett, D Fairbrass, UA Kalu, E Dexter, T Nadeem, N Karunaratne, T Lo, M Pellen (Hull; Hull University Teaching Hospitals NHS Trust); SH Sarwary, J Otote, D Anbu, H Islam, N Morricone (Ilford; 34 King George Hospital); K Lee, E Gimson, M Wilson, C Chiam (Larbert; Forth Valley Royal Hospital); M Solkar, M Bautista, NS Blencowe, A Gupta, J Sutcliffe, A Ahmad, A Peckham-Cooper (Leeds; Leeds Teaching Hospitals); E O’Connell, KE Dey, R Lunevicius, MM Barakat, GR Goodwin, B Devkaran, IC Nzenwa, A Pilavas (Liverpool; Aintree University Hospital); K Bananis, A Alamin, S Bennett , MD Barcelona, A Sharp, F Soggiu (London; Ealing Hospital); E Baili, H Ebied, A Botha, M Haghighat Ghahfarokhi, MMT Youssef (London; Guy’s and St Thomas’ Hospitals); K Theodoropoulou, A Quddus, R Hegy, M Mahran, A Ghanbari (London; Homerton University Hospital); P Kapsampelis, T Chouari, J Saunders, C Boven, I Gerogiannis (London; Kingston); N Karthikeyan, C Karagianni , E Spanoudakis (London; Queen Elizabeth Hospital, Woolwich); H Younus, R Ben Hmida, D Eaton (London; The Whittington Hospital); C Seet, R Bradley, R Roberts (London; University Hospital Lewisham); FN Amir, M Durrani, S Khan, A Tahir (Macclesfield; Macclesfield District General Hospital); A Khalil, E O’Neill, S Ingley, V Bill, P Wilson, M Elmousili, E Chin , C Alphonse, P Suresh, L Devi, C Shelton (Manchester; Wythenshawe Hospital); S Bugren, M Abdelreheem, MMR Azzuz, F Gareb (Margate; Queen Elizabeth the Queen Mother Hospital Margate); DJ Dhillon, MF Khan, B Peter, I Fagiri , T Harris , P Thambi (Middlesbrough; James Cook University Hospital); M Tomlinson, C Hidalgo Salinas, A Mwanjoka , M Catterall, B Ali (Morecambe; Royal Lancaster Infirmary); S Tingle, K Waddell, F Peters, T Akharaekpanya, S Robinson (North Shields; Northumbria NHS Hospital Trust); NA Binti Yusri, A Ashiru, SA Chowdhury , J Reilly, S Malek, S Kumaran, A Doghaim, ABA AlHajjaj, LR Chieng, ZY Wong, R Olatunji (Nottingham; Queens Medical Centre); J Bundred, B Down, A Ang, KA Shamiyah, G Bond-Smith (Oxford; John Radcliffe Hospital); M Elmesalmi (Portsmouth; Queen Alexandra Hospital); S Monkhouse , O Mohamed, B Robertson-Jones, P Patel, BZ Hao (Redhill; East Surrey Hospital); S Ghattas, N Beharry, A Maraqa, N Uttam, S Rafiq, A Abdelhamid, E Mazumdar, M Dyer, MEEA Abdelsalam, B Johnson, M Abdelkarim, A Murtada, MMS Tora, Z Azhar (Rhyl; Glan Clwyd Hospital); O Whitehurst, K Bhatti, S Silvestre, LN Bin Aizan, A Patel (Salford; Salford Royal Hospital); Z Khan, A Maqsood-Shah, O Webster, H Reilly, S Boyes (Sheffield; Sheffield Teaching Hospital NHS Foundation Trust); S Bandyopadhyay, B McDermott, H Kynaston, B Neall, M West, G Hart, R Titcombe , H Kaur, A Ekerin, A Demetriou, N Harrison, JJQ Chen (Southampton; Southampton General Hospital); M Hammoda, L Robine-Durnell, E Mansour, A Evans, E Baker (Swansea; Morriston Hospital Swansea); I Abdullah, O Osunlusi (Walsall; Walsall Manor Hospital). United States: S Soelling, R Askari (Boston; Brigham and Women’s Hospital); M Asaad , J Leong, M Perkins, D Ozal, SS Budhwani (Chicago, IL; Mount Sinai Hospital); A Maxwell, A Shah, S Schimpke (Chicago, IL; Rush University Medical Centre); D Moris, C Nicholson Jr., HE Rice (Durham, NC; Duke University Medical Center); D Ridder, M Tsuruta, K Noguchi, D Mikami, R Kitamura, J Ng-Kamstra (Honolulu, Hawaii; The Queen’s Medical Center); RJ Robitsek, K Fretwell, J Chan, R Laskowski (Jamaica; Jamaica Hospital); R Zerna Encalada (Syracuse; SUNY Upstate University Hospital). Yemen, Rep.: R Ghaleb, N Alnamari, A Al-Bahla, B Al soudi (Hajjah; Kowaydina hospital); B Alshaikh, M Al-Shehari, M Al-Dhaheri, YSSM Ghaleb, MI Issa (Sana’a; Al-Thawra Modern General Hospital).
