# Supplementary material for: Global anaesthesia practice using inguinal hernia surgery as a tracer condition: a secondary analysis of an international prospective cohort study
Source: Anaesthesia. 2025 Sep 9;80(11):1343–51. doi: 10.1111/anae.16686 (PMC12519944; doi:10.1111/anae.16686)
Supplement: Supplementary file 3 — Appendix S2. Reflexivity statement. [file ANAE-80-1343-s002.docx]

## **Appendix S2. Reflexivity Statement**

**How does this study address local research and policy priorities?**

This research was prioritised by the NIHR Global Health Research Group on Environmentally Sustainable Hospitals in Low- and Middle-income Countries steering group. This steering group is comprised of 35 surgical and anaesthetic researchers, with 12 from high income countries and 23 from low- and middle-income countries.

Further information on the primary study can be found in the Equitable Partnership Declaration (EPD), supplementary appendix 2.

**How were local researchers involved in study design?**

The steering group was involved in the primary cohort study and subsequently in design of this secondary analysis.

**How has funding been used to support the local research team(s)?**

The research group is co-funded with the NIHR Global Health Research Unit on Global Surgery, which funds seven research hubs in South Africa, Rwanda, Nigeria, Benin, Ghana, Mexico and India. Funding was not required for other countries that participated in this study.

The NIHR Global Health Research Group on Environmentally Sustainable Hospitals in Low- and Middle-income Countries has created free tool to enhance skills of local researchers (<https://globalsurgeryunit.org/education>)

**How are research staff who conducted data collection acknowledged?**

Hospital leads identified teams of data collectors within each hospital. All hospital leads and members of data collection teams are included in the authorship, in the supplementary materials.

**How have members of the research partnership been provided with access to study data?**

Data from the study has been presented online to the NIHR Global Health Research Group on Environmentally Sustainable Hospitals in Low- and Middle-income Countries group. We will additionally produce a template presentation, which can be used by all authors to present data locally.

**How were data used to develop analytical skills within the partnership?**

The steering group reviewed data at each stage and was involved in data analysis.

**How have research partners collaborated in interpreting study data?**

Descriptive data from the study were presented to the steering committee and interpretations discussed. The structure of the paper was planned accordingly. All perspectives were valued equally.

**How were research partners supported to develop writing skills?**

Drafts of the paper were presented to the steering group and iteratively amended through online discussions

**How will research products be shared to address local needs?**

All findings from this secondary analysis will be published through open access, within the main manuscript or the supplement.

**How is the leadership, contribution and ownership of this work by LMIC researchers recognised within the authorship?**

Given the collaborative nature of this research, all authors are categorised are listed by alphabetic order within the NIHR Global Health Research Group on Environmentally Sustainable Hospitals in Low- and Middle-income Countries

**How have early career researchers across the partnership been included within the authorship team?**

Early career researchers have been involved throughout. The first authors CL and MP are PhD candidates, and other PhD candidates and trainees have been involved in data analysis, manuscript review and editing.

**How has gender balance been addressed within the authorship?**

The steering group consisted of 25 men and 12 women.

**How has the project contributed to training of LMIC researchers?**

LMIC researchers have been a core and majority part of the steering group and data collection teams, and this has contributed to training.

**How has the project contributed to improvements in local infrastructure?**

This project has not contributed to improvements of local infrastructure

**What safeguarding procedures were used to protect local study participants and researchers?**

The main risk to collaborators was lack of allocated research time. As a result, data-collection teams were limited to 5 collaborators, which could be changed depending on local needs.
