# Supplementary material for: Global anaesthesia practice using inguinal hernia surgery as a tracer condition: a secondary analysis of an international prospective cohort study
Source: Anaesthesia. 2025 Sep 9;80(11):1343–51. doi: 10.1111/anae.16686 (PMC12519944; doi:10.1111/anae.16686)

### **Figure S1:** Multilevel logistic regression model showing odds ratios for complications against types of anaesthetic.


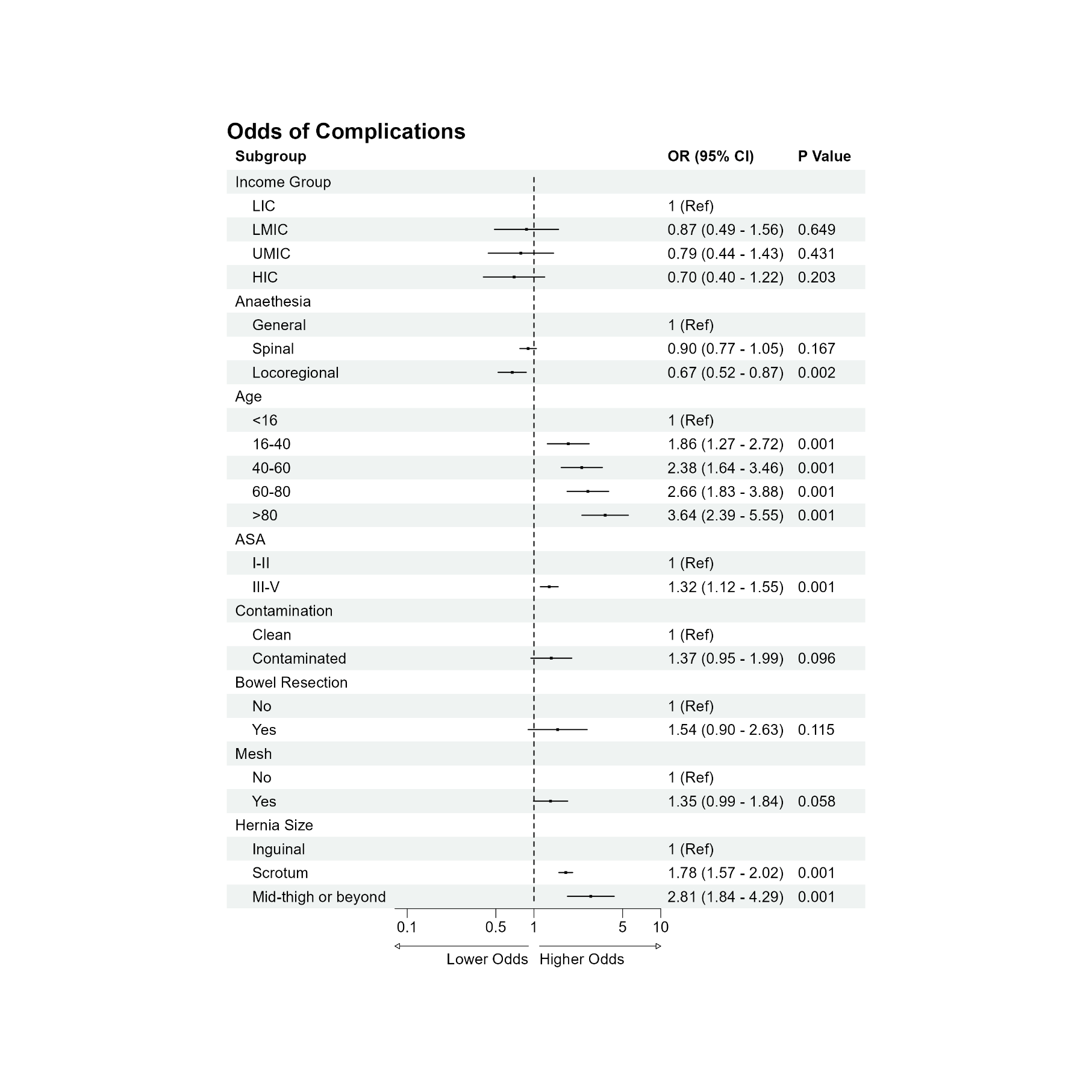


### **Figure S2:** Map showing halothane use globally. Higher use of halothane is indicated by darker shades of blue


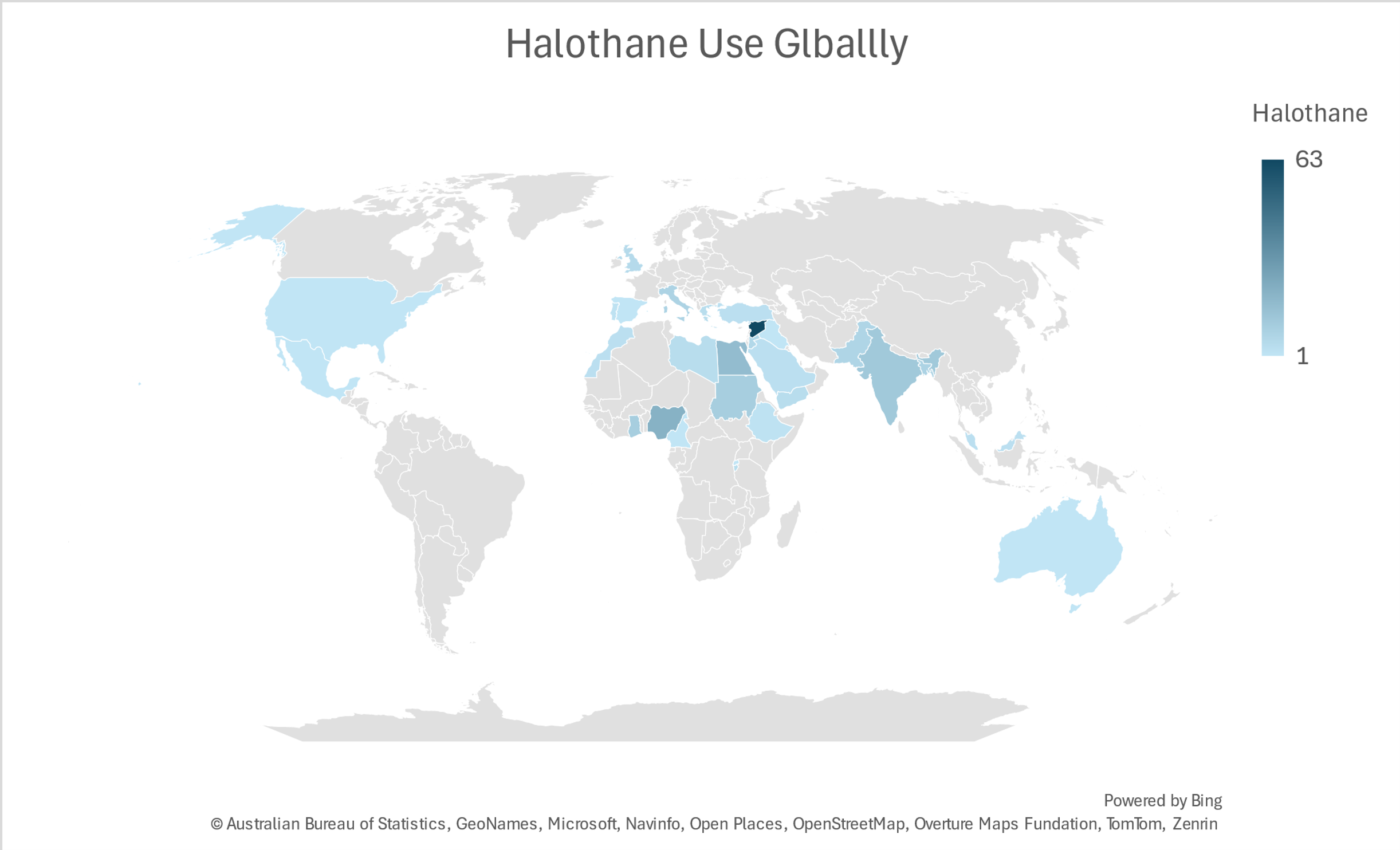

Supplement: Supplementary file 4 — Figure S1. Multilevel logistic regression model showing odds ratios for complications against types of anaesthetic. Figure S2. Map showing halothane use globally. [file ANAE-80-1343-s005.docx]
