# Supplementary material for: Global anaesthesia practice using inguinal hernia surgery as a tracer condition: a secondary analysis of an international prospective cohort study
Source: Anaesthesia. 2025 Sep 9;80(11):1343–51. doi: 10.1111/anae.16686 (PMC12519944; doi:10.1111/anae.16686)
Supplement: Supplementary file 5 — Table S1. Country and hospital characteristics across anaesthetic groups. Table S2. Country, hospital and patient selection, capacity and safety outcomes across paediatric age groups. Table S3. Subgroup table of complications in locoregional anaesthesia across anaesthesia provider, grouped by anaesthetic type. Table S4. Anaesthetic volatile agents and gases across income groups. [file ANAE-80-1343-s003.docx]

### **Table S1:** Country and hospital characteristics across anaesthetic groups; general anaesthetic broken down into “general inhaled” and Total Intravenous Anaesthetic (TIVA). Values are number (proportion)

|  |  | General Inhaled | TIVA | Spinal | Locoregional | Total |  |
| --- | --- | --- | --- | --- | --- | --- | --- |
| Income  groups | LIC | 199 (3.4) | 53 (1.6) | 491 (8.4) | 61 (4.0) | 804 (4.9) | <0.001 |
|  | LMIC | 1239 (20.9) | 143 (4.4) | 1720 (29.4) | 445 (29.0) | 3547 (21.4) |  |
|  | UMIC | 1386 (23.4) | 385 (11.9) | 1127 (19.3) | 177 (11.5) | 3075 (18.6) |  |
|  | HIC | 3107 (52.4) | 2653 (82.0) | 2515 (43.0) | 853 (55.5) | 9128 (55.1) |  |
| HDI  groups | Low | 272 (4.2) | 103 (3.0) | 647 (10.3) | 222 (14.1) | 1244 (7.0) | <0.001 |
|  | Medium | 969 (14.9) | 75 (2.2) | 1380 (22.0) | 253 (16.1) | 2677 (15.0) |  |
|  | High | 1528 (23.5) | 311 (8.9) | 1168 (18.7) | 146 (9.3) | 3153 (17.7) |  |
|  | Very High | 3736 (57.4) | 2997 (86.0) | 3067 (49.0) | 954 (60.6) | 10754 (60.3) |  |
| Hospital  Type | Primary | 498 (8.5) | 267 (8.3) | 404 (7.0) | 184 (12.0) | 1353 (8.3) | <0.001 |
|  | Secondary | 1356 (23.1) | 965 (30.1) | 1551 (26.7) | 510 (33.3) | 4382 (26.7) |  |
|  | Tertiary | 4005 (68.4) | 1970 (61.5) | 3850 (66.3) | 838 (54.7) | 10663 (65.0) |  |
| Hospital  Funding | Public | 4562 (77.9) | 2860 (89.3) | 4889 (84.2) | 1374 (89.7) | 13685 (83.5) | <0.001 |
|  | Private | 908 (15.5) | 228 (7.1) | 646 (11.1) | 98 (6.4) | 1880 (11.5) |  |
|  | Public-Private | 389 (6.6) | 114 (3.6) | 270 (4.7) | 60 (3.9) | 833 (5.1) |  |
| Hospital  Payment | Other | 219 (3.7) | 125 (3.9) | 272 (4.7) | 46 (3.0) | 662 (4.0) | <0.001 |
|  | Other insurance | 607 (10.4) | 363 (11.3) | 236 (4.1) | 79 (5.2) | 1285 (7.8) |  |
|  | Insurance by the  government | 4554 (77.7) | 2652 (82.8) | 4737 (81.6) | 1199 (78.3) | 13142 (80.1) |  |
|  | Out of pocket | 479 (8.2) | 62 (1.9) | 560 (9.6) | 208 (13.6) | 1309 (8.0) |  |

LIC – Low Income Countries, LMIC – Lower Middle Income Countries, UMIC – Upper Middle Income Countries, HIC – High Income Countries, HDI – Human Development Index

### **Table S2:** Country, hospital and patient selection, capacity and safety outcomes across paediatric age groups. Values are number (proportion)

|  |  | <1 month | 1-12 months | 1-3 years | 4-11 years | 12-18 years | Total |
| --- | --- | --- | --- | --- | --- | --- | --- |
| Anaesthetic Type | General | 209 (88.2) | 437 (94.8) | 335 (96.5) | 635 (91.0) | 71 (45.8) | 1687 (88.9) |
|  | Spinal | 19 (8.0) | 18 (3.9) | 8 (2.3) | 51 (7.3) | 63 (40.6) | 159 (8.4) |
|  | Sedation | 0 (0.0) | 0 (0.0) | 0 (0.0) | 0 (0.0) | 0 (0.0) | 0 (0.0) |
|  | Loco-regional | 9 (3.8) | 6 (1.3) | 4 (1.2) | 12 (1.7) | 21 (13.5) | 52 (2.7) |
| Income Group | LIC | 29 (12.2) | 35 (7.6) | 35 (10.1) | 57 (8.2) | 19 (12.3) | 175 (9.2) |
|  | LMIC | 89 (37.6) | 230 (49.9) | 186 (53.6) | 308 (44.1) | 76 (49.0) | 889 (46.8) |
|  | UMIC | 80 (33.8) | 128 (27.8) | 67 (19.3) | 185 (26.5) | 39 (25.2) | 499 (26.3) |
|  | HIC | 39 (16.5) | 68 (14.8) | 59 (17.0) | 148 (21.2) | 21 (13.5) | 335 (17.7) |
| HDI Group | Low | 37 (12.7) | 40 (7.6) | 37 (10.1) | 64 (8.9) | 23 (13.5) | 201 (9.7) |
|  | Medium | 110 (37.7) | 267 (51.0) | 195 (53.0) | 314 (43.4) | 84 (49.1) | 970 (46.7) |
|  | High | 95 (32.5) | 145 (27.7) | 73 (19.8) | 189 (26.1) | 40 (23.4) | 542 (26.1) |
|  | Very High | 50 (17.1) | 72 (13.7) | 63 (17.1) | 156 (21.6) | 24 (14.0) | 365 (17.6) |
| Hospital Type | Primary | 13 (5.5) | 8 (1.7) | 18 (5.2) | 30 (4.3) | 16 (10.3) | 85 (4.5) |
|  | Secondary | 21 (8.9) | 41 (8.9) | 32 (9.2) | 102 (14.6) | 32 (20.6) | 228 (12.0) |
|  | Tertiary | 199 (84.0) | 411 (89.2) | 295 (85.0) | 561 (80.4) | 107 (69.0) | 1573 (82.9) |
|  | (Missing) | 4 (1.7) | 1 (0.2) | 2 (0.6) | 5 (0.7) | 0 (0.0) | 12 (0.6) |
| Hospital Funding | Public | 193 (81.4) | 418 (90.7) | 304 (87.6) | 567 (81.2) | 133 (85.8) | 1615 (85.1) |
|  | Private | 38 (16.0) | 28 (6.1) | 33 (9.5) | 94 (13.5) | 19 (12.3) | 212 (11.2) |
|  | Public-Private | 2 (0.8) | 14 (3.0) | 8 (2.3) | 32 (4.6) | 3 (1.9) | 59 (3.1) |
|  | (Missing) | 4 (1.7) | 1 (0.2) | 2 (0.6) | 5 (0.7) | 0 (0.0) | 12 (0.6) |
| Hospital Payment | Other | 10 (4.2) | 21 (4.6) | 19 (5.5) | 33 (4.7) | 3 (1.9) | 86 (4.5) |
|  | Other insurance | 24 (10.1) | 23 (5.0) | 21 (6.1) | 45 (6.4) | 12 (7.7) | 125 (6.6) |
|  | Insurance by the government | 173 (73.0) | 370 (80.3) | 258 (74.4) | 501 (71.8) | 110 (71.0) | 1412 (74.4) |
|  | Out of pocket | 26 (11.0) | 46 (10.0) | 47 (13.5) | 114 (16.3) | 30 (19.4) | 263 (13.9) |
|  | (Missing) | 4 (1.7) | 1 (0.2) | 2 (0.6) | 5 (0.7) | 0 (0.0) | 12 (0.6) |
| Sex | Female | 29 (12.2) | 90 (19.5) | 72 (20.7) | 205 (29.4) | 14 (9.0) | 410 (21.6) |
|  | Male | 208 (87.8) | 371 (80.5) | 275 (79.3) | 493 (70.6) | 141 (91.0) | 1488 (78.4) |
| ASA Groups | ASA I-II | 230 (97.0) | 439 (95.2) | 341 (98.3) | 694 (99.4) | 153 (98.7) | 1857 (97.8) |
|  | ASA III-V | 5 (2.1) | 18 (3.9) | 4 (1.2) | 3 (0.4) | 2 (1.3) | 32 (1.7) |
|  | Not recorded | 2 (0.8) | 4 (0.9) | 2 (0.6) | 1 (0.1) | 0 (0.0) | 9 (0.5) |
| Comorbidities | None | 236 (99.6) | 451 (97.8) | 344 (99.1) | 690 (98.9) | 153 (98.7) | 1874 (98.7) |
|  | One | 1 (0.4) | 10 (2.2) | 3 (0.9) | 7 (1.0) | 2 (1.3) | 23 (1.2) |
|  | Two | 0 (0.0) | 0 (0.0) | 0 (0.0) | 1 (0.1) | 0 (0.0) | 1 (0.1) |
|  | Three or more | 0 (0.0) | 0 (0.0) | 0 (0.0) | 0 (0.0) | 0 (0.0) | 0 (0.0) |
| Hernia Size | Limited to inguinal region | 156 (65.8) | 266 (57.7) | 234 (67.4) | 510 (73.1) | 105 (67.7) | 1271 (67.0) |
|  | Limited to scrotum | 80 (33.8) | 190 (41.2) | 112 (32.3) | 187 (26.8) | 49 (31.6) | 618 (32.6) |
|  | Extend to mid-thigh or beyond | 1 (0.4) | 5 (1.1) | 1 (0.3) | 1 (0.1) | 1 (0.6) | 9 (0.5) |
| Indication groups | Asymptomatic | 99 (41.8) | 217 (47.1) | 160 (46.1) | 327 (46.8) | 27 (17.4) | 830 (43.7) |
|  | Symptomatic | 138 (58.2) | 244 (52.9) | 187 (53.9) | 371 (53.2) | 128 (82.6) | 1068 (56.3) |
| Day case | No | 94 (39.7) | 174 (37.7) | 116 (33.4) | 243 (34.8) | 71 (45.8) | 698 (36.8) |
|  | Yes | 143 (60.3) | 287 (62.3) | 231 (66.6) | 455 (65.2) | 84 (54.2) | 1200 (63.2) |
| Anaesthetic  administrator | Anaesthetist / anaesthetic nurse / technician | 218 (92.0) | 435 (94.4) | 325 (93.7) | 645 (92.4) | 128 (82.6) | 1751 (92.3) |
|  | Yes (e.g. surgeon) | 19 (8.0) | 26 (5.6) | 22 (6.3) | 53 (7.6) | 27 (17.4) | 147 (7.7) |
| Complications | no | 214 (90.3) | 426 (92.4) | 329 (94.8) | 667 (95.6) | 138 (89.0) | 1774 (93.5) |
|  | complications | 23 (9.7) | 35 (7.6) | 18 (5.2) | 31 (4.4) | 17 (11.0) | 124 (6.5) |
| Clavien Dindo | 0 (no complications) | 214 (90.3) | 426 (92.4) | 329 (94.8) | 667 (95.6) | 138 (89.0) | 1774 (93.5) |
|  | I | 18 (7.6) | 26 (5.6) | 13 (3.7) | 24 (3.4) | 13 (8.4) | 94 (5.0) |
|  | II | 3 (1.3) | 6 (1.3) | 4 (1.2) | 7 (1.0) | 4 (2.6) | 24 (1.3) |
|  | IIIb | 2 (0.8) | 2 (0.4) | 1 (0.3) |  |  | 5 (0.3) |
|  | IIIa |  | 1 (0.2) |  |  |  | 1 (0.1) |
| Post op infection | No | 223 (94.1) | 452 (98.0) | 339 (97.7) | 683 (97.9) | 148 (95.5) | 1845 (97.2) |
|  | Yes | 14 (5.9) | 9 (2.0) | 8 (2.3) | 15 (2.1) | 7 (4.5) | 53 (2.8) |
| Post op re-operation | No | 235 (99.2) | 459 (99.6) | 345 (99.4) | 698 (100.0) | 155 (100.0) | 1892 (99.7) |
|  | Yes | 2 (0.8) | 2 (0.4) | 2 (0.6) |  |  | 6 (0.3) |

LIC – Low Income Countries, LMIC – Lower Middle Income Countries, UMIC – Upper Middle Income Countries, HIC – High Income Countries, TIVA – Total Intravenous Anaesthetic, HDI – Human Development Index

### **Table S3:** Subgroup table of complications in locoregional anaesthetic across anaesthesia provider, grouped by anaesthetic type. Values are number (proportion)

|  |  | **Anaesthetist / anaesthetic nurse / technician n = 15999** | | | **Surgeon**  **n = 1835** | | |
| --- | --- | --- | --- | --- | --- | --- | --- |
|  |  | **General** | **Spinal** | **Locoregional** | **General** | **Spinal** | **Locoregional** |
| **Complications** | **No** | 8354 (87.6) | 5034 (84.1) | 405 (87.9) | 403 (89.0) | 240 (89.6) | 980 (88.3) |
|  | **Yes** | 1179 (12.4) | 949 (15.9) | 56 (12.1) | 50 (11.0) | 28 (10.4) | 130 (11.7) |
| **Clavien-Dindo** | **0** | 8354 (87.6) | 5034 (84.1) | 405 (87.9) | 403 (89.0) | 240 (89.6) | 980 (88.3) |
|  | **I** | 853 (8.9) | 741 (12.4) | 42 (9.1) | 35 (7.7) | 21 (7.8) | 97 (8.7) |
|  | **II** | 214 (2.2) | 159 (2.7) | 12 (2.6) | 10 (2.2) | 5 (1.9) | 24 (2.2) |
|  | **IIIa** | 40 (0.4) | 25 (0.4) | 1 (0.2) | 3 (0.7) | 2 (0.7) | 8 (0.7) |
|  | **IIIb** | 50 (0.5) | 19 (0.3) | 0 | 2 (0.4) | 0 | 1 (0.1) |
|  | **IVa** | 6 (0.1) | 1 (0.0) | 1 (0.2) | 0 | 0 | 0 |
|  | **IVb** | 4 (0.0) |  | 0 | 0 | 0 | 0 |
|  | **V (death)** | 12 (0.1) | 4 (0.1) | 0 | 0 | 0 | 0 |
| **Post-op infection** | **No** | 9271 (97.3) | 5721 (95.7) | 434 (94.1) | 436 (96.2) | 255 (95.1) | 1074 (96.8) |
|  | **Yes** | 262 (2.7) | 260 (4.3) | 27 (5.9) | 17 (3.8) | 13 (4.9) | 36 (3.2) |
| **Post-op re-operation** | **No** | 9463 (99.3) | 5951 (99.5) | 461 (100.0) | 450 (99.3) | 267 (99.6) | 1106 (99.6) |
|  | **Yes** | 70 (0.7) | 30 (0.5) | 0 | 3 (0.7) | 1 (0.4) | 4 (0.4) |

### **Table S4:** Anaesthetic volatile agents and gases across income groups. Values are number (proportion)

| label | levels | LIC  n = 199 | LMIC  n = 1239 | UMIC  n = 1386 | HIC  n = 3107 | Total  n = 5931 | p |
| --- | --- | --- | --- | --- | --- | --- | --- |
| Inhaled volatile agent | N20 | 2 (1.0) | 13 (1.0) | 7 (0.5) | 61 (2.0) | 83 (1.4) | <0.001 |
|  | Halothane | 84 (42.2) | 92 (7.4) | 18 (1.3) | 25 (0.8) | 219 (3.7) |  |
|  | Desflurane | 3 (1.5) | 8 (0.6) | 82 (5.9) | 363 (11.7) | 456 (7.7) |  |
|  | Isoflurane | 34 (17.1) | 378 (30.5) | 220 (15.9) | 83 (2.7) | 715 (12.1) |  |
|  | Sevoflurane | 76 (38.2) | 748 (60.4) | 1059 (76.4) | 2570 (82.7) | 4453 (75.1) |  |
|  | (Missing) | 0 (0.0) | 0 (0.0) | 0 (0.0) | 5 (0.2) | 5 (0.1) |  |

LIC – Low Income Countries, LMIC – Lower Middle Income Countries, UMIC – Upper Middle Income Countries, HIC – High Income Countries
